# Transcriptional profiling of host gene expression in chicken embryo lung cells infected with laryngotracheitis virus

**DOI:** 10.1186/1471-2164-11-445

**Published:** 2010-07-21

**Authors:** Jeong Yoon Lee, Joon Jin Song, Ann Wooming, Xianyao Li, Huaijun Zhou, Walter G Bottje, Byung-Whi Kong

**Affiliations:** 1 Department of Poultry Science, University of Arkansas, Fayetteville, AR 72701, USA; 2 Cell and Molecular Biology Graduate Program, University of Arkansas, Fayetteville, AR 72701, USA; 3 Department of Mathematical Sciences, University of Arkansas, Fayetteville, AR 72701, USA; 4 Department of Poultry Science, Texas A&M University, College Station, TX 77845, USA

## Abstract

**Background:**

Infection by infectious laryngotracheitis virus (ILTV; *gallid herpesvirus 1*) causes acute respiratory diseases in chickens often with high mortality. To better understand host-ILTV interactions at the host transcriptional level, a microarray analysis was performed using 4 × 44 K Agilent chicken custom oligo microarrays.

**Results:**

Microarrays were hybridized using the two color hybridization method with total RNA extracted from ILTV infected chicken embryo lung cells at 0, 1, 3, 5, and 7 days post infection (dpi). Results showed that 789 genes were differentially expressed in response to ILTV infection that include genes involved in the immune system (cytokines, chemokines, MHC, and NF-κB), cell cycle regulation (cyclin B2, CDK1, and CKI3), matrix metalloproteinases (MMPs) and cellular metabolism. Differential expression for 20 out of 789 genes were confirmed by quantitative reverse transcription-PCR (qRT-PCR). A bioinformatics tool (Ingenuity Pathway Analysis) used to analyze biological functions and pathways on the group of 789 differentially expressed genes revealed that 21 possible gene networks with intermolecular connections among 275 functionally identified genes. These 275 genes were classified into a number of functional groups that included cancer, genetic disorder, cellular growth and proliferation, and cell death.

**Conclusion:**

The results of this study provide comprehensive knowledge on global gene expression, and biological functionalities of differentially expressed genes in chicken embryo lung cells in response to ILTV infections.

## Background

Infectious laryngotracheitis virus (ILTV; *gallid herpesvirus 1*) is the only member of the *Iltovirus *genus of the *Alphaherpesvirinae *subfamily of the *Herpesviridae *family. ILTV includes ~150 kb of linear dsDNA genome consisting of two unique regions (unique long; UL and unique short; US), inverted repeats (IR) and terminal repeats (TR) flanking the US region [1]. About 76 open reading frames (ORFs) have been shown to express viral proteins in ILTV [2]. The genome structure and gene contents of the ILTV genome clearly prove its classification as an *alphaherpesvirus *[3]. Infection of ILTV causes an upper respiratory disease in chickens during lytic infection, and ILTV can establish latency in the central nervous system. Respiratory symptoms of ILTV infection include dramatically increased mucus formation in the trachea and tracheal hemorrhage that can cause up to 70% mortality. Currently, live attenuated vaccines developed from chicken embryo or cultured cells are commercially available to control ILTV disease [4]. However, vaccinal laryngtracheitis (VLT), resulting from reversion of vaccine virus to virulent form and spreading from vaccinated- to unvaccinated birds, is often associated with the use of live attenuated ILTV vaccines [5,6].

Microarray methodology was developed as an epochal method to simultaneously analyze enormous data sets for gene expression patterns in various biological conditions [7]. Microarrays have been used to investigate host responses to the infection of various viruses such as Epstein-Barr virus (EBV) [8-10], varicella-zoster virus (VZV) [11], human cytomegalovirus (HCMV) [12], Marek's disease virus (MDV) [13-17], herpesvirus of turkey [18], herpes simplex virus-1 (HSV-1) [19,20], hepatitis virus [21], human immunodeficiency virus (HIV) [22-25] and coxsackieviruses [26].

Microarray data sets can be interpreted further by clustering analysis. Many of the heuristic clustering methods have several shortcomings; these include the determination of the number of clusters which generally is unknown when there is no prior knowledge of the number or there no other information about the structure of the data to be clustered. A model-based clustering method can overcome these critical drawbacks by treating a clustering problem as a model selection problem over a variety of candidate models specified by different numbers of clusters and distribution, and by estimating the number of clusters in the clustering analysis. The best model is selected on the basis of a model selection criterion, simultaneously providing the optimal number of clusters and assigning cluster membership to observations.

To our knowledge, the effects of ILTV infection on changes in global gene expression in host cells have not been previously reported. ILTV is a special type of herpesvirus that causes acute respiratory disease in poultry. Thus, the objective of this study was to understand host responses to ILTV infection in cultured chicken embryo lung cells using microarray analysis. The microarray used in the current study contains 44 K chicken genes including functionally identified genes, predicted ORFs, ESTs, genomic contigs, chicken microRNAs and various control spots [27]. Importantly, functional analysis of differentially expressed genes should follow gene discovery research. To this end, a software program (Ingenuity Pathway Analysis), specifically developed to analyze large data sets such as microarray data for biological functionalities, gene networks, and physiological pathways [28], was used to assign biological functionalities and molecular interactions in chicken embryo lung cells after 1 to 7 days in response to ILTV infection.

## Results and Discussion

### Gene expression profile of lung cells infected by ILTV

**Figure 1:**
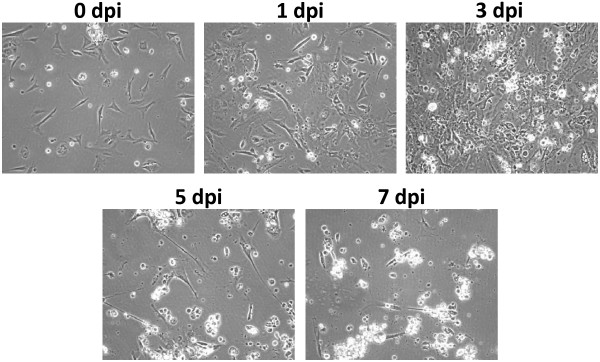
**ILTV infection in chicken embryonic lung cells**. The chicken embryo lung cells were infected with ILTV at a multiplicity of infection (MOI) of 0.1. The infected cells were visualized at 0, 1, 3, 5, and 7 dpi, respectively, using a phase contrast microscope at 200× magnification.

Primary chicken embryo lung cells at passage 1 were infected by the USDA reference strain of ILTV and cells were collected at 1, 3, 5, and 7 dpi. Cytopathic effects (CPE) were observed by 3 dpi, which became more severe by 5 dpi. Massive cell disruption was observed at 7 dpi (Figure 1). Total RNA was isolated from both controls and infected lung cells at each dpi time point and subjected to microarray analysis.

To control for dye bias effects, spike-in control mixtures were utilized by mixing with RNA samples according to the manufacturer's recommendations (See Methods). The spike-in RNA controls consisted of two sets of synthetic RNA mixtures derived from the Adenovirus E1A genes with different concentrations in each set [29]. The Agilent chicken 4 × 44 K oligo gene expression array contains 320 spike-in indicator spots to be hybridized with spike-in controls of both A mix, which was hybridized with Cy 3, and the B mix hybridized with Cy 5 on each array. These spike-in sets were mixed with either uninfected control or infected samples and co-hybridized to arrays. The ratio of signal intensities for all spike-in spots were calculated, evaluated, and revealed no significant dye effects on all array slides (data not shown) as reported previously [29]. All raw and normalized data were deposited in the Gene Expression Omnibus (GEO; accession number: GSE20630).

**Figure 2:**
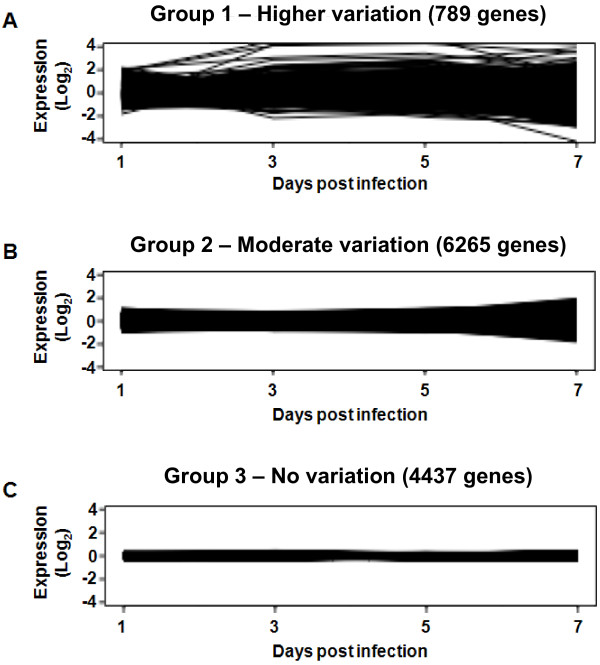
**Groups for sorting differentially expressed genes in the time course of ILTV infection**. The 11,491 genes showing a signal to noise ratio (SNR) > 3 were sorted into three groups based on alterations of fold changes at each dpi time point. The Y-axis represents log_2 _values of fold changes and the X-axis indicates dpi time points.

Normalized signal intensities were subjected to statistical analysis to find differentially expressed genes during ILTV infection in cultured embryonic lung cells. The 44 K array revealed 11,491 genes with significant signal intensities that were sorted by signal to noise ratio (SNR) >3, meaning that real (forward) signals of the samples were three times greater than background signals. In order to discover time course change in gene expression patterns, a model-based method [30] was used for clustering the gene expression profiles. A key drawback in heuristic clustering techniques is that it is difficult to determine the number of clusters *a priori*. The method enables the number of clusters to be determined by estimating the number of components in a multivariate normal mixture model from which the data are generated. The clustering analysis resulted in three gene groups (Figure 2). Group 1 included a total of 789 genes that showed significant differential expression in response to ILTV, Group 2 included 6,265 genes that displayed moderate alterations, and Group 3 included 4,437 genes that revealed no alterations during ILTV infection at four time points in chicken lung cells. Of the 789 genes in Group 1 exhibiting differential expression in response to ILTV (see Additional file 1), the top 10% (79 genes) were sorted by statistical analysis based on the highest value of standard deviations using the mean values of four different time points (Table 1). This approach highlights genes with more significant alterations in response to ILTV overtime. Out of the 789 genes, 390, 370, 320, and 422 genes were down-regulated, while 399, 419, 469, and 367 genes were up-regulated relative to uninfected cells at 1, 3, 5, and 7 dpi, respectively.

**Table 1:** Top 10% (79 out of 789 genes) of the most highly differentially expressed

Accession #	Gene Symbol	Fold Change			
		
		Day 1	Day 3	Day 5	Day 7
Y14971	CXC chemokine K60	3.7	17.3	19.7	11.3
X65459	FABP7	1.1	0.3	0.8	0.2
X16881	CDC2	1.9	0.4	0.7	0.4
X03509	CKB	0.9	4.7	5.5	2.0
X02009	LTF	0.7	2.7	3.6	3.2
U62026	CENPF	3.3	0.7	1.4	0.6
U12438	RFC2	1.9	0.8	1.0	0.3
U09350	VIP	2.0	0.2	0.3	0.2
M16199	IL8	3.7	22.4	26.8	18.5
CR733296	LIPG	1.0	0.7	0.6	0.1
CR523746	TMEM196	1.0	2.3	2.8	0.7
CR406543	SELO	1.0	0.9	1.1	4.1
CR406252	Prematurely terminated mRNA decay factor-like	1.2	1.1	1.4	4.7
CR391404	ITGA8	0.7	0.8	0.5	0.2
CR391234	LL	1.5	0.3	0.6	0.2
CR387914	CHAD	1.1	0.8	0.7	0.2
CR385491	IDl1	1.3	0.8	0.9	0.2
CR385166	MYCN	0.6	1.0	1.1	5.7
CR385124	DHCR7	1.3	0.9	1.0	0.3
CR382435	HDGFRP3	0.8	0.9	0.9	5.1
CR352395	OSTN	0.8	0.9	0.9	4.2
CO635775	HSP90AA1	0.8	0.5	0.5	2.6
CN218923.1	ARHGEF9	1.1	1.3	0.9	5.5
CF250950	ALDH1A3	0.6	2.1	2.3	3.6
CD763113	FDPS	1.1	0.6	0.7	0.2
BX936026	AURKA	2.4	0.5	0.7	1.0
BX935864	XBP1	0.9	0.8	0.7	3.4
BX935550	AKR1D1	0.6	2.9	2.7	1.2
BX935026	MAT1A	0.7	1.0	0.7	3.5
BX934121	TFPI2	0.9	1.5	2.2	5.5
BX932212	PTTG1	2.1	0.7	0.8	0.3
BX931971	SPON2	1.6	2.8	4.0	12.1
BX931663	ROPN1L	0.7	2.7	1.1	5.6
BU456021	SNAl1	1.9	1.0	0.7	0.4
BU409770	HMG_COA_S	2.5	1.4	1.2	0.3
BU200000	TNFAIP6	1.0	1.9	4.0	0.4
BU138507	CYP51	1.0	0.7	0.7	0.2
AJ851480	ACSL1	1.2	7.3	8.7	7.3
AJ721110	VNN2	0.5	3.3	6.5	2.5
AJ721107	SLA	0.8	1.1	1.4	5.0
AJ720861	LSS	1.3	1.1	1.3	0.2
AJ720657	DNAJB9	0.9	0.9	0.8	3.8
AJ720217	STARD4	1.8	0.9	1.0	0.3
AJ719858	ITFG1	1.3	1.0	1.2	4.8
AJ719718	SC4MOL	1.1	0.8	1.1	0.2
AJ719295	INSIG1	1.5	0.9	0.9	0.2
AJ443395	TRIP13	2.1	0.6	0.7	0.4
AJ393939	ITPR3	1.5	1.4	1.8	0.4
AJ309540	IL6	0.9	4.8	7.2	3.1
AJ004940	HSPA8	0.9	0.5	0.6	2.7
AF432506	FABP4	1.0	5.3	7.1	14.9
AF411083	SFTPA1	1.7	0.4	0.9	0.4
AF070478	MMP-13	0.7	3.4	2.9	0.7
AF062392	MMP27	0.8	4.1	6.6	3.5
AB031398	LEFTY2	1.2	1.7	1.8	5.8
BU306841	NSDHL	1.5	0.6	0.8	0.1
BU144940	ATF3	0.6	0.9	2.0	3.2
BU106686	MKI67	2.6	0.8	1.1	0.5
BU336892	HSPH1	1.0	0.5	0.5	3.2

CR385186	PREDICTED: similar to CUG2	2.2	0.5	0.8	0.4
CR387761	PREDICTED: similar to Gap junction alpha-7 protein	1.2	0.7	0.8	0.2
BU456843	PREDICTED: similar to Cancer susceptibility candidate 5	2.9	0.7	1.2	0.6
BU468099	PREDICTED: similar to Histone protein Hist2h3c1	2.5	0.9	0.9	0.4
BX950657	PREDICTED: chemokine (C-C motif) receptor-like 1 isoform 1	1.1	1.3	2.5	0.3
CR390562	PREDICTED: hypothetical protein	1.2	1.2	1.3	6.2

CR388632	Unknown	0.7	1.4	0.8	0.2
BU212825	Unknown	0.7	0.6	0.6	0.2
BU281664	Unknown	1.1	0.8	0.9	0.2
BU377399	Unknown	0.9	1.4	1.2	5.3
BU420694	Unknown	0.6	18.2	26.2	3.1
BU433279	Unknown	0.7	1.2	2.4	3.5
CR385201	Unknown	0.6	5.0	7.0	0.6
CR385678	Unknown	1.1	1.3	1.8	5.5
CR386845	Unknown	1.0	0.8	0.7	3.1
CR389767	Unknown	1.2	3.2	3.2	0.5
CR389813	Unknown	1.0	1.3	1.2	5.9
CR390519	Unknown	1.4	1.5	1.8	8.3
CR391100	Unknown	0.3	3.0	4.1	0.4
DR431104	Unknown	1.2	1.7	1.9	0.3

The highly differentially expressed genes were sorted. All genes were matched and verified with UniGene function of NCBI database.

### Quantitative reverse transcription-PCR (qRT-PCR)

To validate the microarray data, 20 genes were subjected to qRT-PCR using gene specific primer pairs and the same RNA samples as in the microarray analysis (Table 2). Results were analyzed by 2^-∆∆Ct ^method to determine relative levels of gene expression at each dpi time point compared to uninfected control [31]. There were no differences between microarray data and the qRT-PCR at any dpi time point (Table 3). However, it should be noted that fold change values for certain genes obtained by qRT-PCR analysis showed much greater expression levels than those observed in the microarray analysis. For example, the fold changes for the gene expression of matrix metalloproteinase (MMP) 27, interleukin (IL) 6, fatty acid binding protein (FABP) 4, IL8, and CXC chemokine K60 at 3- or 5 dpi showed much higher levels in qRT-PCR analysis compared to fold changes shown in microarray analysis (Table 3). Possibly, this qualitative difference between methodologies may be attributed to the upper detection limits of the fluorescent intensities for the array scanner. Based on quality control measures, such as the spike-in controls and the results of targeted qRT-PCR indicate that the microarray data sets for differential gene expression are valid to investigate genome-wide differential expression patterns for host responses during ILTV infection.

**Table 2:** Primers used for qRT-PCR

Accession #	Forward Primer	Gene Symbol
	Reverse Primer	
AJ711110	TGGTGGCTCGTTACCACAAG	VNN2
	TTCCCAAAGGGAGTCTCGAA	
AF070478	TCCCAAAACGCCAGAGAAAT	MMP-13
	TCGCCAGAAAAACCTGTCCT	
AF062392	CAGCCCCAGTGAATTTCCTC	MMP27
	GACGGTTGGCCTTTTACCTG	
X03509	CCAGGGGTATCTGGCACAAT	CKB
	TCATGTTGCCACCTTTCTGC	
AJ309540	CCTGTTCGCCTTTCAGACCT	IL6
	GCCAGGTGCTTTGTGCTGTA	
AF432506	CCTGTTCGCCTTTCAGACCT	FABP4
	GCCAGGTGCTTTGTGCTGTA	
X65459	GGACAGCCACAACTTTGACG	FABP7
	GCTGCTGATGATCACTGTGG	
AJ851480	TGATGCAAGCACACGACTTG	ACSL1
	ACCCACCAGGGTATTTGTCG	
AJ720861	AGGTTCACCCAGATCCCAGA	LSS
	CCACAGTCCCGTGTGCTAAA	
AJ719295	CTGTTTCCCGACGAGCTCAT	INSIG1
	GGTACAGCAGGCCAACAACA	
AJ719718	GGCAGTGAACGACAGCGTTA	SC4MOL
	TAAATGGCTGCTGCAGAGGA	
U09350	CGAAAGCAAATGGCTGTGAA	VIP
	TGCTTCACCTCGAAGTTTGG	
U62026	GAATGCTGGCACCAGGAAA	CENPF
	TCCGGAAAGGTTCCATCATC	
M16199	CGCTGGTAAAGATGGGGAAT	IL8
	CTTGGCGTCAGCTTCACATC	
X02009	GATAGCGGCTGTGTGTTTCG	LTF
	GAGGTCCCTGAGGTTGTTGC	
AJ004940	CTGAATTCAAGCGCAAGCAC	HSPA8
	TGACAGGGTACGCTTTGCAC	
AF411083	GTTGCTTTGCTAACGCCTTG	SFTPA1
	AGAGCTCCCAGACCAAGCAG	
X16881	TTCCACGGGGACTCAGAGAT	CDC2
	TGCAAGGATTCCACATCAGG	
U12438	GTCAGCAGGCTGGAGGTCTT	RFC2
	AGCAGAGGATGCTCCTCCTT	
Y14971	GGCTGTAGCTGCTGTCATGG	CXC chemokine K60
	TATGCACTGGCATCGGAGTT	
NM_204305	GGCACTGTCAAGGCTGAGAA	chGAPDH
	TGCATCTGCCCATTTGATGT	

The first column indicates the NCBI accession number for designated genes, and the second column shows sequences for the forward and reverse primers. The gene symbols are provided in the third column.

**Table 3:** Comparison of fold changes between microarray and qRT-PCR

Gene Accession #	Gene Symbol	1 Day		3 Day		5 Day		7 Day	
		
		Micro-array	RT-PCR	Micro-array	RT-PCR	Micro-array	RT-PCR	Micro-array	RT-PCR
AJ721110	VNN2	0.5	0.3	3.3	3.5	6.5	8.7	2.5	2.6
AF070478	MMP13	0.7	0.8	3.4	3.7	2.9	3.8	0.7	0.5
AF062392	MMP27	0.8	0.8	4.1	7.8	6.6	23.7	3.5	7.9
X03509	CKB	0.9	1.4	4.7	6.8	5.5	11.0	2.0	3.3
AJ309540	IL6	0.9	1.1	4.8	7.7	7.2	37.1	3.1	5.7
AF432506	FABP4	1.0	1.2	5.3	7.8	7.1	33.4	15.0	10.0
X65459	FABP7	1.1	1.5	0.3	0.8	0.8	1.1	0.2	0.3
AJ851480	ACSL1	1.2	0.9	7.3	6.0	8.7	8.0	7.3	7.6
AJ720861	LSS	1.3	1.1	1.1	0.7	1.3	1.1	0.2	0.1
AJ719295	Insulin induced gene 1	1.5	1.3	1.0	0.7	0.9	0.9	0.2	0.1
AJ719718	SC4MOL	1.1	1.7	0.8	0.9	1.1	1.3	0.2	0.2
U09350	VIP	2.0	2.4	0.2	0.1	0.3	0.2	0.2	0.2
U62026	CENPF	3.3	9.9	0.7	0.7	1.4	2.2	0.6	0.6
M16199	IL8	3.7	3.0	22.4	43.4	26.8	172.7	18.5	39.0
X02009	LTF	0.7	0.5	2.7	2.3	3.6	5.2	3.2	4.1
AJ004940	HSPA8	0.9	1.1	0.5	0.4	0.6	0.5	2.7	3.2
AF411083	SFTPA1	1.7	2.5	0.4	0.7	0.9	0.9	0.4	0.3
X16881	CDC2	1.9	2.2	0.4	0.3	0.7	0.6	0.4	0.4
U12438	RFC2	1.9	2.0	0.8	0.6	1.0	0.9	0.3	0.2
Y14971	CXC chemokine K60	3.7	7.9	17.3	60.3	19.7	206.4	11.3	38.2

The gene expression levels of 20 genes at four different time points in microarray analysis were confirmed by qRT-PCR. The expression levels were presented by fold changes values in microarray analysis, while, for qRT-PCR, the values were calculated by 2^-ΔΔCT ^method [31], which would be comparable to fold changes in the microarray. All values are mean values determined by the calculation from three replicating assays.

### Expression clustering

**Figure 3:**
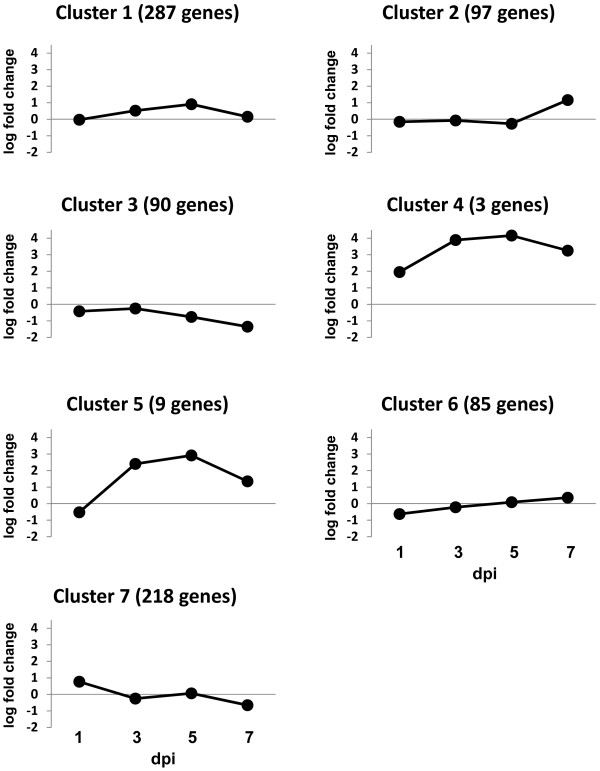
**Patterns of differential expression**. The mean value of each cluster was independently plotted in the graph. The closed circle displays dpi time points. The Y-axis indicates fold changes by log_2 _value.

The pattern of differential gene expression over time can provide insights into biologically functional relevance among genes. In the present study, a model-based clustering method [30] was used to cluster alteration patterns for the 789 differentially expressed genes in response to ILTV infection and revealed 7 gene clusters exhibiting distinct expression patterns (Figure 3 and Additional file 2). The 287 genes placed in cluster (C) 1 showed only nominal increases at 3 and 5 dpi followed by decreased expression levels at 7 dpi that were similar to those at the onset of the experiment. The C2 representing 97 genes exhibited a dramatic increase in gene expression only at 7 dpi, whereas the expression levels of the 90 genes in C3 progressively declined at 5 and 7 dpi. Three genes in C4 showed higher expression during early infection (1 dpi), sharp increases at 3 and 5 dpi, followed by a slight decline at 7 dpi. Expression patterns of 9 genes in C5 showed slightly lower expression at 1 dpi relative to the other time points, a dramatically increase at 3 and 5 dpi, followed by decreased expression at 7 dpi. The 85 genes in C6 showed lower expression at 1 dpi followed by a progressive increase during the later time points, which was opposite to 218 genes in C7 that showed higher expression at 1 dpi followed by decreased expression at 3, 5, and 7 dpi. GenBank accession numbers for genes in each cluster are shown in the Additional file 2.

Interestingly, the genes in C4 that exhibited the highest expression during ILTV infection include cytokines (IL8 and IL1β) and a chemokine (CXC-K60), while in the C5, IL6 was most highly expressed. From these findings, it is reasonable to hypothesize that expression of functionally relevant genes such as cytokines and chemokines released in response to an immune challenge may be regulated similarly during a specific immunological challenge.

### Functional Gene Ontology (GO)

**Figure 4:**
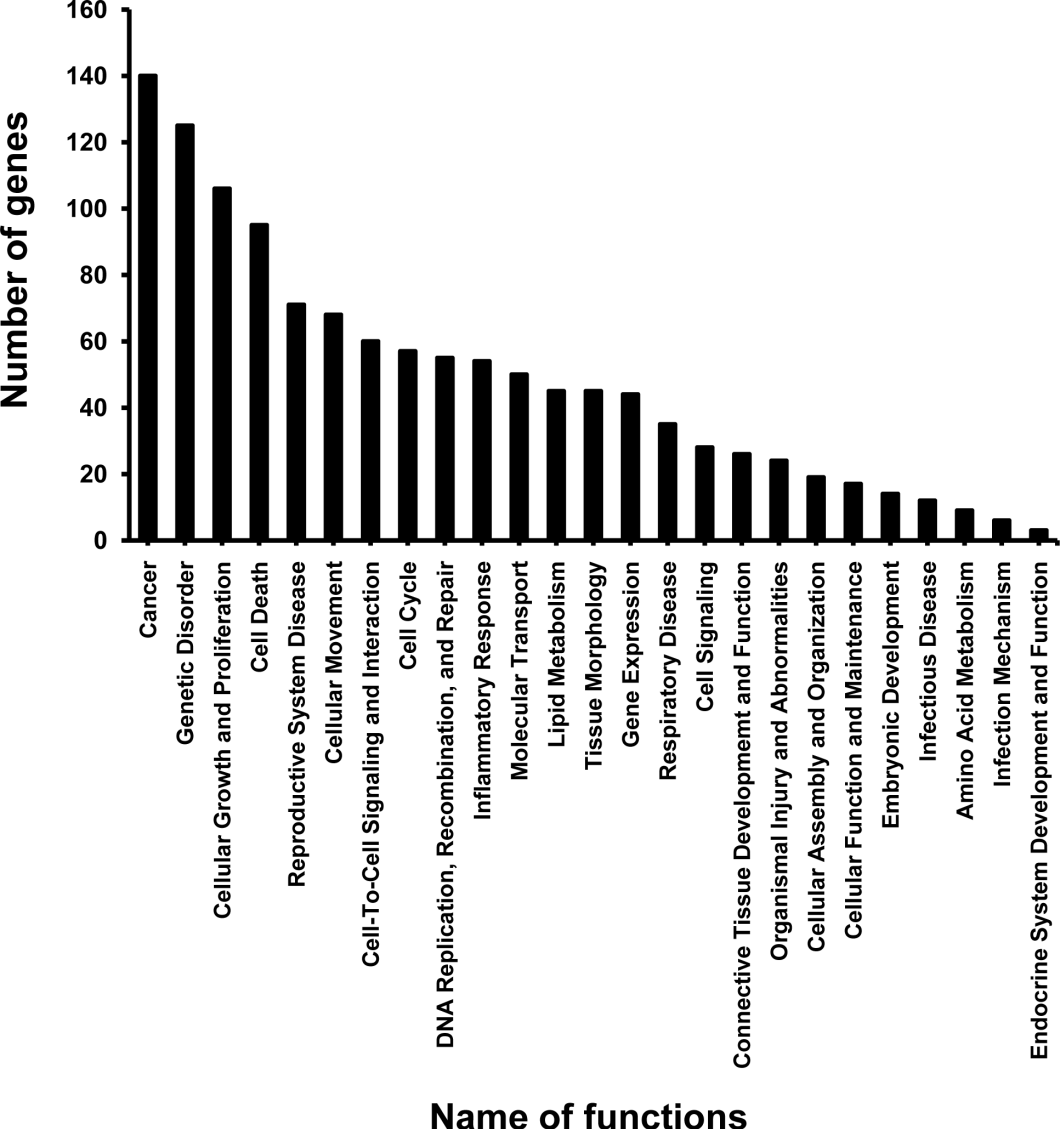
**Functional Gene Ontology (GO) for differentially expressed genes**. The 789 genes were categorized into functional groups by the IPA program. Bars represent the number of genes for each group. The Y-axis shows the total number of genes, and the X-axis indicates name of functional groups.

Recently, new bioinformatics tools have been developed to facilitate efficient analysis of biological functionalities for large numbers of differentially expressed genes obtained from microarray analysis. By using the IPA program (http://www.ingenuity.com/), bioinformatics aspects of differentially expressed genes during ILTV infection were analyzed for the relevance of gene functionalities and gene networks. While 789 differentially expressed genes were used as the input number of genes, only 275 have been characterized with specific cellular functions according to the IPA program. Results obtained with the IPA program in terms of numbers of genes, biological functions of genes and categories were independently confirmed with a second bioinformatics tool, DAVID (**D**atabase for **A**nnotation, **V**isualization and **I**ntegrated **D**iscovery) version 6.7 (http://david.abcc.ncifcrf.gov/) (data not shown). The group of 275 differentially expressed genes was placed into 65 functional groups (see Additional file 3) and the top 25 functional groups of genes are displayed in Figure 4. The main categories for gene functionalities include diseases and disorders, molecular and cellular functions, and physiological system development. Gene information was repeatedly used in multiple groups of functionalities due to the multi-functional characteristics for designated genes. It can be seen that the cancer related function contains the highest number (140) of genes, while 125 genes were involved in genetic disorders, and 54 genes were grouped as inflammatory responses.

### Gene network analysis

**Figure 5:**
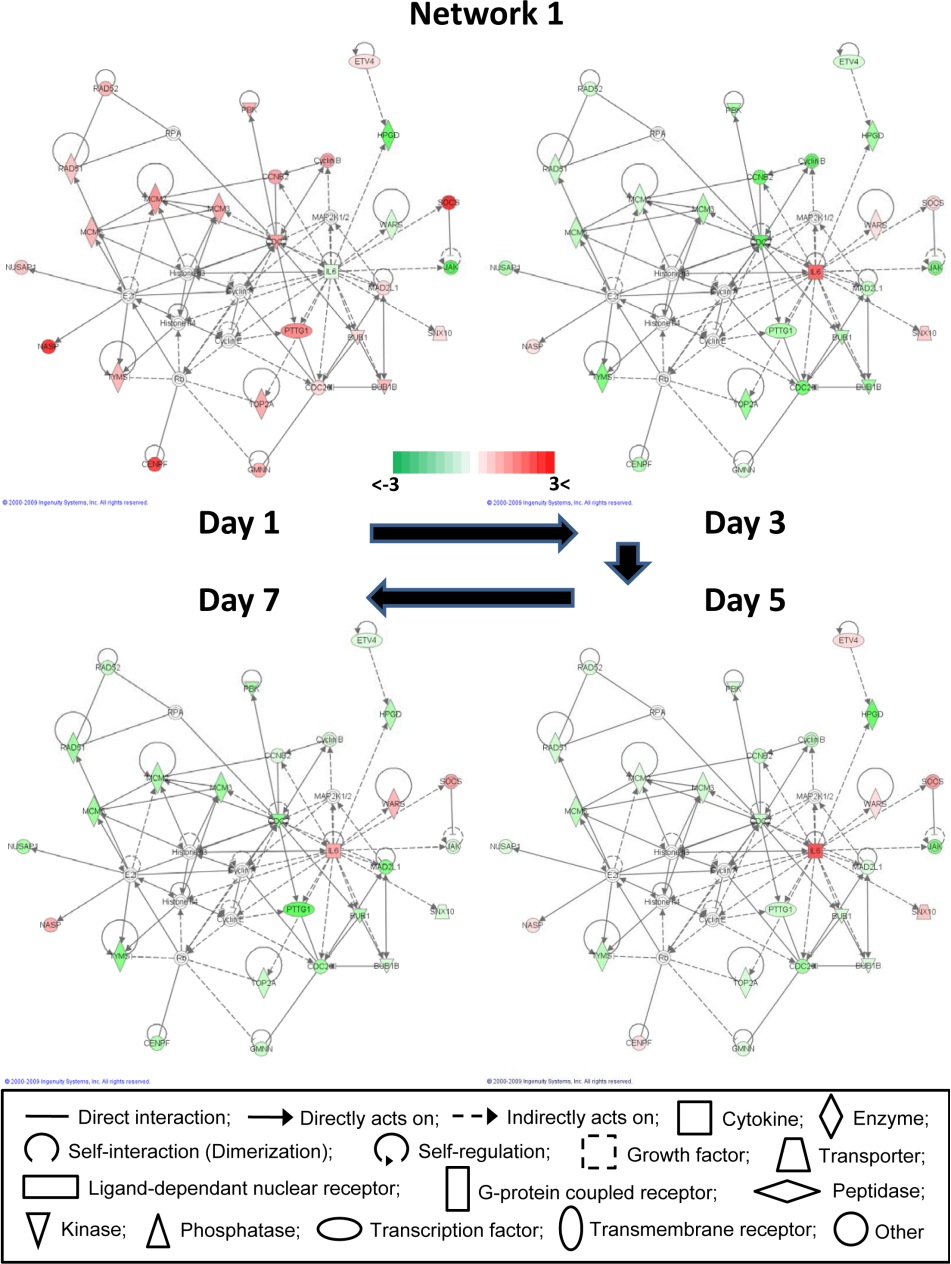
**Network #1 of gene network analysis**. Molecular interactions among important focus molecules are displayed at each dpi time points. Green represents down-regulation while red depicts up-regulation. White symbols depict neighboring genes. The intensity of color represents the average of log fold change in a given population. The numbers below the color change bar denote log_2 _values. Symbols for each molecule are present according to molecular functions and type of interactions.

Gene network analysis, which represents the intermolecular connections among interacting genes based on functional knowledge inputs, was performed on the differentially expressed genes using IPA program (see Additional file 4). In this way, 21 possible gene networks for all days post ILTV infection were generated based on differential gene expression. Of these 21 gene networks, only 6 gene networks were identical at all time points following ILTV infection. Possibly, the reason why only 6 of 21 networks were identical might be because of differences in the sets of focus molecules which are generated from p-values and fold change values of differential gene expression that are used in IPA algorithms. The dynamics of alterations in gene expression for a subset of genes during the time course of ILTV infection can provide insights into biologically interacting genes within a network that display functional similarities. The most interactive network (network #1) is presented in Figure 5 whereas the other networks are shown in Additional file 5.

Network #1 is closely associated with a signaling pathway of IL6, which is a cytokine known to be involved in cell proliferation and inflammatory responses [32]. The top functions related with genes in network #1 involve cancer, gastrointestinal disease, and the cell cycle. Interestingly, expression of certain genes in network #1 such as CDC20 (cell division cycle 20 homolog), PTTG1 (pituitary tumor transforming 1), CDC2, and Cyclin B, which are associated with cellular proliferation in cell cycle progression, appeared to be inversely related to IL6 expression. The dynamics of alterations in gene expression over time during ILTV infection suggest that ILTV infection elevates IL6 expression followed by the inhibition of cellular proliferation. In contrast, expression patterns of HPGD (hydroxyprostaglandin dehydrogenase 15-NAD), SOCS (suppressors of cytokine signaling), JAK (Janus kinase 1), and NASP (nuclear autoantigenic sperm protein) were independent of the IL6 expression pattern (Figure 5A, B and Additional file 5A). JAK is known to enhance cellular proliferation through the signal transducer and activator of transcription (STAT) pathway that can be suppressed by IL6 signaling [33]. The consistent downregulation of JAK supports a role of JAK in the repression of cellular proliferation by ILTV infection. The top functions of genes in network #2 are involved with cellular compromise, connective tissue disorders, and post-translational modifications. Several heat shock proteins (HSP) were also focused in this network (see Additional file 5B). Heat shock proteins, especially the HSP70 family that serve as molecular chaperones, are known to interact with viral early immediate genes in HSV-1 genomic DNA replication [34]. Interestingly, since the expression of several HSPs in network #2 were downregulated, it is reasonable to hypothesize that the lower HSP through 5 dpi may lead to production of erroneous virion structures of ILTV that in turn results in low ILTV titers in tissue culture, which has been reported to barely exceed one infectious unit per cell [1,35].

Network #3 contains genes for growth factors and matrix metalloproteinases (MMPs), and genes have top functions associated with endocrine system function and development, carbohydrate metabolism, and digestive system function and development (see Additional file 5C). Expression for growth factors and MMPs increased on 3 dpi and remain elevated through 7 dpi. This observation is in agreement with other reports that infection with herpesviruses, such as HSV and HCMV, lead to an increase in growth factor expression and MMPs for extracellular remodeling, tissue invasion and angiogenesis [36-38]. Networks #4 and #5 contain cytokine genes (IFNβ and IL1β), chemokine genes (CCL20 and CCL4), and genes of the NF-kB families (NF-kB and NFIB) with top functions that are involved in organism injury and abnormalities, antigen presentation, cell mediated immune responses, lipid metabolism, small molecule biochemistry, and molecular transport (see Additional file 5D and 5E). Finally, genes in network #6 contain IL1, NF-kB, and ID1 that function in cardiac inflammation, cardiovascular disease and in the inflammatory response (see Additional file 5F). Interacting molecules found in networks #4, 5, and 6 are mostly focused on the host immune responses against pathogenic infections.

The network analysis suggests that a large number of biological pathways, regulated by various sets of genes, closely interact with each other in host responsiveness during ILTV infection. More detailed interactions among genes showing altered expression levels in each network are currently under investigation to identify host-response mechanisms that may occur in conjunction with general immunological reactions during ILTV infections.

**Figure 6:**
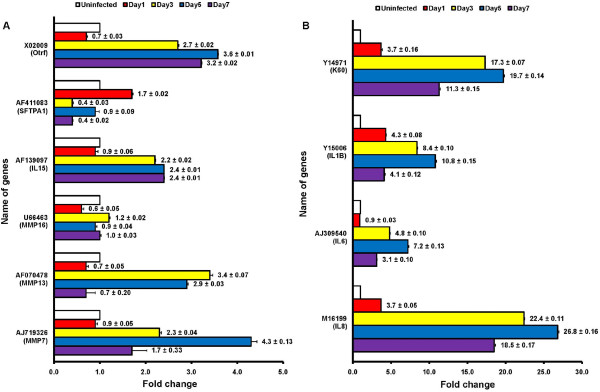
**Genes of interest in the time course of ILTV infection**. The expression levels for genes of interest at each time point displayed by the bar graphs; Genes showing lower range of alteration levels are displayed in (A), whereas genes ranging higher alteration levels displayed in (B). The open, red, yellow, blue, and purple bars reflect 0, 1, 3, 5, and 7 dpi, respectively. Graphs show the mean ± SE.

The fold changes in gene expression of key molecules associated with cellular immune response, cell signaling, MMP molecules, cytokines, chemokines, and cell proliferation were plotted individually (Figure 6). This was done since the molecules may help in clarifying the interaction of host lung cells with ILTV. Four matrix metalloproteinases (MMPs) including MMP 7, 13, 16, and 27 were differentially expressed during ILTV infections. For example, MMP 1, 2, and 9 were shown to function in cell invasions of primary human endothelial cell in Kaposi's sarcoma-associated herpesvirus (KSHV) pathogenesis [39]. Similarly the viral oncoprotein meq in MDV is known to activate MMP3 transcription [40]. Furthermore, the balance between MMP9 and tissue inhibitor of metalloproteinases 1 (TIMP1) was altered in human macrophages in HCMV infection, such that MMP9 activity declined in response to HCMV infection. Moreover, it was shown that HCMV infection may affect atherogenesis in mice through the control of MMP9 expression [38]. Taken together, these results suggest that MMPs generally play a role in herpesvirus pathogenesis, but different isoforms of MMPs may be capable of responding to specific herpesviruses. Furthermore, in the present study with chicken lung cells, expression of surfactant protein A (SFTPA-1; GenBank accession - AF411083; Table 1 and 3) was downregulated by ILTV infection. Since the SFTPA-1 is a transcriptional indicator of EGFR (epidermal growth hormone receptor) signaling pathway [41], the reduction in SFTPA-1 expression suggests that the EGFR signaling pathway is suppressed by ILTV infection. This conclusion is consistent with the reported downregulation of EGFR functions in HCMV infected human lung [41] and foreskin fibroblastic cells [42]. Additionally, our findings are similar to a report in which the mRNA expression of SFTPA-1 declined after inoculation of influenza A virus H9N2 into chicken lung cells [43].

Another molecule, ovotransferrin (Otrf; GenBank accession-X02009; Table 1 and 3) has both iron transport- and antibacterial-activities. In the present study, the expression level of Otrf increased in ILTV infected cells (Figure 6A). The finding is qualitatively similar to a possible role of Otrf in MDV infection that might protect the spread of MDV in chicken embryonic fibroblast cells [44]. Otrf also was found to accelerate the expression of immune response gene such as IFN-γ against MDV infection [45]. Furthermore, lactoferrin, which is a homologous form of transferrin in mammals, showed antiviral activity against canine herpesvirus [46]. It has been hypothesized that the anti-viral activity of Otrf may be similar to the anti-HSV capability of mammalian transferrins [47]. IL6 plays a role in both pro-inflammatory and anti-inflammatory responses [32]. The elevation of IL6 expression during virus infection has been well-characterized as one of the immune response activities in the pathogenesis of various viruses, such as Dengue virus in human [48], or HSV-1 in mice [49]. Also, the expression of IL6 is increased by KHSV-encoding miRNA [50]. Similarly, expression of IL8 and IL1β were upregulated in ILTV infected cells and also in MDV infected chicken lung [51]. These observations are consistent with previous findings of NF-kB activation via IL8 signaling pathway by human herpesvirus (HHV)-8 infection [52] and HHV-6 infection in peripheral blood mononuclear cell cultures [53], respectively.

In addition to the well-characterized host-virus interactions, a variety of unique responses detected with the microarray analysis of ILTV infection in the present study. For instance, the expression level of vasoactive intestinal peptide (VIP; GenBank accession number-U09350: Table 1 and 3) decreased dramatically in ILTV infection, but the functional consequences were not determined. Likewise, genes related to various metabolic enzyme functions such as acyl-CoA synthetase long chain-1 (ACSL1; GenBank Accession number-AJ851480; Table 1 and 3) were differentially expressed in ILTV infected cells, but the precise mechanisms in the host response have not been verified. Therefore, further investigations are being performed to identify unique and more deeply involved interactions between host lung cells and ILTV.

## Conclusions

In this study, we have demonstrated changes in global gene expression in response to ILTV infection in chicken embryo lung cells using microarray analysis. A total of 789 differentially expressed genes were involved in a variety of molecular and cellular defense mechanisms of immune response, cell cycle regulation, cellular metabolism, and matrix metalloprotenases. Moreover, the bioinformatics studies, such as gene ontology and gene network analysis, using knowledge based bioinformatics tools (Ingenuity Pathway Analysis and DAVID) revealed biological functionalities and intermolecular connections among interacting genes associated with differentially expressed genes. Our study provides epigenetic insights into the pathogenesis of ILTV infection in chicken lung cells.

## Methods

### Cell culture and ILTV infection

Cell culture reagents were purchased from Invitrogen Life Technologies (Carlsbad, CA, USA). Chicken embryo lungs were isolated from 19 day old specific-pathogen free (SPF) chicken embryos (Charles River Laboratories, North Franklin, CT, USA). Lung tissues were homogenized and incubated in a 0.125% trypsin solution for 30 min at room temperature (25°C). Cells dissociated from lung tissues were suspended in a 1:1 ratio of mammary epithelial growth media (MEGM; Lonza, Rockland, ME, USA) and Dulbecco's Modified Eagle's Medium (DMEM, 0.45% glucose) plus 2% fetal bovine serum (FBS), 100 units/ml penicillin, 100 μg/ml streptomycin, and 2 mM L-glutamine in 10 cm tissue culture dishes (Sarstedt Inc., Newton, NC, USA) pretreated with 0.5% gelatin in PBS to improve cell adhesion. Cultured cells were grown at 39°C containing 5% CO_2 _until cells reached confluent monolayers (2 to 4 days). The USDA reference strain of ILTV (National Veterinary Services Laboratories, Ames, IA, USA) was used to infect the chicken embryonic lung cells at a multiplicity of infection (m.o.i.) of 0.1. Infected cells were incubated at 37°C for 1 hr with rocking gently every 15 min. After the incubation, 10 ml of media, 1:1 MEGM/DMEM, were added to each culture dish, and the cells were incubated at 37°C in 5% CO_2 _for up to 7 days. This research was performed under the permitted protocol approved by both the Institutional Biosafety Committee (IBC; permit number: 10007) of University of Arkansas and the Animal and Plant Health Inspection Service (APHIS; permit number: 102743) of United States Department of Agriculture (USDA).

### Total RNA extraction

Total RNA was extracted from uninfected- or ILTV infected chicken embryonic lung cells at 1, 3, 5, and 7 dpi using TRIzol reagent (Invitrogen Life Technologies, Carlsbad, CA, USA) following the manufacturer's instructions. Total RNA was treated with DNase I (New England BioLabs Inc., Ipswich, MA, USA), and RNA was re-purified by the TRIzol reagent. The quality of RNA was checked by fractionation on an agarose gel (data not shown).

### Probe labeling and microarray hybridization

A two color labeling microarray system was used to compare uninfected- and ILTV infected embryonic lung cells at 1, 3, 5, and 7 dpi. Fluorescently labeled complementary RNA (cRNA) probes were generated by using the Two Color Microarray Quick Labeling kit (Agilent Technologies, Palo Alto, CA, USA) and following the manufacturer's instructions. RNA spike-in controls were used to adjust possible dye effects following manufacturer's instructions. The Spike-in controls represent two sets of ten synthesized RNA mixtures derived from the Adenovirus E1A transcriptome with different concentrations in each set [29]. These spike-in sets were mixed with either uninfected control or infected samples and co-hybridized to arrays. Briefly, 2 μg of total RNA were mixed with Spike-ins and converted to cDNA using reverse transcriptase and oligo dT primers in which T7 promoter sequences were added. T7 RNA polymerase was used for the synthesis and labeling of cRNA with either Cy3 dye for the uninfected control or Cy5 dye for the ILTV infected samples. The fluorescently labeled cRNA probes were purified using the Qiagen RNeasy Mini Kit (Qiagen Inc., Valencia, CA, USA), and the concentration, fluorescent intensities, and quality of labeled cRNA probes were determined using a Nano-drop spectrophotometer (Thermo Scientific, Wilmington, DE, USA). An equal amount (825 ng) of Cy3 and Cy5 labeled cRNA probes were hybridized on a 4 × 44 K Agilent custom chicken oligo microarray (array ID: 017698). The hybridized slides were washed using a commercial kit package (Agilent Technologies, Palo Alto, CA, USA) and then scanned using a Genepix 4000B scanner (Molecular Devices Corporation, Sunnyvale, CA, USA) with the tolerance of saturation setting of 0.005%. Three biological replicates were conducted.

### Microarray data collection and analysis

Background-corrected red and green intensities for each spot were used in the subsequent analysis. Global normalization based on local polynomial regression (loess) was applied to the intensities to remove effects that were due to undesirable systematic variations in microarray experiments rather than biological differences. The average values of the resulting normalized expression values in replicate hybridization sets were considered in the subsequent analysis. In order to identify a given set of genes that exhibited major alterations over time, a model-based clustering method [30] was employed, and the genes in the cluster were considered as differentially expressed over the time period. All analytic techniques were implemented in R (http://www.R-project.org).

### Quantitative reverse transcription-polymerase chain reaction (qRT-PCR)

Reverse transcription was performed with 3 μg of total RNA using Superscript II reverse transcriptase (Invitrogen Life Technologies, Carlsbad, CA, USA) with oligo dT_12-18 _primers (Invitrogen Life Technologies, Carlsbad, CA, USA) following the manufacturer's instructions. The reverse-transcribed cDNA were diluted by 1:10 ratio and a portion (1 μl) of each product was subjected to qPCR under the following conditions: 40 cycles of 95°C for 30 s, gene-specific annealing temperature for 62°C for 1 min, extension for 30 s at 72°C, and a final extension at 72°C for 10 min. A non-template control and endogenous control (chicken GAPDH) were used for the relative quantification. The differential expression levels for the ILTV infected group were compared by the 2^-ΔΔCT ^method against the uninfected controls [31]. Primers for qRT-PCR were designed using Primer3 software (http://frodo.wi.mit.edu/cgi-bin/primer3/primer3.cgi) with these parameters: amplicon length, 95-100 bp; primer length, 18-27 nucleotides; primer melting temperature, 60-64°C; primer and amplicon GC content, 20-80%; difference in melting temperature between forward and reverse primers, 1-2°C. Primers were synthesized by Integrated DNA Technologies (Coralville, IA, USA). Primer information is listed in Table 2.

### Bioinformatics

Functional interpretation of differentially expressed genes was analyzed in the context of gene ontology and molecular networks using the Ingenuity Pathways Analysis (IPA) 6.5 software (Ingenuity Systems^®^; http://www.ingenuity.com). The differentially expressed genes were categorized, compared to genetic categories in the IPA database, and ranked according to p-values [54]. The IPA analysis determined the subcategories within each category which is supplied with an appropriate p-value and the number of genes identified. Since the size of the created network could potentially be enormous, the IPA software limited the number of molecules in the network to 35, leaving only the most important ones based on the number of connections for each focus gene (focus genes = a subset of uploaded significant genes having direct interactions with other genes in the database) to other significant genes [55].

## Authors' contributions

JYL and BWK designed the experiments, performed the experiments, analyzed the data, and wrote the manuscript. JJS contributed in statistical analysis of microarray data, and AW participated in virus preparation and helped analysis of the qRT-PCR data. XL and HZ contributed the analysis of microarray data. WB contributed the bioinformatics analysis and manuscript editing. All authors read and approved the final manuscript.

## Supplementary Material

Additional file 1 **List of 789 highly variable genes expressed differentially**. The values at each time point indicate fold changes. The black letters indicate characterized genes, the orange letters are predicted genes, and the green letters denote uncharacterized genes. The accession numbers and descriptions were derived from Agilent gene list and GenBank database. Click here for file

Additional file 2 **GenBank accession numbers of 7 clusters for expression patterns**. Click here for file

Additional file 3 **Gene Ontology generated by IPA**. The 789 differentially expressed genes were divided into 65 groups based on their functions. Click here for file

Additional file 4 **Gene lists of each network**. Gene symbols and GenBank accession numbers were displayed for the illustrations of network analysis. Only focus molecules, which were elected as differentially expressed genes from microarray analysis, are marked as bold and GenBank accession numbers are provided. Accession numbers for reference molecules were not included in the table. Click here for file

Additional file 5 **Six gene networks**. (A) network #1 (B) network #2 (C) network #3 (D) network #4 (E) network #5 and (F) network #6 are displayed. Enlarged images are followed by small alphabetical orders (a - d) to indicate dpi time points. Colored shapes indicate focus molecules, which were identified as differentially expressed genes by microarray analysis, while clear shapes indicate reference molecules. The green represents down-regulation and the red represents up-regulation. Degree of color intensities indicates levels of fold changes. Click here for file
