# Supplementary material for: Transcriptional profiling of host gene expression in chicken embryo lung cells infected with laryngotracheitis virus
Source: BMC Genomics. 2010 Jul 21;11:445. doi: 10.1186/1471-2164-11-445 (PMC3091642; doi:10.1186/1471-2164-11-445)
Supplement: Additional file 1 — List of 789 highly variable genes expressed differentially. The values at each time point indicate fold changes. The black letters indicate characterized genes, the orange letters are predicted genes, and the green letters denote uncharacterized genes. The accession numbers and descriptions were derived from Agilent gene list and GenBank database. [file 1471-2164-11-445-S1.PDF]

Accession #	Day 1	Day 3	Day 5	Day 7	Description
AB007445	1.7	0.6	0.8	0.7	Topoisomerase (DNA) II alpha 170kDa (TOP2A)
AB031025	1.3	1.6	2.7	1.4	Platelet-derived growth factor beta polypeptide (simian sarcoma viral (v-sis) oncogene homolog) (PDGFB)
AB031398	1.2	1.7	1.8	5.8	Left-right determination factor 2 (LEFTY2)
AB045597	0.8	1.1	1.4	2.7	Peroxisome proliferator-activated receptor gamma (PPARG)
AB055783	1.3	0.6	0.9	0.6	Centromere protein H (CENPH)
AB100407	1.4	1.6	2.1	2.1	Phosphatase and actin regulator 1 (PHACTR1)
AB101005	1.1	3.1	2.6	1.3	Chemokine (C-C motif) ligand 20 (CCL20)
AB105812	0.9	1.4	1.8	1.1	GTP binding protein overexpressed in skeletal muscle (GEM)
AB154518	0.8	0.8	0.9	2.8	Heat shock protein 25 (HSP25)
AF000241	1.8	1.3	1.6	0.9	Nuclear factor of kappa light polypeptide gene enhancer in B-cells 1 (NFKB1)
AF020315	0.7	1.3	1.3	2.5	Purinergic receptor P2X, ligand-gated ion channel, 4 (P2RX4)
AF031168	0.9	1.7	2.6	0.6	Wingless-type MMTV integration site family, member 9A (WNT9A)
AF051399	1.1	2.0	2.1	1.3	Fibulin 1 (FBLN1)
AF053401	3.1	1.3	2.3	1.3	Serpin peptidase inhibitor, clade B (ovalbumin), member 10 (SERPINB10)
AF062392	0.8	4.1	6.6	3.5	Matrix metallopeptidase 27 (MMP27)
AF068831	2.5	0.9	1.1	0.6	Inhibitor of DNA binding 2, dominant negative helix-loop-helix protein (ID2)
AF070478	0.7	3.4	2.9	0.7	Matrix metalloproteinase-13 (MMP-13)
AF075708	1.1	0.8	1.5	0.9	Ets variant gene 4 (E1A enhancer binding protein, E1AF) (ETV4)
AF096264	0.5	0.5	0.5	0.8	Janus kinase 1 (a protein tyrosine kinase) (JAK1)
AF098515	0.5	0.9	1.2	0.6	Ras homolog gene family, member B (RHOB)
AF119370	0.7	1.1	1.4	0.8	Clusterin (CLU)
AF125575	0.5	0.6	0.5	0.2	Deiodinase, iodothyronine, type II (DIO2)
AF131224	0.8	0.5	0.7	0.9	Heparin-binding EGF-like growth factor (HBEGF)
AF139097	0.9	2.2	2.4	2.4	Interleukin 15 (IL15)
AF153205	1.5	2.3	3.3	1.6	CD44 molecule (Indian blood group) (CD44)
AF239837	0.7	0.8	0.7	0.3	Nidogen 1 (NID1)
AF261079	1.0	1.2	2.4	0.7	Cbp/p300-interacting transactivator, with Glu/Asp-rich carboxy-terminal domain, 4 (CITED4)
AF285876	0.9	2.5	2.1	2.7	Chemokine (C-X-C motif) ligand 14 (CXCL14)
AF289218.1	0.9	1.5	1.0	1.0	Amyloid beta (A4) precursor protein (APP)
AF308592	1.8	1.0	1.2	2.7	Nucleolar protein family A, member 1 (H/ACA small nucleolar RNPs) (NOLA1)
AF327880	0.8	1.2	1.5	1.3	ENS-2 (ens-2)
AF411083	1.7	0.4	0.9	0.4	Surfactant, pulmonary-associated protein A1 (SFTPA1)
AF432506	1.0	5.3	7.1	14.9	Fatty acid binding protein 4, adipocyte (FABP4)
AF459286	2.2	0.9	1.2	1.0	Telomeric repeat binding factor (NIMA-interacting) 1 (TERF1)
AF472618	0.9	1.1	1.8	1.1	Endothelin receptor type A (EDNRA)
AF498103	1.7	0.8	0.9	3.3	LZ1 (LOC395251)
AF505881	0.5	1.0	0.9	0.4	Class II bHLH protein scleraxis (SCX)
AF534111	3.5	1.7	3.2	2.7	UDP-glucose pyrophosphorylase 2 (UGP2)
AF537108	0.8	1.4	1.4	0.7	Sidekick homolog 2 (SDK2)
AJ004940	0.9	0.5	0.6	2.7	Heat shock 70kDa protein 8 (HSPA8)
AJ307060	0.4	0.5	0.7	0.4	Retinoic acid receptor responder (tazarotene induced) 1 (RARRES1)
AJ309540	0.9	4.8	7.2	3.1	Interleukin 6 (interferon, beta 2) (IL6)
AJ393786	0.7	1.1	1.3	1.7	Regulator of G-protein signalling 1 (RGS1)

AJ393939	1.5	1.4	1.8	0.4	Inositol 1,4,5-triphosphate receptor, type 3 (ITPR3)
AJ442179	0.7	0.5	0.7	0.7	PREDICTED: similar to Protein phosphatase 1G (formerly 2C), magnesium-dependent, gamma
AJ443395	2.1	0.6	0.7	0.4	Thyroid hormone receptor interactor 13 (TRIP13)
AJ450520.1	1.0	1.9	2.0	1.0	Echinoderm microtubule associated protein like 4 (EML4)
AJ585767	0.8	1.0	1.6	0.8	ST3 beta-galactoside alpha-2,3-sialyltransferase 6 (ST3GAL6)
AJ719282	2.2	1.6	1.8	2.4	Spindle assembly 6 homolog (C. elegans) (SASS6)
AJ719295	1.5	0.9	0.9	0.2	Insulin induced gene 1 (RCJMB04_1d1)
AJ719296	1.4	1.5	1.2	2.9	Epidermal growth factor receptor pathway substrate 15 (RCJMB04_1d5)
AJ719321	0.7	1.4	1.3	1.8	Protein-L-isoaspartate (D-aspartate) O-methyltransferase domain containing 1 (PCMTD1)
AJ719326	0.9	2.3	4.3	1.7	Matrix metallopeptidase 7 (matrilysin, uterine) (MMP7)
AJ719335	1.1	0.6	0.8	2.0	Solute carrier organic anion transporter family, member 4A1 (SLCO4A1)
AJ719339	3.7	1.2	1.4	2.8	Nuclear autoantigenic sperm protein (histone-binding) (NASP)
AJ719348	2.5	0.8	1.1	1.0	Thymopoietin (TMPO)
AJ719352	1.8	0.6	0.8	0.5	Minichromosome maintenance complex component 3 (MCM3)
AJ719362	1.5	1.3	2.3	3.5	Riboflavin kinase (RCJMB04_1k1)
AJ719387	3.3	0.9	1.5	0.8	KIAA1524 (KIAA1524)
AJ719438	0.6	0.5	0.8	0.6	RAT Selenoprotein P precursor (SeP)
AJ719443	1.6	0.8	1.1	0.9	Heterogeneous nuclear ribonucleoprotein A2/B1 (RCJMB04_2g17)
AJ719454	1.2	0.7	0.7	1.6	Nucleolar protein 5A (56kDa with KKE/D repeat) (NOL5A)
AJ719475	1.8	0.6	0.9	0.5	Karyopherin alpha 2 (RAG cohort 1, importin alpha 1) (KPNA2)
AJ719507	1.2	0.7	0.9	0.3	Structural maintenance of chromosomes 4 (SMC4)
AJ719535	2.2	1.6	2.0	2.5	E2F transcription factor 6 (RCJMB04_3c14)
AJ719555	0.8	1.1	1.5	1.1	Neutrophil cytosolic factor 1 (RCJMB04_3f13)
AJ719593	2.0	0.8	0.9	0.6	Minichromosome maintenance complex component 2 (MCM2)
AJ719627	1.9	0.6	0.8	0.7	G-2 and S-phase expressed 1 (RCJMB04_4l2)
AJ719676	1.3	0.7	0.8	0.6	BUB1 budding uninhibited by benzimidazoles 1 homolog (yeast) (BUB1)
AJ719718	1.1	0.8	1.1	0.2	Sterol-C4-methyl oxidase-like (SC4MOL)
AJ719751	1.1	0.7	0.5	0.8	Pre-B-cell leukemia homeobox 3 (RCJMB04_6b10)
AJ719817	1.4	0.7	0.9	1.6	SWI/SNF related, matrix associated, actin dependent regulator of chromatin, subfamily a, member 5 (SMARCA5)
AJ719818	1.7	1.1	1.5	0.8	Serpin peptidase inhibitor, clade B (ovalbumin), member 6 (SERPINB6)
AJ719854	1.8	0.9	1.2	0.8	Heterogeneous nuclear ribonucleoprotein M (RCJMB04_7e17)
AJ719858	1.3	1.0	1.2	4.8	Integrin alpha FG-GAP repeat containing 1 (ITFG1)
AJ719869	1.5	1.8	2.7	3.2	CD47 molecule (CD47)
AJ719902	0.9	0.7	1.1	1.3	Epidermal retinal dehydrogenase 2 (RCJMB04_8a2)
AJ719903	1.1	0.8	1.4	1.7	Pleckstrin homology domain containing, family A (phosphoinositide binding specific) member 3 (PLEKHA3)
AJ719947	0.9	1.6	2.0	1.5	CD82 molecule (CD82)
AJ719973	1.7	1.2	1.3	0.6	Farnesyl-diphosphate farnesyltransferase 1 (FDFT1)
AJ719984	1.7	0.8	1.0	0.9	Centromere protein L (CENPL)
AJ720011	1.7	0.8	0.9	1.0	PREDICTED: similar to family with sequence similarity 29, member A (RCJMB04_9g9)
AJ720016	1.7	0.8	0.8	0.8	Minichromosome maintenance complex component 6 (MCM6)
AJ720017	1.1	0.9	1.5	0.5	Acyl-CoA synthetase bubblegum family member 2 (RCJMB04_9i11)
AJ720018	1.0	1.9	2.4	1.6	Phosphodiesterase 4B, cAMP-specific (phosphodiesterase E4 dunce homolog, Drosophila) (PDE4B)
AJ720030	1.4	0.9	1.2	0.3	Squalene epoxidase (SQLE)
AJ720043	1.1	0.5	0.9	0.6	Small nuclear ribonucleoprotein polypeptide A' (SNRPA1)

AJ720066	1.6	1.3	1.6	0.7	Adaptor-related protein complex 1, sigma 2 subunit (AP1S2)
AJ720074	1.6	0.7	0.7	0.5	Minichromosome maintenance complex component 5 (MCM5)
AJ720138	1.7	0.9	1.0	0.7	Geminin, DNA replication inhibitor (GMNN)
AJ720179	0.7	0.7	1.0	2.2	Methylenetetrahydrofolate dehydrogenase (NADP+ dependent) 2, methenyltetrahydrofolate cyclohydrolase (RCJMB04_12b8)
AJ720196	1.7	1.1	1.1	3.5	Paraspeckle component 1 (RCJMB04_12f9)
AJ720217	1.8	0.9	1.0	0.3	StAR-related lipid transfer (START) domain containing 4 (STARD4)
AJ720298	0.6	0.7	0.8	1.6	Thioredoxin 2 (TXN2)
AJ720303	1.7	0.9	1.2	3.0	6-phosphogluconolactonase (RCJMB04_14h20)
AJ720331	1.1	0.6	0.6	1.6	DnaJ (Hsp40) homolog, subfamily A, member 1 (RCJMB04_15h2)
AJ720339	1.5	0.7	0.9	0.5	Nucleolar and spindle associated protein 1 (NUSAP1)
AJ720344	0.9	1.1	0.8	2.9	Solute carrier family 16, member 9 (monocarboxylic acid transporter 9) (RCJMB04_15m4)
AJ720351	0.6	0.7	0.8	0.9	Palmitoyl-protein thioesterase 1 (ceroid-lipofuscinosis, neuronal 1, infantile) (PPT1)
AJ720366	1.7	0.7	0.9	0.4	ELOVL family member 6, elongation of long chain fatty acids (FEN1/Elo2, SUR4/Elo3-like, yeast) (RCJMB04_16d24)
AJ720410	1.8	0.8	0.8	0.4	DCMP deaminase (DCTD)
AJ720414	0.7	0.9	0.9	0.3	Neuroepithelial cell transforming gene 1 (NET1)
AJ720428	0.9	1.4	1.9	1.3	NSFL1 (p97) cofactor (p47) (RCJMB04_13o20)
AJ720471	1.1	0.8	1.0	3.2	Basic transcription factor 3-like 4 (BTF3L4)
AJ720504	1.2	1.5	2.7	1.4	Toll-like receptor 7 (TLR7)
AJ720523	0.7	0.9	0.6	0.9	Phosphatidylethanolamine N-methyltransferase (PEMT)
AJ720555	2.4	1.1	1.0	3.1	Neutrophil cytosolic factor 4, 40kDa (NCF4)
AJ720586	0.4	0.7	0.8	0.5	PREDICTED: similar to guanine nucleotide-binding regulatory protein gamma-3 subunit
AJ720638	1.3	1.1	1.8	0.9	C-terminal binding protein 1 (CTBP1)
AJ720657	0.9	0.9	0.8	3.8	DnaJ (Hsp40) homolog, subfamily B, member 9 (DNAJB9)
AJ720683	0.9	1.4	2.0	1.4	Myotubularin related protein 2 (MTMR2)
AJ720705	1.1	1.1	1.8	1.0	Shisa homolog 5 (Xenopus laevis) (RCJMB04_23m20)
AJ720717	0.8	0.8	1.2	3.0	Fatty acid binding protein 5 (FABP5)
AJ720739	1.2	2.6	3.5	2.0	Immunoresponsive 1 homolog (mouse) (IRG1)
AJ720793	1.0	1.3	0.9	1.6	Solute carrier family 7 (cationic amino acid transporter, y+ system), member 5 (RCJMB04_25m5)
AJ720813	1.5	1.4	1.2	0.5	Heterogeneous nuclear ribonucleoprotein D (AU-rich element RNA binding protein 1, 37kDa) (RCJMB04_26e18)
AJ720823	0.9	0.9	1.5	0.8	Tribbles homolog 2 (Drosophila) (TRIB2)
AJ720825	1.1	1.7	1.9	0.9	Sorting nexin 10 (SNX10)
AJ720845	0.8	1.0	1.3	3.6	RAS guanyl releasing protein 3 (calcium and DAG-regulated) (RASGRP3)
AJ720861	1.3	1.1	1.3	0.2	Lanosterol synthase (2,3-oxidosqualene-lanosterol cyclase) (LSS)
AJ720905	1.0	1.0	1.6	1.3	Kruppel-like factor 6 (KLF6)
AJ720929	1.9	0.5	0.9	0.5	Kinesin family member 23 (KIF23)
AJ720948	1.3	0.5	0.6	0.5	Cell division cycle 20 homolog (S. cerevisiae) (CDC20)
AJ720958	0.9	1.5	2.0	2.0	Ring finger and FYVE-like domain containing 1 (RFFL)
AJ720988	0.9	2.3	3.5	1.9	Transmembrane protein 123 (TMEM123)
AJ721080	1.4	0.7	0.9	0.4	Acetyl-Coenzyme A acetyltransferase 2 (acetoacetyl Coenzyme A thiolase) (ACAT2)
AJ721104	0.4	0.7	0.6	0.5	LIM domain containing 2 (LIMD2)
AJ721107	0.8	1.1	1.4	5.0	Src-like-adaptor (SLA)
AJ721110	0.5	3.3	6.5	2.5	Vanin 2 (RCJMB04_35g11)

AJ721113	1.2	4.2	5.2	2.9	Nuclear factor of kappa light polypeptide gene enhancer in B-cells inhibitor, zeta (NFKBIZ)
AJ721124	0.9	0.9	1.4	2.5	ATPase, H+ transporting, lysosomal 38kDa, V0 subunit D2 (ATP6V0D2)
AJ735439	2.3	1.1	1.6	1.1	Gallus gallus cDNA clone 8d24r7, mRNA sequence
AJ851370	1.6	1.0	1.3	0.6	Coagulation factor II (thrombin) receptor-like 1 (F2RL1)
AJ851390	1.0	0.8	1.2	0.5	Solute carrier family 20 (phosphate transporter), member 2 (RCJMB04_1f1)
AJ851395	0.9	1.2	2.3	2.0	GRIP and coiled-coil domain containing 1 (GCC1)
AJ851432	1.1	0.9	1.4	2.7	Solute carrier family 38, member 2 (SLC38A2)
AJ851480	1.2	7.3	8.7	7.3	Acyl-CoA synthetase long-chain family member 1 (ACSL1)
AJ851505	1.1	0.9	1.3	0.6	DnaJ (Hsp40) homolog, subfamily B, member 6 (DNAJB6)
AJ851506	0.7	0.8	1.1	1.5	Kelch-like 24 (Drosophila) (KLHL24)
AJ851520	1.0	0.9	1.3	0.5	Lymphocyte-specific protein 1 (LSP1)
AJ851548.1	1.1	0.7	0.7	0.4	ATP citrate lyase (ACLY)
AJ851569	1.2	1.7	2.4	1.2	Vav 3 oncogene (VAV3)
AJ851578	0.7	1.2	1.5	1.4	Pleckstrin homology domain containing, family B (evectins) member 2 (PLEKHB2)
AJ851633	0.7	0.6	0.8	1.0	Chromosome 14 open reading frame 83 (C14orf83)
AJ851647	0.9	0.9	1.2	0.4	Solute carrier family 40 (iron-regulated transporter), member 1 (RCJMB04_14o23)
AJ851669	1.1	0.7	1.2	1.4	Fibrinogen-like 2 (FGL2)
AJ851680	1.7	0.8	0.9	0.7	Fanconi anemia, complementation group C (FANCC)
AJ851705	2.0	1.4	1.5	0.9	UDP-Gal:betaGlcNAc beta 1,4- galactosyltransferase, polypeptide 6 (B4GALT6)
AJ851748	1.3	1.8	1.8	2.4	Chromatin modifying protein 2B (CHMP2B)
AJ851803	1.0	0.7	1.3	1.0	PREDICTED: similar to Moesin (Membrane-organizing extension spike protein) (RCJMB04_34k20)
AJ851808	1.2	1.4	2.0	1.0	PREDICTED: similar to KIAA0592 protein (LOC423772)
AL584098	0.5	0.6	0.3	0.1	PREDICTED: similar to Atrial natriuretic peptide clearance receptor precursor (ANP-C) (ANPRC) (NPR-C) (Atrial natriuretic peptide C-type receptor)
AW239595	1.0	1.8	2.6	1.7	PREDICTED: similar to Trappin-6 (LOC428141)
AY040527	3.1	1.1	1.3	1.1	Inhibitor of DNA binding 1, dominant negative helix-loop-helix protein (ID1)
AY237249	2.2	1.0	1.5	1.6	Bone morphogenetic protein 2 (BMP2)
AY245433	1.5	0.6	0.9	0.7	BUB1 budding uninhibited by benzimidazoles 1 homolog beta (yeast) (BUB1B)
AY265159	0.5	0.8	0.5	0.4	Lipoma HMGIC fusion partner-like 5 (LHFPL5)
AY278202	0.6	0.8	1.3	1.0	Dual specificity phosphatase 6 (DUSP6)
AY434090	0.9	1.3	1.6	0.6	Integrin, beta 5 (ITGB5)
AY450642	0.8	1.4	1.5	1.3	Phospholipase A2 receptor 1, 180kDa (PLA2R1)
AY574987	0.7	0.5	0.3	0.3	Keratin 14 (KRT14)
BI067703	4.0	0.9	2.0	1.8	Chemokine (C-C motif) ligand 17 (CCL17)
BI392244	0.7	1.6	1.3	1.1	Gallus gallus cDNA clone pgp1n.pk007.e14 5- similar to no significant hits (pLog(P) - 4), mRNA sequence; Normalized Chicken Pituitary/Hypothalamus/Pineal Library
BM440220.1	0.6	0.6	0.8	0.6	PREDICTED: hypothetical protein
BM485850	0.6	1.3	1.7	0.5	Complement component 1, r subcomponent (C1R)
BU106686	2.6	0.8	1.1	0.5	Antigen identified by monoclonal antibody Ki-67 (MKI67)
BU111203	1.4	0.5	0.8	0.9	Proliferating cell nuclear antigen (PCNA)
BU111493	1.2	0.8	1.3	1.0	Transforming growth factor, beta 2 (TGFB2)
BU111648	1.3	1.3	1.9	1.3	Microsomal glutathione S-transferase 1 (MGST1)
BU114438	1.0	1.8	1.8	1.6	PREDICTED: similar to cyclin-dependent kinase inhibitor 1C, p57 isoform 2 [Rattus norvegicus]
BU115553	1.7	1.7	2.6	4.0	Gallus gallus cDNA clone ChEST132i24 5-, mRNA sequence

BU118728	1.8	1.0	1.0	1.7	Dynein, cytoplasmic 1, intermediate chain 1 (DYNC1I1)
BU118929	0.6	0.5	0.4	0.3	Delta/notch-like EGF repeat containing (DNER)
BU120054	1.3	1.3	1.6	4.4	Rap guanine nucleotide exchange factor (GEF) 4 (RAPGEF4)
BU120465	1.2	1.6	2.1	1.9	Chimerin (chimaerin) 2 (CHN2)
BU120686	0.8	2.0	3.7	1.5	Gallus gallus cDNA clone ChEST147l12 5-, mRNA sequence
BU121809	1.3	2.1	1.4	0.9	Gallus gallus cDNA clone ChEST142g9 5-, mRNA sequence
BU122751	1.1	1.1	1.3	0.5	Gallus gallus cDNA clone ChEST148b13 5-, mRNA sequence
BU123182	0.6	0.9	1.1	1.6	Ectonucleoside triphosphate diphosphohydrolase 4 (ENTPD4)
BU124346	1.0	2.4	1.9	1.1	PREDICTED: similar to Ephx1 protein
BU135143	0.8	1.2	1.7	1.0	PREDICTED: similar to Glutamine:fructose-6-phosphate amidotransferas
BU138507	1.0	0.7	0.7	0.2	Cytochrome P450, family 51, subfamily A, polypeptide 1 (CYP51)
BU143074	1.7	1.4	2.4	3.3	PREDICTED: similar to Protein tyrosine phosphatase, non-receptor type 2 [Gallus gallus]
BU144940	0.6	0.9	2.0	3.2	Activating transcription factor 3 (ATF3)
BU145104.1	0.7	0.7	0.5	0.9	Glypican 1 (GPC1)
BU199991	0.8	1.5	1.2	1.1	Gallus gallus cDNA clone ChEST993m12 5-, mRNA sequence
BU200000	1.0	1.9	4.0	0.4	Tumor necrosis factor, alpha-induced protein 6 (TNFAIP6)
BU202662	0.7	0.7	0.5	0.6	Mab-21-like 2 (C. elegans) (MAB21L2)
BU205290	0.7	0.5	0.6	0.6	Gallus gallus cDNA clone ChEST914h14 5-, mRNA sequence
BU212825	0.7	0.6	0.6	0.2	Gallus gallus cDNA clone ChEST1003a9 5-, mRNA sequence
BU217629	1.1	1.0	1.6	2.1	Gallus gallus cDNA clone ChEST46l6 5-, mRNA sequence
BU217918	0.6	0.9	0.6	0.6	Transmembrane protein 61 (TMEM61)
BU217968	1.2	0.8	1.0	0.4	Arylsulfatase family, member J (ARSJ)
BU218715	1.2	1.5	1.5	0.7	Gallus gallus cDNA clone ChEST669p11 5-, mRNA sequence
BU221355	1.1	1.3	1.9	1.6	Gallus gallus cDNA clone ChEST662g20 5-, mRNA sequence
BU221762	1.3	0.7	0.6	0.3	LIM and cysteine-rich domains 1 (LMCD1)
BU222772	0.5	1.1	0.5	1.1	Phosphoglycerate dehydrogenase (PHGDH)
BU223203	1.4	0.5	0.7	0.5	Gallus gallus cDNA clone ChEST899h5 5-, mRNA sequence
BU229919	0.6	1.5	1.5	1.1	Junctophilin 3 (JPH3)
BU229963	1.2	0.7	0.6	0.5	Gallus gallus cDNA clone ChEST902o18 5-, mRNA sequence
BU234625	0.6	1.1	0.7	1.1	Gallus gallus cDNA clone ChEST331h23 5-, mRNA sequence
BU235342	0.7	1.8	1.8	0.8	Gallus gallus cDNA clone ChEST329f12 5-, mRNA sequence
BU239064	0.5	0.5	0.4	0.8	Betaine-homocysteine methyltransferase (BHMT)
BU241296	1.1	1.6	1.8	0.8	PREDICTED: hypothetical protein XP_429861 [Gallus gallus]
BU242338	0.9	0.5	0.7	0.7	PREDICTED: similar to YLP motif-containing protein 1 (Nuclear protein ZAP3) (ZAP113)
BU242783	0.9	1.7	2.1	1.9	Rho guanine nucleotide exchange factor (GEF) 7 (ARHGEF7)
BU246669	1.3	0.7	1.2	0.9	PREDICTED: similar to 2-phosphodiesterase isoform 2
BU253133	1.8	0.7	0.8	0.4	Gallus gallus cDNA clone ChEST646p7 5-, mRNA sequence
BU253190	1.5	1.7	2.3	1.5	Gallus gallus cDNA clone ChEST339b1 5-, mRNA sequence
BU255739	2.1	1.0	1.2	1.1	PREDICTED: similar to MCM4 minichromosome maintenance deficient 4 (S. cerevisiae)
BU258106	1.6	0.7	0.8	0.6	PREDICTED: similar to olfactory receptor MOR202-4, partial
BU259459	0.7	1.5	1.0	0.6	ATPase family, AAA domain containing 1 (ATAD1)
BU259849	1.5	0.7	0.8	0.5	Ribonucleotide reductase M2 polypeptide (RRM2)
BU260738	1.9	0.7	1.3	1.0	Kinetochore associated 1 (KNTC1)
BU261103	0.9	1.6	1.8	1.9	Differentially expressed in FDCP 6 homolog (mouse) (DEF6)

BU262875	1.2	3.2	3.3	3.6	PREDICTED: similar to carbonic anhydrase 9
BU263588	1.6	1.0	0.9	0.5	PREDICTED: similar to LOC446237 protein, partial
BU266634	2.0	0.7	0.9	0.7	PREDICTED: similar to parafibromin [Gallus gallus]
BU269038	1.0	0.8	1.0	0.4	Phosphotyrosine interaction domain containing 1 (PID1)
BU269342	0.8	0.8	0.5	0.8	Gallus gallus cDNA clone ChEST805b3 5-, mRNA sequence
BU269552	1.1	1.5	1.9	1.5	CUB domain containing protein 1 (CDCP1)
BU270966	0.6	0.6	1.2	0.3	PREDICTED: hypothetical protein XP_418509 [Gallus gallus]
BU271039	0.8	2.3	2.0	0.9	LIM domain only 2 (rhombotin-like 1) (LMO2)
BU273218	1.7	1.9	1.8	1.2	DAZ interacting protein 1 (DZIP1)
BU274538	1.1	0.9	1.7	0.5	Gallus gallus cDNA clone ChEST489c19 5-, mRNA sequence
BU277690	0.7	1.0	0.7	1.6	WD repeat domain 66 (WDR66)
BU278251	0.9	0.6	0.4	0.2	Gallus gallus cDNA clone ChEST877g11 5-, mRNA sequence
BU278451	0.9	0.8	1.0	0.4	Intraflagellar transport 57 homolog (Chlamydomonas) (IFT57)
BU278528	1.2	0.8	0.9	0.4	Tetratricopeptide repeat domain 9 (TTC9)
BU279469	1.3	1.0	0.6	0.4	Gallus gallus cDNA clone ChEST880o9 5-, mRNA sequence
BU279953	1.2	0.8	1.1	0.4	Sperm antigen with calponin homology and coiled-coil domains 1 (SPECC1)
BU280180	1.6	1.4	2.3	2.8	PREDICTED: similar to rat GCP360
BU280688	0.9	1.1	1.5	0.4	Fer-1-like 3, myoferlin (C. elegans) (FER1L3)
BU281664	1.1	0.8	0.9	0.2	Gallus gallus cDNA clone ChEST579p20 5-, mRNA sequence
BU281908	1.1	1.9	3.5	1.4	Collagen, type XXI, alpha 1 (COL21A1)
BU284059.1	0.8	1.2	0.9	1.8	PREDICTED: hypothetical protein
BU284657	1.3	0.6	0.7	0.9	Proliferation-associated 2G4, 38kDa (PA2G4)
BU286389	0.4	0.5	0.2	0.2	PREDICTED: similar to Solute carrier family 16 (monocarboxylic acid transporters), member 13, partial [Gallus gallus]
BU287966	1.4	0.7	0.8	2.7	EMG1 nucleolar protein homolog (S. cerevisiae) (EMG1)
BU291832	1.0	1.0	1.1	3.8	Cystathionase (cystathionine gamma-lyase) (CTH)
BU291905	0.9	0.7	1.3	0.8	PREDICTED: hypothetical protein
BU295027	0.7	0.7	0.9	0.3	Macrophage stimulating 1 receptor (c-met-related tyrosine kinase) (MST1R)
BU300550	1.5	1.1	1.1	0.5	Sodium channel, voltage-gated, type IX, alpha subunit (SCN9A)
BU304608	0.7	0.7	0.8	0.2	PREDICTED: similar to conserved hypothetical protein
BU305390	0.8	0.9	1.2	0.5	PREDICTED: hypothetical protein
BU305545	1.9	0.8	0.9	1.3	Non-SMC condensin II complex, subunit D3 (NCAPD3)
BU306465	0.8	2.1	3.2	2.8	Phospholipid scramblase 1 (PLSCR1)
BU306841	1.5	0.6	0.8	0.1	NAD(P) dependent steroid dehydrogenase-like (NSDHL)
BU307434	0.6	1.0	0.8	1.3	PREDICTED: similar to transcription elongation factor A (SII), 3 [Homo sapiens]
BU307848	1.3	1.1	1.8	3.4	Gallus gallus cDNA clone ChEST503k1 5-, mRNA sequence
BU307877	1.0	1.4	1.4	0.6	Gallus gallus cDNA clone ChEST506e2 5-, mRNA sequence
BU313670	0.8	1.2	0.8	3.1	Putative ISG12-1 protein (ISG12-1)
BU317012	0.7	0.7	1.2	1.1	Gallus gallus cDNA clone ChEST384b19 5-, mRNA sequence
BU317358	1.7	0.8	1.2	0.5	KIAA1913 (KIAA1913)
BU319109	1.1	1.1	2.1	0.9	Cathepsin C (CTSC)
BU320805	1.5	2.0	2.6	2.0	Asporin (ASPN)
BU321464	1.2	1.6	1.3	2.4	TIMP metallopeptidase inhibitor 4 (TIMP4)
BU322109	0.9	1.9	1.3	0.5	PREDICTED: hypothetical protein

BU323058	0.8	1.1	0.9	2.9	Musculin (activated B-cell factor-1) (MSC)
BU325184	1.2	0.5	0.5	0.4	Transmembrane protein with EGF-like and two follistatin-like domains 1 (TMEFF1)
BU325823	2.6	0.7	1.1	1.0	PREDICTED: similar to M-phase phosphoprotein 1 (LOC423793)
BU326858	0.7	0.7	0.5	0.5	Gallus gallus cDNA clone ChEST393i21 5-, mRNA sequence
BU333516	1.9	0.8	0.8	0.4	Downstream neighbor of SON (DONSON)
BU334887	1.8	0.5	0.6	0.6	Gallus gallus cDNA clone ChEST412c8 5-, mRNA sequence
BU336892	1.0	0.5	0.5	3.2	Heat shock 105kDa/110kDa protein 1 (HSPH1)
BU338496	1.5	0.9	1.0	0.5	Gallus gallus cDNA clone ChEST453h8 5-, mRNA sequence
BU338914	1.1	1.2	0.8	1.7	Gallus gallus cDNA clone ChEST454o7 5-, mRNA sequence
BU339047	1.0	0.5	0.7	0.3	Corticotropin releasing hormone receptor 2 (CRHR2)
BU339355	1.0	1.6	2.5	2.0	Gallus gallus cDNA clone ChEST451l15 5-, mRNA sequence
BU340649	0.8	0.9	0.8	0.3	Gallus gallus cDNA clone ChEST456g21 5-, mRNA sequence
BU343906	1.0	0.6	0.7	1.9	Gallus gallus cDNA clone ChEST1032h8 5-, mRNA sequence
BU347615	1.4	2.4	3.4	2.3	Regulator of telomere elongation helicase 1 (RTEL1)
BU350029	0.7	0.7	1.1	0.4	Gallus gallus cDNA clone ChEST476d4 5-, mRNA sequence
BU350156	1.0	1.0	1.6	0.9	V-ral simian leukemia viral oncogene homolog A (ras related) (RALA)
BU350636	2.2	1.1	1.2	2.6	protein tyrosine phosphatase, receptor type, O
BU351543	1.6	0.7	0.8	0.5	Fanconi anemia, complementation group L (FANCL)
BU351880	0.8	1.1	1.3	0.4	Integrin, beta 8 (ITGB8)
BU352215	1.1	1.2	1.2	0.4	Gallus gallus cDNA clone ChEST473p3 5-, mRNA sequence
BU352242	0.5	0.8	1.0	1.9	PREDICTED: similar to protein kinase BRPK
BU353622	0.9	1.3	1.7	1.2	Gallus gallus finished cDNA, clone ChEST508c4
BU353686	0.7	1.0	1.2	2.3	Gallus gallus finished cDNA, clone ChEST136j22
BU353859	0.8	1.2	2.2	1.3	Gallus gallus cDNA clone ChEST481g10 5-, mRNA sequence
BU353919	1.6	0.9	1.1	0.6	Nonhistone chromosomal protein HMG-14A (LOC422278)
BU356156	1.3	1.0	1.9	1.8	Transmembrane protein 49 (TMEM49)
BU359249	1.1	1.5	2.2	1.9	Carnitine O-octanoyltransferase (CROT)
BU362756	0.9	0.8	0.7	2.3	ATP-binding cassette, sub-family G (WHITE), member 2 (ABCG2)
BU363918	1.0	2.0	1.8	2.3	Ectonucleotide pyrophosphatase/phosphodiesterase 2 (autotaxin) (ENPP2)
BU366414	0.7	1.9	2.9	0.9	Gallus gallus cDNA clone ChEST540m5 5-, mRNA sequence
BU368845	0.9	0.6	0.4	0.4	Gallus gallus cDNA clone ChEST567m23 5-, mRNA sequence
BU373433	1.0	1.7	2.1	1.5	Dopamine receptor D4 (DRD4)
BU374834	0.8	2.0	2.6	1.5	Vanin 1 (VNN1)
BU375972	1.4	1.5	2.2	2.4	Gallus gallus cDNA clone ChEST790k18 5-, mRNA sequence
BU376215	0.5	1.2	0.6	0.3	PREDICTED: Hypothetical LOC419409 (LOC419409)
BU377399	0.9	1.4	1.2	5.3	Gallus gallus cDNA clone ChEST792o16 5-, mRNA sequence
BU379145	1.0	0.6	0.6	1.0	PREDICTED: similar to chaperonin-containing TCP-1 complex gamma chain (LOC425644)
BU380570	0.7	0.8	0.5	0.4	Solute carrier family 35, member B2 (SLC35B2)
BU382851	1.6	2.4	3.1	3.1	Gallus gallus cDNA clone ChEST862i23 5-, mRNA sequence
BU385227	1.5	0.7	0.9	0.8	Centromere protein T (CENPT)
BU386549	1.6	0.8	1.0	0.9	Diaphanous homolog 3 (Drosophila) (DIAPH3)
BU390859	1.0	1.1	0.8	0.4	Phosphoinositide-3-kinase, regulatory subunit 1 (p85 alpha) (PIK3R1)
BU391226	2.1	1.1	1.5	1.0	Centrosomal protein 152kDa (CEP152)
BU391712	0.8	1.3	1.7	1.9	ChaC, cation transport regulator homolog 1 (E. coli) (CHAC1)

BU394402	0.7	0.9	0.5	0.5	Chromosome 16 open reading frame 30 (C16orf30)
BU395153	0.9	1.5	0.9	0.8	WSC domain containing 1 (WSCD1)
BU395620	1.7	1.0	0.8	0.9	Gallus gallus cDNA clone ChEST783m9 5-, mRNA sequence
BU398229	1.0	1.6	2.0	1.3	PREDICTED: similar to beta chain spectrin
BU404404	1.3	1.9	1.6	1.8	PREDICTED: similar to Pro-Pol-dUTPase polyprotein; RNaseH; dUTPase; integrase; protease; reverse transcriptase (LOC770129), mRNA
BU405306	1.2	2.1	2.6	1.6	PREDICTED: similar to CDK5RAP2
BU405648	0.7	0.6	0.5	1.4	Protein tyrosine phosphatase, receptor type, F (PTPRF)
BU409199	4.6	1.3	4.3	2.4	Serpin peptidase inhibitor, clade B (ovalbumin), member 2 (SERPINB2)
BU409770	2.5	1.4	1.2	0.3	Hydroxymethylglutaryl-CoA synthase 1
BU411355	0.7	1.4	0.9	2.1	Growth arrest-specific 6 (GAS6)
BU413519	1.1	0.6	0.9	1.5	Chaperonin containing TCP1, subunit 5 (epsilon) (CCT5)
BU417507	1.1	0.6	0.4	0.4	Gallus gallus cDNA clone ChEST230a2 5-, mRNA sequence
BU418843	1.0	0.9	1.4	0.7	Gallus gallus cDNA clone ChEST231d2 5-, mRNA sequence
BU420127	1.0	3.0	3.7	1.7	Zinc finger CCCH-type containing 12A (ZC3H12A)
BU420182	1.5	1.8	2.8	1.1	Glutaminyl-peptide cyclotransferase (glutaminyl cyclase) (QPCT)
BU420694	0.6	18.2	26.2	3.1	Gallus gallus cDNA clone ChEST933n2 5-, mRNA sequence
BU421389	0.9	2.4	2.1	1.4	Gallus gallus cDNA clone ChEST930j3 5-, mRNA sequence
BU422376	0.9	1.3	1.3	3.4	Gallus gallus cDNA clone ChEST229h14 5-, mRNA sequence
BU425020	1.1	2.4	3.9	2.4	K123 protein (K123)
BU426499	1.8	1.0	1.4	1.2	Gallus gallus cDNA clone ChEST236h2 5-, mRNA sequence
BU433279	0.7	1.2	2.4	3.5	Gallus gallus cDNA clone ChEST218p8 5-, mRNA sequenc
BU433762	1.9	0.7	1.0	0.8	Centrosomal protein 55kDa (CEP55)
BU434803	0.9	1.6	2.0	1.4	ENSGALP00000012050 : ENSGALP00000012050 pep:novel chromosome:WASHUC1:2:15186600:15190245:-1 gene:ENSGALG00000007461 transcript:ENSGALT00000012064
BU434877	0.8	1.1	1.4	0.8	Gallus gallus cDNA clone ChEST237h23 5-, mRNA sequence
BU437856	1.1	1.3	0.9	0.5	Paired related homeobox 1 (PRRX1)
BU440496	0.9	1.3	1.0	2.1	Gallus gallus cDNA clone ChEST989a22 5-, mRNA sequence
BU440826	0.8	1.5	1.7	0.8	ATP-binding cassette, sub-family A (ABC1), member 8 (ABCA8)
BU440951	1.1	0.6	0.8	0.4	Endothelin 1 (EDN1)
BU441594	1.0	1.0	1.6	0.9	Gallus gallus cDNA clone ChEST978m1 5-, mRNA sequence
BU441936	0.7	1.5	2.5	0.6	Snail homolog 2 (Drosophila) (SNAI2)
BU442255	0.8	1.4	1.6	2.9	Stanniocalcin 2 (STC2)
BU444777	1.2	2.3	3.9	2.4	Desmoglein 2 (DSG2)
BU447021	1.0	1.5	2.1	0.7	PREDICTED: similar to elastic titin
BU448303	1.1	1.1	0.8	1.2	Gallus gallus cDNA clone ChEST192n11 5-, mRNA sequence
BU449222	0.4	0.5	0.5	0.3	PREDICTED: similar to follistatin-like 1 precursor
BU449643	0.9	1.1	0.7	1.5	Chromatin modifying protein 4C (CHMP4C)
BU452127	0.7	0.8	0.6	0.9	Gallus gallus cDNA clone ChEST203a14 5-, mRNA sequence
BU453230	1.8	1.0	1.1	0.8	Family with sequence similarity 132, member A (FAM132A)
BU456005	0.7	0.5	0.5	0.5	Gallus gallus cDNA clone ChEST203o5 5-, mRNA sequence
BU456021	1.9	1.0	0.7	0.4	Snail homolog 1 (Drosophila) (SNAI1)
BU456708	1.0	1.5	2.2	2.1	ATPase, H+ transporting, lysosomal 42kDa, V1 subunit C2 (ATP6V1C2)
BU456843	2.9	0.7	1.2	0.6	PREDICTED: hypothetical protein

BU457236	1.1	2.6	2.9	0.9	Ceruloplasmin (ferroxidase) (CP)
BU463345	0.9	1.4	1.4	0.6	PREDICTED: hypothetical protein
BU465112	0.9	1.1	0.7	0.5	Kinesin family member 25 (KIF25)
BU467932	0.5	1.2	0.9	1.1	PREDICTED: hypothetical protein
BU468099	2.5	0.9	0.9	0.4	PREDICTED: similar to histone protein Hist2h3c1
BU470096	0.6	0.9	0.7	0.3	Gallus gallus cDNA clone ChEST262e19 5-, mRNA sequence
BU471244	0.8	1.5	1.3	0.8	PREDICTED: similar to glutamine synthetase
BU472175	0.8	1.0	1.7	1.6	Gallus gallus cDNA clone ChEST678i1 5-, mRNA sequence
BU475739	1.2	1.2	0.7	0.7	Solute carrier family 2 (facilitated glucose transporter), member 12 (SLC2A12)
BU475842	0.7	0.7	1.1	0.6	G protein-coupled receptor 1 (GPR1)
BU476209	1.0	0.8	1.4	1.0	Inhibin, beta A (INHBA)
BU477418	1.3	2.3	2.8	1.9	Gallus gallus cDNA clone ChEST825n14 5-, mRNA sequence
BU481905	1.6	0.6	0.9	0.8	Gallus gallus cDNA clone ChEST831j9 5-, mRNA sequence
BU483257	1.6	0.7	1.0	0.6	PREDICTED: hypothetical protein
BU483476	1.5	0.8	0.7	0.6	Gallus gallus cDNA clone ChEST344o23 5-, mRNA sequence
BX261173	0.9	0.8	0.8	3.1	PREDICTED: similar to selenoprotein P precursor
BX261359	1.3	1.0	0.9	0.4	Fanconi anemia, complementation group A (FANCA)
BX262245	1.8	1.0	1.7	1.0	Palladin, cytoskeletal associated protein (PALLD)
BX262539	1.0	0.8	1.0	0.4	Gallus gallus cDNA clone gcal0008.g.13 3prim, mRNA sequence; Gallus gallus multi-tissues normalized and once-subtracted cDNA library (gcal)
BX262827	0.5	0.7	1.0	0.9	Kruppel-like factor 2 (lung) (KLF2)
BX266231	0.7	0.7	0.4	1.0	Gallus gallus cDNA clone gcal0011.j.24 3prim, mRNA sequence; Gallus gallus multi-tissues normalized and once-subtracted cDNA library (gcal)
BX266352	1.2	1.5	1.4	0.6	Suppression of tumorigenicity 14 (colon carcinoma) (ST14)
BX267574	1.0	1.5	1.8	4.2	PREDICTED: similar to stem cell antigen 2
BX268699	0.6	1.3	1.1	1.4	Tetratricopeptide repeat domain 16 (TTC16)
BX271230	0.7	1.1	0.9	0.4	Gallus gallus cDNA clone gcal0001.h.19 3prim, mRNA sequence; Gallus gallus multi-tissues normalized and once-subtracted cDNA library (gcal)
BX272499	0.5	0.5	0.6	0.5	Wingless-type MMTV integration site family, member 2B (WNT2B)
BX272837	1.1	0.6	1.0	0.4	Calpain 9 (CAPN9)
BX273853	0.8	1.6	1.7	1.4	Gallus gallus cDNA clone gcal0005.g.17 5prim, mRNA sequence; Gallus gallus multi-tissues normalized and once-subtracted cDNA library (gcal)
BX276973	0.8	1.1	0.8	0.3	Gallus gallus cDNA clone gcag0009.n.04 5prim, mRNA sequence; Gallus gallus multi-tissues normalized library (gcag)
BX277390	1.4	0.7	0.8	0.5	Gallus gallus cDNA clone gcag0009.g.04 5prim, mRNA sequence; Gallus gallus multi-tissues normalized library (gcag)
BX279056	1.4	0.6	0.8	0.5	PREDICTED: similar to Brrn1-prov protein
BX929282	1.2	1.7	2.0	3.9	Thioesterase superfamily member 2 (THEM2)
BX929599	1.1	2.4	1.9	1.1	Growth arrest and DNA-damage-inducible, gamma (GADD45G)
BX929654.1	1.3	1.2	1.8	1.1	Retinoic acid induced 14 (RAI14)
BX929698	1.1	0.9	1.4	0.9	PREDICTED: similar to steroid dehydrogenase
BX929804	1.1	1.6	1.9	0.7	Superoxide dismutase 3, extracellular (SOD3)
BX929845	0.7	1.2	1.0	0.5	Fin bud initiation factor (FIBIN)
BX929886	0.8	0.9	0.7	0.3	Glycosyltransferase 8 domain containing 2 (GLT8D2)

BX929945	1.9	0.6	0.8	0.5	Cyclin-dependent kinase inhibitor 3 (CDK2-associated dual specificity phosphatase) (CDKN3)
BX930046	1.3	1.3	2.1	1.1	Tetratricopeptide repeat domain 19 (TTC19)
BX930047	0.9	1.2	1.2	3.3	Aquaporin 9 (AQP9)
BX930055	0.6	1.0	0.9	1.4	Gallus gallus finished cDNA, clone ChEST985n16
BX930091	0.7	1.2	0.8	0.9	Gallus gallus finished cDNA, clone ChEST1017a23
BX930115	1.1	1.6	1.4	4.6	Ankyrin repeat domain 22 (ANKRD22)
BX930147	1.2	1.0	1.7	0.9	Peroxiredoxin 1 (PRDX1)
BX930215	0.5	0.9	0.6	0.4	Response gene to complement 32 (LOC418833)
BX930231	0.4	0.8	0.7	0.4	Family with sequence similarity 20, member C (FAM20C)
BX930261	0.9	1.2	0.9	2.2	2,4-dienoyl CoA reductase 1, mitochondrial (DECR1)
BX930311	0.6	0.5	0.3	0.3	Potassium channel tetramerisation domain containing 12 (KCTD12)
BX930357	0.4	0.8	0.7	0.5	Dehydrogenase/reductase (SDR family) member 2 (DHRS2)
BX930367	0.7	1.6	1.9	1.6	Bactericidal/permeability-increasing protein (BPI)
BX930381	0.9	0.6	0.7	0.5	Epithelial membrane protein 1 (EMP1)
BX930456	0.9	4.4	3.0	3.3	RAS, dexamethasone-induced 1 (RASD1)
BX930754	1.4	0.9	1.4	0.7	N-acetylglucosamine-1-phosphate transferase, alpha and beta subunits (GNPTAB)
BX930996	1.8	1.1	1.9	1.4	Angiopoietin-like 2 (ANGPTL2)
BX931007	0.8	1.2	0.9	1.7	Aldehyde dehydrogenase 7 family, member A1 (ALDH7A1)
BX931154	0.7	1.6	1.2	0.6	PREDICTED: similar to Chromosome 16 open reading frame 77
BX931191	1.0	2.5	3.6	1.3	PREDICTED: similar to normal mucosa of esophagus specific 1 (LOC415442)
BX931246	0.8	1.3	1.3	2.6	Tripartite motif-containing 63 (TRIM63)
BX931288	2.1	0.6	0.8	0.6	Ubiquitin-like, containing PHD and RING finger domains, 1 (UHRF1)
BX931291	1.0	0.7	1.0	0.7	Selenoprotein S (SELS)
BX931297	1.2	1.5	0.8	0.4	Cytokine-like 1 (CYTL1)
BX931352	0.8	1.2	1.5	1.9	PREDICTED: similar to stem cell antigen 2
BX931404	0.8	1.4	1.6	1.1	Mdm2, transformed 3T3 cell double minute 2, p53 binding protein (mouse) (MDM2)
BX931418	0.8	0.8	1.0	2.9	IKK interacting protein (IKIP)
BX931532	1.3	1.6	2.2	2.4	Cystatin B (stefin B) (CSTB)
BX931577	0.9	0.8	0.5	0.4	Glyoxylate reductase/hydroxypyruvate reductase (GRHPR)
BX931663	0.7	2.7	1.1	5.6	Ropporin 1-like (ROPN1L)
BX931790	0.9	2.3	2.2	1.4	Tumor necrosis factor receptor superfamily, member 6b, decoy (TNFRSF6B)
BX931940	0.7	1.4	1.2	0.8	Translocator protein (18kDa) (TSPO)
BX931971	1.6	2.8	4.0	12.1	Spondin 2, extracellular matrix protein (SPON2)
BX932207	2.9	1.4	1.6	1.3	A kinase (PRKA) anchor protein 7 (AKAP7)
BX932212	2.1	0.7	0.8	0.3	Pituitary tumor-transforming 1 (PTTG1)
BX932293	0.6	0.6	0.5	0.2	Heat shock 27kDa protein family, member 7 (cardiovascular) (HSPB7)
BX932384	1.2	0.5	0.7	0.4	Cell division cycle associated 3 (CDCA3)
BX932426	1.0	1.4	1.7	0.6	Leucine rich repeat containing 6 (LRRC6)
BX932427	1.2	3.1	2.9	4.9	Basic leucine zipper transcription factor, ATF-like 3 (BATF3)
BX932445	0.7	1.1	1.1	2.1	IKK interacting protein (IKIP)
BX932547	0.8	0.7	0.6	2.2	Phosphoserine phosphatase (PSPH)
BX932834	1.6	0.5	0.7	0.4	Thymidylate synthetase (TYMS)
BX932979	2.0	0.9	1.0	0.5	Centromere protein P (CENPP)
BX933015	1.6	0.9	0.8	0.7	Apoptosis-inducing, TAF9-like domain 1 (APITD1)

BX933041	1.3	1.2	1.0	0.5	Gallus gallus finished cDNA, clone ChEST997e1
BX933127	2.0	0.7	0.9	0.6	Kinesin family member 15 (KIF15)
BX933215	3.9	1.5	2.9	3.3	Suppressor of cytokine signaling 1 (SOCS1)
BX933315	0.7	1.4	1.4	2.2	KH domain containing, RNA binding, signal transduction associated 3 (KHDRBS3)
BX933437	0.8	1.5	1.0	2.5	Fibrinogen-like 1 (FGL1)
BX933454	0.9	1.1	1.7	4.2	Prokineticin 2 (PROK2)
BX933582	0.6	1.3	1.0	1.1	PREDICTED: similar to TGF-beta type II receptor
BX933595	0.5	1.1	1.0	0.6	Sulfotransferase family 1E, estrogen-preferring, member 1 (SULT1E1)
BX933725	1.2	1.1	1.5	0.6	Annexin A1 (ANXA1)
BX933739	1.2	0.6	0.7	0.7	PREDICTED: hypothetical protein
BX933888	0.9	1.8	1.6	0.6	C1q and tumor necrosis factor related protein 3 (C1QTNF3)
BX933946	1.1	1.6	1.2	0.8	BX933946
BX933994	0.8	1.2	1.1	2.6	Tryptophanyl-tRNA synthetase (WARS)
BX934024	1.7	0.6	0.8	0.5	PDZ binding kinase (PBK)
BX934061	1.6	1.3	2.2	1.0	BMP and activin membrane-bound inhibitor homolog (Xenopus laevis) (BAMBI)
BX934073	0.9	0.9	1.4	0.4	Ras-related associated with diabetes (RRAD)
BX934082	2.1	0.8	0.9	0.7	Cancer susceptibility candidate 5 (CASC5)
BX934121	0.9	1.5	2.2	5.5	Tissue factor pathway inhibitor 2 (TFPI2)
BX934150	0.7	1.4	1.7	0.6	PREDICTED: similar to Pyridoxal (pyridoxine, vitamin B6) kinase, partial
BX934532	0.9	1.5	2.3	1.2	Gallus gallus finished cDNA, clone ChEST158l24
BX934666	1.0	0.9	1.2	0.4	WNT1 inducible signaling pathway protein 1 (WISP1)
BX934937	0.5	0.6	0.5	0.6	Hydroxyprostaglandin dehydrogenase 15-(NAD) (HPGD)
BX935001	0.5	0.7	0.7	0.3	Selenium binding protein 1 (SELENBP1)
BX935011	0.8	0.9	1.1	0.4	Fumarylacetoacetate hydrolase (fumarylacetoacetase) (FAH)
BX935026	0.7	1.0	0.7	3.5	Methionine adenosyltransferase I, alpha (MAT1A)
BX935060	0.9	0.5	0.3	0.3	PREDICTED: similar to Keratin, type I cytoskeletal 18 (Cytokeratin 18) (K18) (CK 18) isoform 1
BX935069	0.8	1.7	1.8	1.2	PREDICTED: similar to NAD(P)H dehydrogenase, quinone 1 (LOC769737)
BX935110	1.8	0.9	1.9	0.4	PREDICTED: similar to PA2.26 antigen isoform 2
BX935133	0.6	0.7	0.7	0.3	PREDICTED: chloride intracellular channel 3
BX935204.2	0.7	0.7	1.0	0.6	PREDICTED: similar to folylpoly-gamma-glutamate carboxypeptidase
BX935349	0.5	0.4	0.5	0.1	Complement component 1, q subcomponent, B chain (C1QB)
BX935378	1.0	1.1	2.1	2.6	Ankyrin repeat domain 9 (ANKRD9)
BX935484	0.7	0.7	0.7	1.7	Phosphoserine aminotransferase 1 (PSAT1)
BX935496	0.6	1.1	0.8	1.2	Sarcospan (Kras oncogene-associated gene) (SSPN)
BX935550	0.6	2.9	2.7	1.2	Aldo-keto reductase family 1, member D1 (delta 4-3-ketosteroid-5-beta-reductase) (AKR1D1)
BX935571	0.6	0.8	0.6	0.3	Fibulin 5 (FBLN5)
BX935573	0.9	0.8	1.3	0.8	Superoxide dismutase 1, soluble (amyotrophic lateral sclerosis 1 (adult)) (SOD1)
BX935591	1.0	1.7	2.1	2.1	Ninjurin 1 (NINJ1)
BX935595	1.3	0.7	0.9	0.4	MAD2 mitotic arrest deficient-like 1 (yeast) (MAD2L1)
BX935712	1.5	0.9	0.7	0.6	PREDICTED: similar to Cell division cycle associated 8, partial
BX935863	1.2	1.8	3.0	3.1	Peripheral myelin protein 2 (PMP2)
BX935864	0.9	0.8	0.7	3.4	X-box binding protein 1 (XBP1)
BX935985	0.9	0.8	1.4	0.9	Gallus gallus finished, clone ChEST431o2
BX936026	2.4	0.5	0.7	1.0	Aurora kinase A (AURKA)

BX936224	0.5	0.8	0.5	0.3	Cysteine dioxygenase, type I (CDO1)
BX936276	0.5	0.5	0.5	0.8	PREDICTED: similar to DnaJ-like protein
BX950396	1.9	1.8	2.5	1.0	Matrix-remodelling associated 5 (MXRA5)
BX950437	0.7	0.9	0.7	0.3	Butyrobetaine (gamma), 2-oxoglutarate dioxygenase (gamma-butyrobetaine hydroxylase) 1 (BBOX1)
BX950502	1.4	0.8	1.6	0.8	Pleckstrin homology-like domain, family A, member 2 (PHLDA2)
BX950642	1.1	1.5	1.9	1.0	PREDICTED: similar to RAB18, member RAS oncogene family
BX950656	1.2	0.6	0.6	0.7	Receptor accessory protein 1 (REEP1)
BX950657	1.1	1.3	2.5	0.3	PREDICTED: chemokine (C-C motif) receptor-like 1 isoform 1
BX950711.1	1.0	1.2	0.8	1.5	Selenoprotein X, 1 (SEPX1)
BX950745	0.9	1.0	1.1	0.4	Olfactomedin-like 3 (OLFML3)
BX950762	2.0	0.7	0.9	0.6	Ubiquitin-conjugating enzyme E2T (putative) (UBE2T)
CB270855.1	0.7	0.7	0.5	0.4	High density lipoprotein binding protein (vigilin) (HDLBP)
CD728822	1.0	1.4	1.9	1.6	PREDICTED: similar to TPA-induced transmembrane protein
CD729265	1.0	0.7	1.0	0.4	PREDICTED: similar to PDNP1
CD763113	1.1	0.6	0.7	0.2	Farnesyl diphosphate synthase (farnesyl pyrophosphate synthetase, dimethylallyltranstransferase, geranyltranstransferase) (FDPS)
CD764582	0.5	0.7	1.0	1.1	Limb Bud - LB1 Gallus gallus cDNA clone GGEZLB1017E09, mRNA sequence
CF250950	0.6	2.1	2.3	3.6	Aldehyde dehydrogenase 1 family, member A3 (ALDH1A3)
CF251376	0.6	1.0	0.5	0.8	Dermatopontin (DPT)
CF254917	1.4	0.7	1.0	0.3	SHC (Src homology 2 domain containing) family, member 4 (SHC4)
CK609466	1.7	1.4	1.4	0.6	Gallus gallus cDNA clone IFNi_N09 5-, mRNA sequence
CK609691	1.0	2.0	1.9	1.5	IFNk_G10 Interferon Stimulated Chicken PBL Macrophage Gallus gallus cDNA clone IFNk_G10 5-, mRNA sequence
CK609854	0.9	1.6	2.1	1.4	Cystatin A (stefin A) (CSTA)
CK610423	1.2	2.0	2.9	2.7	Chemokine ah221 (LOC417536)
CK612370	1.2	0.9	1.5	0.9	Dermatan sulfate epimerase (DSE)
CN210627.1	0.8	1.4	1.9	1.0	Odz, odd Oz/ten-m homolog 4 (Drosophila) (ODZ4)
CN218923.1	1.1	1.3	0.9	5.5	Cdc42 guanine nucleotide exchange factor (GEF) 9 (ARHGEF9)
CN219930	0.8	0.7	1.1	2.1	Transcribed locus, weakly similar to NP_001007009.1 coiled-coil-helix-coiled-coil-helix domain containing 10 [Rattus norvegicus]
CN223734	0.7	1.2	1.4	1.3	Gallus gallus finished cDNA, clone ChEST472d7
CN229430	1.0	1.3	0.9	3.9	DEAD (Asp-Glu-Ala-Asp) box polypeptide 4 (DDX4)
CO505470	0.8	0.6	0.5	0.3	SPARC related modular calcium binding 2 (SMOC2)
CO506634	0.6	0.8	0.5	0.4	Embryo breast muscle - EB1 Gallus gallus cDNA clone GGEZEB1021B04, mRNA sequence
CO635775	0.8	0.5	0.5	2.6	Heat shock protein 90kDa alpha (cytosolic), class A member 1 (HSP90AA1)
CO760996	0.8	1.1	0.8	2.0	Aldehyde dehydrogenase 1 family, member L2 (ALDH1L2)
CR338704	1.1	0.6	0.9	1.3	Gallus gallus finished cDNA, clone ChEST99d11
CR338819	1.1	0.9	1.2	0.5	Gallus gallus finished cDNA, clone ChEST963b22
CR352395	0.8	0.9	0.9	4.2	Osteocrin (OSTN)
CR352420	1.0	1.1	0.9	2.4	Guanine nucleotide binding protein (G protein), alpha z polypeptide (GNAZ)
CR352647	2.7	1.3	1.6	1.4	Gallus gallus finished cDNA, clone ChEST424l5
CR352660	2.3	1.7	2.2	2.1	Chromosomal passenger complex protein Dasra A (DASRAA)
CR352752	0.8	1.7	2.5	1.8	Mdm2, transformed 3T3 cell double minute 2, p53 binding protein (mouse) (MDM2)
CR352822	2.0	1.8	3.0	1.9	IQ motif and Sec7 domain 1 (IQSEC1)

CR352895	1.7	0.5	0.6	0.4	PREDICTED: similar to aldolase C
CR353337	1.2	1.1	2.0	0.8	Tissue factor pathway inhibitor (lipoprotein-associated coagulation inhibitor) (TFPI)
CR353415	0.5	1.0	1.4	0.4	Secreted phosphoprotein 1 (osteopontin, bone sialoprotein I, early T-lymphocyte activation 1) (SPP1)
CR353484	0.9	1.3	2.0	0.7	Chromosome 9 open reading frame 91 (C9orf91)
CR353501	0.9	1.1	1.6	1.3	Proline rich 5 (renal) (PRR5)
CR353524	1.7	0.6	0.7	0.8	Atonal homolog 8 (Drosophila) (ATOH8)
CR353526	0.9	2.3	2.4	2.5	Gallus gallus finished cDNA, clone ChEST132b22
CR354068	1.5	0.8	1.0	0.7	Centromere protein K (CENPK)
CR354132	0.7	0.5	0.9	1.7	Gallus gallus finished cDNA, clone ChEST385h5
CR354135	0.7	2.1	2.2	0.8	Gallus gallus finished cDNA, clone ChEST387p23
CR354151	1.6	0.5	0.6	0.5	Gallus gallus finished cDNA, clone ChEST380p8
CR354286	1.0	1.7	2.6	3.5	Ubiquitin specific peptidase 18 (USP18)
CR354333	0.8	1.4	2.9	1.0	Gallus gallus finished cDNA, clone ChEST75j21
CR354334	1.6	0.7	0.8	0.6	PREDICTED: similar to chromosome transmission fidelity factor 8 homolog
CR354360	1.4	0.8	0.8	0.5	Hydrolethalus syndrome 1 (HYLS1)
CR354361	0.8	1.6	1.9	0.6	Fibroblast growth factor 7 (keratinocyte growth factor) (FGF7)
CR382434	1.1	2.0	1.5	1.2	Phosphatidic acid phosphatase type 2A (PPAP2A)
CR382435	0.8	0.9	0.9	5.1	Hepatoma-derived growth factor, related protein 3 (HDGFRP3)
CR385124	1.3	0.9	1.0	0.3	7-dehydrocholesterol reductase (DHCR7)
CR385142	0.6	0.6	0.7	0.4	PREDICTED: hypothetical protein
CR385166	0.6	1.0	1.1	5.7	V-myc myelocytomatosis viral related oncogene, neuroblastoma derived (avian) (MYCN)
CR385186	2.2	0.5	0.8	0.4	PREDICTED: similar to CUG2
CR385201	0.6	5.0	7.0	0.6	Gallus gallus finished cDNA, clone ChEST750f11
CR385332	0.8	0.7	0.8	0.3	SPARC related modular calcium binding 1 (SMOC1)
CR385367	1.8	0.9	1.3	0.6	KIAA1913 (KIAA1913)
CR385491	1.3	0.8	0.9	0.2	Isopentenyl-diphosphate delta isomerase 1 (IDI1)
CR385494	0.5	0.9	0.7	0.7	Gallus gallus finished cDNA, clone ChEST973f18
CR385495	2.2	1.4	2.7	2.7	Gallus gallus finished cDNA, clone ChEST379k15
CR385514	1.1	1.0	1.1	0.3	Chromosome 10 open reading frame 72 (C10orf72)
CR385527	1.9	0.8	0.7	0.6	Gallus gallus finished cDNA, clone ChEST375n1
CR385559	1.7	1.7	2.0	1.4	Gallus gallus finished cDNA, clone ChEST376d11
CR385581	2.1	0.7	0.9	0.8	Helicase, lymphoid-specific (HELLS)
CR385622	1.1	0.9	0.6	0.4	Gallus gallus finished cDNA, clone ChEST855o4
CR385678	1.1	1.3	1.8	5.5	Gallus gallus finished cDNA, clone ChEST876i3
CR385698	0.9	1.7	2.0	1.0	Gallus gallus finished cDNA, clone ChEST571j17
CR385721	1.9	1.5	1.9	1.1	Gallus gallus finished cDNA, clone ChEST508l10
CR385747	1.4	1.6	1.7	0.8	Gallus gallus finished cDNA, clone ChEST516e12
CR385787	0.8	0.5	0.4	0.4	Olfactomedin 3 (OLFM3)
CR385831	0.6	1.3	1.3	1.2	Sideroflexin 3 (SFXN3)
CR385841	1.3	1.1	1.2	0.4	Gallus gallus finished cDNA, clone ChEST704d18
CR385852	1.0	1.1	1.7	2.8	Gallus gallus finished cDNA, clone ChEST206a7
CR385948	0.7	1.3	1.4	1.8	Gallus gallus finished cDNA, clone ChEST156k5
CR386009	0.7	1.6	2.9	1.9	Gallus gallus finished cDNA, clone ChEST213b13
CR386034	1.2	1.8	2.0	2.6	PREDICTED: similar to RP5-1022P6.2, partial

CR386133	0.7	0.7	0.5	0.7	Gallus gallus finished cDNA, clone ChEST364n9
CR386282	0.7	1.0	1.0	0.4	Collagen, type XII, alpha 1 (COL12A1)
CR386318	0.7	1.3	1.0	0.7	PREDICTED: hypothetical protein LOC379587; programmed cell death 4
CR386411	1.4	1.7	2.4	1.8	Gallus gallus finished cDNA, clone ChEST225o19
CR386459	0.9	1.6	1.5	1.6	Marker protein (Ch21)
CR386489	1.1	0.3	0.4	1.0	KH domain containing, RNA binding, signal transduction associated 2 (KHDRBS2)
CR386526	0.9	1.3	1.7	1.6	Gallus gallus finished cDNA, clone ChEST334o14
CR386594	0.7	0.6	0.8	0.7	Gallus gallus finished cDNA, clone ChEST762j22
CR386671	1.8	0.8	1.1	1.6	RAP1 interacting factor homolog (yeast) (RIF1)
CR386687	0.7	0.9	1.2	2.1	Gallus gallus finished cDNA, clone ChEST185l21
CR386703	1.9	1.8	2.0	1.0	Gallus gallus finished cDNA, clone ChEST862a10
CR386845	1.0	0.8	0.7	3.1	Gallus gallus finished cDNA, clone ChEST38l2
CR386878	0.8	1.2	1.1	0.4	Gallus gallus finished cDNA, clone ChEST41l22
CR386923	0.5	0.6	1.2	0.6	Gallus gallus finished cDNA, clone ChEST992o1
CR386957	1.2	0.9	1.3	0.5	Gallus gallus finished cDNA, clone ChEST108i11
CR387033	0.9	1.0	1.6	1.1	Gallus gallus finished cDNA, clone ChEST434d19
CR387035	2.0	1.0	1.4	1.3	Gallus gallus finished cDNA, clone ChEST432l20
CR387087	0.5	1.0	1.0	1.7	Gallus gallus finished cDNA, clone ChEST496j22
CR387244.1	0.8	1.2	1.3	2.2	PREDICTED: hypothetical protein; Golli-mbp isoform 2
CR387256	1.3	1.4	2.2	2.1	Gallus gallus finished cDNA, clone ChEST140e21
CR387287	0.9	0.9	1.5	1.5	Dual specificity phosphatase 1 (DUSP1)
CR387407	3.4	1.6	2.2	0.9	PREDICTED: similar to LOC129881 protein
CR387420	1.1	0.9	0.6	0.9	RAB3A interacting protein (rabin3) (RAB3IP)
CR387448	0.7	0.6	0.4	1.9	Ubiquitin carboxyl-terminal esterase L1 (ubiquitin thiolesterase) (UCHL1)
CR387450	1.4	0.6	0.8	0.6	Gallus gallus finished cDNA, clone ChEST487d8
CR387485	0.7	1.5	2.0	1.3	Gallus gallus finished cDNA, clone ChEST305m14
CR387505	0.4	0.7	0.8	0.7	Solute carrier family 24 (sodium/potassium/calcium exchanger), member 6 (SLC24A6)
CR387701	1.2	1.7	1.8	0.8	MICAL-like 2 (MICALL2)
CR387745	1.0	1.4	1.4	3.3	Gallus gallus finished cDNA, clone ChEST532o15
CR387761	1.2	0.7	0.8	0.2	PREDICTED: similar to Gap junction alpha-7 protein (Connexin 45) (Cx45) [Gallus gallus]
CR387838	1.0	0.7	1.2	0.5	Gallus gallus finished cDNA, clone ChEST608p22
CR387903	1.8	1.1	1.0	0.4	Gallus gallus finished cDNA, clone ChEST175o2
CR387914	1.1	0.8	0.7	0.2	Chondroadherin (CHAD)
CR388632	0.7	1.4	0.8	0.2	Gallus gallus finished cDNA, clone ChEST845e14
CR388639	0.9	1.2	1.3	3.1	Gallus gallus finished cDNA, clone ChEST628g18
CR388879	0.6	0.8	0.6	0.3	Gallus gallus finished cDNA, clone ChEST84l23
CR388945	1.0	1.2	0.7	0.4	Gallus gallus finished cDNA, clone ChEST874b2
CR388971	0.8	0.9	0.9	2.7	Protocadherin 7 (PCDH7)
CR388986	0.7	1.2	0.8	1.4	Sideroflexin 1 (SFXN1)
CR389027	0.9	2.7	2.5	2.3	Gallus gallus finished cDNA, clone ChEST841o6
CR389058	1.2	0.8	1.1	0.4	NUAK family, SNF1-like kinase, 1 (NUAK1)
CR389094	0.8	1.3	1.5	1.3	6-phosphofructo-2-kinase/fructose-2,6-biphosphatase 4 (PFKFB4)
CR389140	0.9	1.4	1.2	2.9	Gallus gallus finished cDNA, clone ChEST280d18
CR389281	0.8	0.8	0.9	0.3	Choline phosphotransferase 1 (CHPT1)

CR389319	2.0	1.4	1.9	1.3	Gallus gallus finished cDNA, clone ChEST731d1
CR389443	1.1	1.4	1.6	0.7	Gallus gallus finished cDNA, clone ChEST184g12
CR389475	0.6	0.5	0.5	0.3	PREDICTED: similar to Coiled-coil domain containing 102A, partial
CR389509	0.6	0.6	0.4	0.3	Gallus gallus finished cDNA, clone ChEST186b22
CR389517	1.1	1.6	2.7	1.5	Gallus gallus finished cDNA, clone ChEST983o22
CR389612	0.5	0.7	0.7	1.6	Gallus gallus finished cDNA, clone ChEST559o9
CR389704	0.5	0.6	0.6	0.5	FAD-dependent oxidoreductase domain containing 1 (FOXRED1)
CR389767	1.2	3.2	3.2	0.5	Gallus gallus finished cDNA, clone ChEST721b7
CR389813	1.0	1.3	1.2	5.9	Gallus gallus finished cDNA, clone ChEST269m14
CR389889	0.7	1.1	1.5	0.8	ch-runtB2
CR389935	2.2	1.6	1.8	2.4	PREDICTED: hypothetical protein
CR390162	0.7	0.9	0.6	0.3	Gallus gallus finished cDNA, clone ChEST724o21
CR390178	1.7	1.1	1.6	0.4	Gallus gallus finished cDNA, clone ChEST930g18
CR390179	1.0	1.2	1.8	1.1	XP_427539 : gi:50782513:ref:XP_427539.1: PREDICTED: similar to 68 kDa type I phosphatidylinositol-4-phosphate 5-kinase alpha [Gallus gallus]
CR390208	0.9	0.7	0.5	0.6	SLIT and NTRK-like family, member 2 (SLITRK2)
CR390337	1.1	1.1	1.4	0.5	Suppression of tumorigenicity 5 (ST5)
CR390375	0.7	0.5	0.5	0.6	PREDICTED: similar to amino acid transporter system A1
CR390519	1.4	1.5	1.8	8.3	Gallus gallus finished cDNA, clone ChEST567a23
CR390562	1.2	1.2	1.3	6.2	PREDICTED: hypothetical protein
CR390609	2.0	0.8	0.9	1.0	Gallus gallus finished cDNA, clone ChEST1029i12
CR390654	0.9	0.7	0.8	1.8	Oligonucleotide/oligosaccharide-binding fold containing 2A (OBFC2A)
CR390810	0.8	1.4	2.1	0.9	DNA-dependent protein kinase catalytic subunit (Fragment)
CR390841	1.0	0.5	0.6	0.9	Peripheral myelin protein 22 (PMP22)
CR390858	2.1	1.1	1.3	1.0	Gallus gallus finished cDNA, clone ChEST234e18
CR390935	1.0	1.5	1.3	0.7	TRAF3 interacting protein 2 (TRAF3IP2)
CR390951	1.5	0.6	0.7	0.3	Chromosome 8 open reading frame 79 (C8orf79)
CR390976	0.8	1.5	1.4	1.3	PREDICTED: similar to C20orf194
CR391100	0.3	3.0	4.1	0.4	Gallus gallus finished cDNA, clone ChEST202c2
CR391234	1.5	0.3	0.6	0.2	Lung lectin (LL)
CR391404	0.7	0.8	0.5	0.2	Integrin, alpha 8 (ITGA8)
CR391426	0.8	1.6	0.9	0.7	Fibulin 2 (FBLN2)
CR391470	0.9	0.6	0.6	0.4	Gallus gallus finished cDNA, clone ChEST771j1
CR391580	0.7	0.8	0.6	0.3	Gallus gallus finished cDNA, clone ChEST801i22
CR391749	0.7	1.4	1.5	1.0	Glycoprotein M6B (GPM6B)
CR405944	1.4	2.5	2.9	3.3	CD44 molecule (Indian blood group) (CD44)
CR405963	0.5	0.8	0.5	0.3	PREDICTED: similar to HWKM1940
CR406056	1.0	1.7	2.1	1.3	Proprotein convertase subtilisin/kexin type 5
CR406066	0.8	1.6	1.4	0.8	Ninjurin 2 (NINJ2)
CR406132	2.1	1.7	1.8	2.7	Aspartate beta-hydroxylase (ASPH)
CR406175	1.0	0.5	0.5	0.4	PREDICTED: similar to MSTP030; similar to ribosomal protein L5
CR406246	0.8	0.6	0.5	1.0	Chromosome 9 open reading frame 150 (C9orf150)
CR406252	1.2	1.1	1.4	4.7	Prematurely terminated mRNA decay factor-like (LOC428770)
CR406298	2.1	0.9	1.2	0.7	Retinoblastoma-like 1 (p107) (RBL1)

CR406322	0.7	0.6	0.8	0.6	Frizzled homolog 8 (Drosophila) (FZD8)
CR406335	1.0	1.2	1.9	1.4	Notum pectinacetylesterase homolog (Drosophila) (NOTUM)
CR406359	0.9	1.4	2.1	1.3	Glucosamine (N-acetyl)-6-sulfatase (Sanfilippo disease IIID) (GNS)
CR406404	0.9	0.6	0.5	0.6	Gallus gallus finished cDNA, clone ChEST834i13
CR406411	0.9	1.2	1.7	0.6	PREDICTED: similar to KIAA1700 protein, partial
CR406447.1	0.9	0.4	0.4	0.8	KH domain containing, RNA binding, signal transduction associated 2 (KHDRBS2)
CR406496	0.9	0.6	0.7	1.1	Transmembrane protein with EGF-like and two follistatin-like domains 2 (TMEFF2)
CR406511	1.0	0.6	0.9	1.2	Gallus gallus finished cDNA, clone ChEST734o14
CR406543	1.0	0.9	1.1	4.1	Selenoprotein O (SELO)
CR406552	1.4	1.7	2.6	1.7	Gallus gallus finished cDNA, clone ChEST680j17
CR406603	2.2	2.1	2.8	1.1	Gallus gallus finished cDNA, clone ChEST648g9
CR406622	2.4	1.2	1.3	1.7	Gallus gallus finished cDNA, clone ChEST761o6
CR406752	0.6	2.0	2.1	0.7	Glutamate-ammonia ligase (glutamine synthetase) (GLUL)
CR406802	2.0	1.1	1.9	1.1	Gallus gallus finished cDNA, clone ChEST733j10
CR406806	0.5	0.9	1.0	0.6	Gallus gallus finished cDNA, clone ChEST725l11
CR406810	1.7	1.6	1.7	1.0	Peroxidasin homolog (Drosophila) (PXDN)
CR406822	1.1	1.6	1.9	2.3	Gallus gallus finished cDNA, clone ChEST728a19
CR406836	0.6	1.3	1.1	1.1	Gallus gallus finished cDNA, clone ChEST737i17
CR406894	1.3	1.3	1.8	0.9	SMAD family member 7 (SMAD7)
CR406946	1.6	0.7	0.9	0.3	Gallus gallus finished cDNA, clone ChEST909p20
CR407116	0.6	1.9	2.2	1.0	Gallus gallus finished cDNA, clone ChEST752o10
CR407207	0.7	0.4	0.5	0.2	Fatty acid desaturase 2 (FADS2)
CR407225	1.1	0.7	0.7	0.4	Nexilin (F actin binding protein) (NEXN)
CR407328	0.9	2.0	1.7	2.4	Gallus gallus finished cDNA, clone ChEST911n23
CR407399	0.4	1.2	1.3	0.4	Argininosuccinate synthetase 1 (RCJMB04_21j5)
CR407416	2.0	0.5	0.9	0.5	PREDICTED: similar to RIKEN cDNA 2700049P18 gene
CR407473	1.1	1.6	1.0	2.0	PREDICTED: similar to MGC80370 protein
CR407493	1.0	1.9	1.4	1.2	BCL2/adenovirus E1B 19kDa interacting protein 3 (BNIP3)
CR407528	0.8	1.1	1.6	0.8	Gallus gallus finished cDNA, clone ChEST662i24
CR522951	1.6	1.0	1.3	0.5	Gallus gallus finished cDNA, clone ChEST738f3
CR522990	1.3	1.4	2.1	2.7	Gallus gallus finished cDNA, clone ChEST800j11
CR523007	0.9	1.1	0.9	2.6	PREDICTED: similar to erythrocyte membrane protein band 4.1 (elliptocytosis 1, RH-linked)
CR523033	2.2	1.2	1.3	1.2	Gallus gallus finished cDNA, clone ChEST898k7
CR523095	1.2	1.8	2.3	1.2	ENSDARP00000065236 : ENSDARP00000065236 pep:novel chromosome:ZFISH5:21:32828315:32869032:-1 gene:ENSDARG00000044419 transcript:ENSDART00000065237
CR523148	1.0	0.9	1.5	1.1	Gallus gallus finished cDNA, clone ChEST1033h5
CR523171	0.9	1.7	2.2	2.6	PREDICTED: similar to receptor-interacting protein 2
CR523188	0.9	0.8	0.9	0.3	Calcium regulated heat stable protein 1, 24kDa (CARHSP1)
CR523212	1.6	0.9	0.9	0.5	Essential meiotic endonuclease 1 homolog 1 (S. pombe) (EME1)
CR523262	1.8	1.8	2.2	1.4	Gallus gallus finished cDNA, clone ChEST970b1
CR523285	1.0	0.9	1.1	0.4	Gallus gallus finished cDNA, clone ChEST613j16
CR523421	0.7	0.7	0.7	0.2	Gallus gallus finished cDNA, clone ChEST293j18
CR523505	0.7	0.9	0.8	2.4	Gallus gallus finished cDNA, clone ChEST301m18
CR523537	0.7	0.9	0.5	1.4	Gallus gallus finished cDNA, clone ChEST874n7

CR523604	1.2	2.2	1.8	1.6	Gallus gallus finished cDNA, clone ChEST331j10
CR523685	0.7	0.8	0.6	0.2	Gallus gallus finished cDNA, clone ChEST836k22
CR523746	1.0	2.3	2.8	0.7	Transmembrane protein 196 (TMEM196)
CR523786	1.3	0.8	0.9	0.4	Gallus gallus finished cDNA, clone ChEST559j8
CR524040	0.6	1.1	0.8	0.8	PREDICTED: similar to ZMYM6 protein
CR524099	0.8	1.6	2.7	3.0	Gallus gallus finished cDNA, clone ChEST262g11
CR524103	0.9	0.5	0.4	0.3	Uroplakin 3B (UPK3B)
CR524171	1.7	3.0	2.9	2.1	Gallus gallus finished cDNA, clone ChEST769m7
CR524261	1.1	1.0	1.7	0.9	PREDICTED: oxysterol-binding protein-like protein 5
CR524277	0.8	1.0	0.5	0.4	Gallus gallus finished cDNA, clone ChEST914j4
CR524314	0.7	1.0	1.1	2.5	Gallus gallus finished cDNA, clone ChEST819a3
CR524355	0.8	1.4	1.5	1.1	Gallus gallus finished cDNA, clone ChEST806n6
CR524462	1.4	2.0	2.7	6.1	Copine VIII (CPNE8)
CR732824	1.8	1.2	1.0	1.0	Gallus gallus finished cDNA, clone ChEST795j14
CR733143	1.1	2.1	2.0	1.3	Gallus gallus finished cDNA, clone ChEST699i17
CR733296	1.0	0.7	0.6	0.1	Lipase, endothelial (LIPG)
CV041732	1.6	0.6	0.7	0.6	PREDICTED: hypothetical protein LOC421799 (LOC421799)
CV859232	2.3	1.9	2.6	2.1	Gallus gallus cDNA 5-, mRNA sequence; Embryonic gonad cDNA Library
CV862000	0.5	0.3	0.3	0.3	Transmembrane protein (Fragment)
CV892859	1.0	0.8	1.3	1.0	PREDICTED: similar to NADH2 dehydrogenase (ubiquinone) chain 1 - western lowland gorilla mitochondrion [Homo sapiens]
D00844	0.9	1.5	1.8	2.4	Coagulation factor X (F10) (Virus activating protease; VAP)
D16187	1.1	0.6	0.8	0.6	V-maf musculoaponeurotic fibrosarcoma oncogene homolog K (MAFK)
D26311	0.8	0.9	0.5	0.3	Dickkopf homolog 3 (Xenopus laevis) (DKK3)
D38026	1.1	1.2	1.5	0.6	Calpain 2, (m/II) large subunit (CAPN2)
D82364	1.0	1.7	1.0	1.9	TSC22 domain family, member 1 (TSC22D1)
DN928500	0.6	0.7	0.4	0.2	Microfibrillar associated protein 5 (MFAP5)
DR410768.1	2.5	0.9	1.1	1.1	Gallus gallus cDNA 5 , mRNA sequence; Embryonic gonadal PGC cDNA Library
DR425015	0.8	1.5	2.8	2.2	Gallus gallus cDNA clone naw13f10 5-, mRNA sequence; Chicken eye (hatched). Unnormalized (naw)
DR427916	1.0	1.0	1.5	0.6	Gallus gallus cDNA clone naw48c01 5-, mRNA sequence; Chicken eye (hatched). Unnormalized (naw)
DR428407	1.1	1.3	1.3	0.5	PREDICTED: similar to type I procollagen pro-alpha 2 chain, partial
DR429619	0.5	1.3	1.4	0.4	Gallus gallus cDNA clone nax19g10 5-, mRNA sequence; Chicken eye (embryo). Unnormalized (nax)
DR430769	2.1	0.9	0.8	0.5	PREDICTED: similar to Kinesin-like protein KIF6
DR431104	1.2	1.7	1.9	0.3	Gallus gallus cDNA clone nax39a04 5-, mRNA sequence; Chicken eye (embryo). Unnormalized (nax)
DT658523.1	1.1	1.0	1.5	0.8	Phospholipase C, beta 1 (phosphoinositide-specific) (PLCB1)
ENSGALT00 000001790.2	0.9	0.9	0.5	0.3	Gallus gallus finished cDNA, clone ChEST874n7
ENSGALT00 000012064.2	0.9	1.3	1.7	1.3	Gallus gallus finished cDNA, clone ChEST874n7
L06125	0.8	0.7	0.8	2.3	Heat shock transcription factor 2 (HSF2)
L34553	2.7	4.4	4.8	6.0	Chemokine (C-C motif) ligand 4 (CCL4)
L34554	0.8	1.2	1.7	1.0	Lymphocyte antigen 6 complex, locus E (LY6E) (Stem cell antigen 2) (SCA-2)
M15889	0.5	0.7	0.3	0.1	Elastin (supravalvular aortic stenosis, Williams-Beuren syndrome) (ELN)
M16199	3.7	22.4	26.8	18.5	Interleukin 8 (IL8)

M27260	1.1	1.3	0.9	2.0	Heat shock 70kDa protein 5 (glucose-regulated protein, 78kDa) (HSPA5)
M29076	0.7	1.9	1.8	0.8	Glutamate-ammonia ligase (glutamine synthetase) (GLUL)
M31764	1.2	0.7	0.6	1.0	Phosphoribosylaminoimidazole carboxylase, phosphoribosylaminoimidazole succinocarboxamide synthetase (PAICS)
M37785	1.3	1.2	2.1	1.2	Solute carrier family 2 (facilitated glucose transporter), member 3 (SLC2A3)
M61145	0.8	1.4	1.7	1.5	Prion protein (p27-30) (Creutzfeldt-Jakob disease, Gerstmann-Strausler-Scheinker syndrome, fatal familial insomnia) (PRNP)
M61754	0.9	1.3	0.9	0.5	Midkine (neurite growth-promoting factor 2) (MDK)
M64990	0.8	2.6	3.4	2.1	Prostaglandin-endoperoxide synthase 2 (prostaglandin G/H synthase and cyclooxygenase) (PTGS2)
M74544	1.1	2.5	3.3	1.5	NF-kappaB inhibitor alpha (I-kappa-B-alpha)
M80584	1.2	1.5	1.2	0.4	Lumican (LUM)
M83235	2.5	0.8	1.0	2.6	High-mobility group box 2 (HMGB2)
M87294	0.7	1.3	1.5	1.7	Neuropeptide Y (NPY)
S59426	1.5	0.8	0.8	0.5	DNA repair protein RAD51 homolog (RecA homolog, E. coli) (S. cerevisiae) (RAD51)
S78477	1.6	2.6	3.5	1.0	Glucagon (GCG)
TC226216	1.7	0.8	0.7	0.4	Disheveled associated activator of morphogenesis 2 (DAM2_Human), partial (28%)
TC226319	0.9	1.3	0.8	0.5	MGC83749 protein, partial (7%)
TC226515	1.7	1.0	1.5	1.0	BchX (Fragment), partial (10%)
TC226615	0.9	1.7	1.6	1.3	Unknown
TC227057	0.8	0.7	0.5	0.8	Q6IGH5 (Q6IGH5) HDC06258, partial (9%) [TC227057]
TC227149	0.6	0.6	0.5	0.2	Unknown
TC227370	1.1	0.8	1.4	1.1	Unknown
U00147	1.0	0.7	1.1	0.5	Filamin B, beta (actin binding protein 278) (FLNB)
U01047	1.6	0.8	0.9	0.7	RAD52 homolog (S. cerevisiae) (RAD52)
U09350	2.0	0.2	0.3	0.2	Vasoactive intestinal peptide (VIP)
U12438	1.9	0.8	1.0	0.3	Replication factor C (activator 1) 2, 40kDa (RFC2)
U18309	2.0	0.5	0.8	0.5	Kinesin family member 4A (KIF4A)
U30520	1.1	1.2	1.1	0.4	Chromosome 5 open reading frame 13 (C5orf13)
U34977	0.8	1.0	0.7	0.3	Fibromodulin (FMOD)
U37691	0.6	1.1	1.4	1.0	Cathepsin K precursor (JTAP-1)
U46504	1.2	1.8	1.8	1.6	Nitric oxide synthase 2, inducible (NOS2)
U62026	3.3	0.7	1.4	0.6	Centromere protein F, 350/400ka (mitosin) (CENPF)
U66463	0.6	1.2	0.9	1.0	Matrix metallopeptidase 16 (membrane-inserted) (MMP16)
V00428	1.6	4.4	5.5	3.1	Lysozyme (renal amyloidosis) (LYZ)
X02009	0.7	2.7	3.6	3.2	Ovotransferrin precursor (Conalbumin); Lactotransferrin (LTF)
X02827	0.8	0.9	1.2	0.4	Aminolevulinate, delta-, synthase 1 (ALAS1)
X03509	0.9	4.7	5.5	2.0	Creatine kinase, brain (CKB)
X05343	0.7	1.9	3.4	1.7	Mature avidin (LOC396260)
X16021	0.9	1.9	1.8	1.0	N-acetyltransferase 1 (arylamine N-acetyltransferase) (NAT1)
X16881	1.9	0.4	0.7	0.4	Cell division cycle 2, G1 to S and G2 to M (CDC2)
X17480	0.6	1.8	1.6	0.6	N-acetyltransferase 2 (arylamine N-acetyltransferase) (NAT2)
X51485	0.7	0.6	0.5	0.3	Nuclear factor I/B (NFIB)
X59284	0.4	1.4	1.3	0.7	Nephroblastoma overexpressed gene (NOV)
X61198	1.4	1.0	1.5	0.8	Lysozyme G-like 2 (LYG2)

X62531	1.8	0.4	0.7	0.8	G2/mitotic-specific Cyclin B2 (CCNB2)
X65459	1.1	0.3	0.8	0.2	Fatty acid binding protein 7, brain (FABP7)
X77960	1.4	1.5	2.1	1.1	Retinol binding protein 4, plasma (RBP4)
X80503	0.9	1.2	1.8	0.9	ST3 beta-galactoside alpha-2,3-sialyltransferase 1 (ST3GAL1)
X80792	2.0	0.6	1.0	0.6	Structural maintenance of chromosomes 2 (SMC2)
X91638	1.9	0.9	1.0	0.9	SWI/SNF related, matrix associated, actin dependent regulator of chromatin, subfamily a, member 2 (SMARCA2)
Y00416	1.8	0.7	0.9	1.1	High-mobility group nucleosomal binding domain 2 (HMGN2)
Y09235	1.1	1.8	3.2	1.1	Glutaredoxin (thioltransferase) (GLRX)
Y11273	0.5	1.7	2.0	2.0	Deiodinase, iodothyronine, type III (DIO3)
Y12225	1.0	1.7	2.2	1.3	Spleen focus forming virus (SFFV) proviral integration oncogene spi1 (SPI1)
Y12601	1.0	1.2	1.8	3.2	Adenosine A2b receptor (ADORA2B)
Y13903	0.6	0.5	0.7	0.3	Matrix Gla-protein precursor (MGP)
Y14971	3.7	17.3	19.7	11.3	K60 protein precursor (CXC chemokine K60)
Y15006	4.3	8.4	10.8	4.1	Interleukin-1, beta (IL1B)
Y17968	1.9	0.8	1.0	1.2	High-mobility group box 1 (HMGB1)
Y18692	1.3	3.7	4.4	3.2	Chemokine K203 precursor; Chemokine (C-C motif) ligand 4 (CCL4)
Z14957	2.0	1.6	4.4	2.5	Carbonic anhydrase II (Carbonate dehydratase II) (CA-II)
Z19110	0.6	0.8	0.4	0.3	Chick Ephrin type-B receptor 1 (EPHB1) (Tyrosine-protein kinase receptor EPH-2) (Tyrosine kinase CEK6 receptor)
Z21536	0.8	2.2	2.6	2.1	Fibropellin III (Fragment)
Z21646	0.5	0.9	1.0	0.7	CCAAT/enhancer binding protein (C/EBP), beta (CEBPB)
Z48921	0.9	1.1	1.6	1.6	Beta-2 microglobulin
