# Supplementary material for: Transcriptional profiling of host gene expression in chicken embryo lung cells infected with laryngotracheitis virus
Source: BMC Genomics. 2010 Jul 21;11:445. doi: 10.1186/1471-2164-11-445 (PMC3091642; doi:10.1186/1471-2164-11-445)
Supplement: Additional file 2 — GenBank accession numbers of 7 clusters for expression patterns. [file 1471-2164-11-445-S2.PDF]

Cluster	Genes
1	CR407399, BX935001, CR353415, AF505881, BX935349, DR429619, BX935550, CR406752, BU229919, BX935133, X17480, BU270966, BM485850, CR407116, CR386282, BU475842, M29076, X05343, BX931154, BI392244, AF119370, BX930367, CR391749, CR387485, BX935204.2, BU304608, CR386009, BU366414, BU441936, BU295027, CR354135, BU235342, AF070478, BX931940, X02009, AJ720414, BU350029, CR389889, BX934150, CR389094, BU440826, CR524355, CR385332, BU305390, BU306465, BU471244, BX935011, BU351880, Z21536, AF327880, CR524099, L34554, CR354333, BU374834, X02827, AY450642, M61145, CR354361, BU135143, CR389281, AJ585767, CR386878, DR425015, AF537108, BU120686, AJ719555, BU271039, CR390976, CR406066, BX935069, BU353859, BX273853, CR407528, CR352752, BX931404, BU434877, CN210627.1, CR390810, M64990, BX934073, BU291905, X80503, BU278451, AJ851395, BX933888, AF139097, X16021 ,AJ720428, BU353622, BX950745, BX930381, CR386459, BU261103, BU463345, AJ720823, BU421389, BX935573, AY434090, BU280688, AJ720988, AJ719947, CR385698, Z48921, ENSGALT00000012064.2, D00844, BX935985, BX931790, CR386526, AF031168, CR353526, CR353501, AB105812, CR387033, BU434803, TC226615, CR406411, CR387287, CR389027, AF285876, AJ719326, AJ851647, AF472618, AJ720958, AJ720683, BU242783, CR353484, CR406359, CR523171, CR407328, BX934532, CK609854, BX930456, BX935591, CR407493, BX934666, BX931291, AJ720018, BU447021, CK609691, CR406335, DR427916, BU350156, BU269038, CD729265, CR523746, AJ720905, BU200000, CR523148, CD728822, CR354286, BU307877, BU124346, CR406056, CV892859, BU114438, CR390935, BU398229, AW239595, BU418843, CR523285, CR387838, CR390179, BX931191, AJ450520.1, BU373433, AJ851390, AF261079, U00147, BU339355, BX935378, BU476209, AJ851520, BU420127, AJ851803, BU441594, Y12225, BX932426, BU456708, BU363918, DR428407, TC227370, CR389443, AJ851505, BX929698, AB101005, D38026, CR406822, BU281908, DT658523.1, BX950642, BX929599, BU425020, BU274538, CR524261, BU269552, CR382434, AJ720825, AF051399, CR390337, BU122751, AJ719903, BU221355, BU217629, BU319109, Y09235, BU457236, CR389517, CR733143, BX929804, AJ851669, BU359249, BX950657, BU241296, AJ720017, AJ720705, AF075708, M74544, AJ851569, CR386957,CR523604, BX933725, CR523095, BU111493, BX932427, CK610423, CR387701, CR353337, BU444777, DR431104, U46504, BU405306, AJ851808, BX929282, BX930147, CR386034, BU120465, CR389767, BX935863, AJ721113, AJ720504, BU262875, CK612370, AJ720739, BU477418, BX931532, BU111648, AJ851748, BU404404, M37785, BX929654.1, AJ720638, Y18692, BU307848, CR387256, CR522990, AB031025, BX930046, BU356156, CR406894, AB100407, CR406552, BU375972, CR524462, BU347615, BX950502, X77960, CR405944, CR386411, BU420182, BU320805, AJ719869, BU253190, AJ719362, AF153205, S78477, BX934061, BX931971, BU280180, V00428, BU382851, BU115553, CR524171, BU143074, Z14957, CR352822, CR385495, L34553
2	BU222772, BX934937, BU239064, BX930055, CR385166, BU234625, BU307434, U66463, BX935496, BX931663, BU145104.1, BX266231, CR386133, CR387448, CR523537, BU411355, BU405648, BU452127, CR523505, BU277690, BX935026, AJ720523, BX935484, CR388986, BX931007, BU313670, BX931418, BU323058, AB154518, L06125, BX932547, BX933994, CR382435, CO760996, BX931246, CR352395, CO635775, CR388971, BX933437, BU284059.1, CR406246, BU377399, CR406496, CR389140, BX261173, AJ004940, CR388639, AJ720657, BU362756, BU440496, BX930261, BU449643, BX935864, AJ720344, BX930047, CR523007, CR390654, CR406447.1, BU422376, CR386845, CR352420, BX950711.1, CR387745, BU291832, CN229430, AJ720793, CR390841, CR406543, D82364, BU336892, CR389813, BU379145, BU343906, CR386489, M27260, CR407473, BX930115, AJ720471, CR338704, AJ719335, BU413519, BU338914, AJ720331, CN218923.1, AB031398, CR390562, CR406252, BU321464, AJ719454, BU120054, AJ719858, CR390519, BU287966, AJ719296, AJ720196, AF498103, AJ720303
3	BU286389, M15889, BU376215, CR405963, AF125575, AY265159, BX930215, AL584098, CR385494, CV862000, BX936224, CR388879, TC227149, BX935571, CR524040, DN928500, CO506634, BX932293, BU470096, Z19110, CF251376, BU217918, CR389475, BX930311, CR389509, BU118929, CR391580, BU380570, CR391404, BU212825, BX271230, BX929845, CB270855.1, X51485, CR407207, BX950437, AF239837, BU394402, BU259459, CR523685, CR523421, BU202662, CR386318, BU326858, AY574987, CR388632, BX930091, CR390162, CR391426, CR524277, BX276973, BU199991, D26311, BX929886, TC227057, CR385787, CO505470, U34977, BU269342, BU340649, AF289218.1, CR524103, BU395153, CR406404, CR391470, ENSGALT00000001790.2, CR390208, TC226319, M61754, BU368845, BX935060, BU465112, BU278251, BU322109, BX931577, CR388945, BU390859, BU437856, BU417507, BU448303, CR385622, AJ719751, CR387420, BX933946, BX931297, BU229963, M31764, BU475739, BU279469, BU121809
4	Y14971, M16199, Y15006

5	CR391100, AJ721110, BU420694, CR385201, AF062392, X03509, AJ309540, AF432506, AJ851480
6	X59284, AJ720586, BX930231, BX930357, BU449222, CR387505, AJ307060, AJ721104, BU467932, CR387087, BX262827, BX936276, AF096264, CD764582, CR406806, CR389704, CR386923, Z21646, BX272499, AF098515, BU352242, CR389612, Y11273, BX933595, CR385142, Y13903, CR406836, AJ720351, BM440220.1, AJ719438, BX933582, AJ720298, CR385831, BU144940, AY278202, U37691, CF250950, BX268699, BU123182, CR386594, CR354132, AJ851633, BX932445, AJ851506, AJ442179, BU205290, AJ851578, CN223734, CR390375, BU456005, AF020315, BX933315, AJ393786, AJ719321, CR386687, AJ720179, M87294, CR385948, CR406322, BU317012, CR524314, BU433279, BU353686, BX931352, CR387244.1, AJ720845, BU442255, BU472175, BU391712, AF131224, AJ720717, AJ721107, AB045597, CN219930, BU242338, BX934121, AJ719902, BX933454, AJ721124, CR406511, Y12601, CR385852, BX267574, CR385678, AJ851432
7	CR523188, BU138507, CR733296, BU339047, CR406175, BX262539, AJ851548.1, BX272837, CR387914, U30520, CR407225, BU440951, AJ719718, CD763113, X65459, AJ720043, BU352215, D16187, CR385514, CR338819, BU281664, BU279953, BX933739, BX950656, M80584, CR389058, BU325184, AJ719507, BX932384, BU278528, BX266352, BU217968, CR387761, BU218715, CR385124, CR385491, AJ720861, AJ720948, AJ719676, AB055783, BX935595, BX933041, BU221762, BU284657, CR385841, CR523786, BU246669, BX261359, BU111203, BX277390, CR387450, BU223203, BX930754, AJ720030, CR385747, X61198, CR354360, BX279056, AJ719817, AJ721080, CF254917, BX935712, CR390951, BU259849, BU306841, BU338496, S59426, AJ720339, CR391234, AJ393939, AJ719295, BU300550, BU483476, BU385227, AY245433, CR354068, AJ720813, CR354334, BU258106, BU351543, BU481905, CV041732, BU353919, CR522951, CR406946, BU483257, BX932834, BX933015, CR523212, BU263588, AJ720074, U01047, AJ719443, AJ851370, CR354151, AJ720066, BU386549, CR352895, TC226216, CR390178, BU395620, CK609466, AJ720011, AJ719973, CR385559, AJ720366, AJ719818, BX934024, BU317358, AJ720016, AJ720138, CR353524, BU273218, AJ719984, TC226515, AF411083, AJ851680, AB007445, CR406810, BX262245, CR732824, AF000241, CR387903, AJ719352, AJ719854, CR385367, BX930996, AJ720217, BX935110, BU453230, AJ720410, BU334887, BU253133, AF308592, CR523262, X62531, CR386671, BU426499, Y00416, BU118728, AJ719475, CR386703, X16881, Y17968, CR385527, BU456021, AJ720929, BU433762, BX929945, BU305545, AJ719627, BX950396, CR385721, X91638, BU260738, BU333516, U12438, CR389319, BX933127, BX950762, AJ851705, BU266634, AJ719593, U18309, BX932979, U09350, X80792, CR406802 ,CR407416, CR390609, CR387035, BU255739, DR430769, CR385581, BU391226, CR406298, CR390858, AJ443395, CR406132, BX934082, BX931288, BX932212, AF459286, CR406603, CR523033, CR385186, AJ719535, BU350636, AY237249, CR389935, AJ719282, AJ735439, CV859232, CR352660, BX936026, CR406622, AJ720555, AJ719348, DR410768.1, BU409770, AF068831, BU468099, M83235, BU325823, BU106686, CR352647, BU456843, BX932207, AY040527, AF053401, AJ719387, U62026, CR387407, AF534111, AJ719339, BX933215, BI067703, BU409199
