# Supplementary material for: Transcriptional profiling of host gene expression in chicken embryo lung cells infected with laryngotracheitis virus
Source: BMC Genomics. 2010 Jul 21;11:445. doi: 10.1186/1471-2164-11-445 (PMC3091642; doi:10.1186/1471-2164-11-445)
Supplement: Additional file 3 — Gene Ontology generated by IPA. The 789 differentially expressed genes were divided into 65 groups based on their functions. [file 1471-2164-11-445-S3.PDF]

**Functions**	**Number of gene**
Cancer	140
Genetic Disorder	125
Cellular Growth and Proliferation	106
Cell Death	95
Reproductive System Disease	71
Neurological Disease	71
Cellular Movement	68
Gastrointestinal Disease	62
Small Molecule Biochemistry	61
Cell-To-Cell Signaling and Interaction	60
Cell Cycle	57
Hematological System Development and Function	56
Inflammatory Disease	55
DNA Replication, Recombination, and Repair	55
Tissune Development	55
Inflammatory Response	54
Cellular Development	52
Antigen Presentation	51
Cell-Mediated Immune Response	51
Humoral Immune Response	51
Molecular Transport	50
Lipid Metabolism	45
Immunological Disease	45
Tissue Morphology	45
Gene Expression	44
Metabolic Disease	43
Skeletal and Muscular Disorders	42
Hematological Disease	40
Immune Cell Trafficking	39
Connective Tissue Disorders	38
Cardiobascular Disease	36
Respiratory Disease	35
Organismal Survival	35

Cardiovascular System Development and Function	34
Endocrine System Disorders	31
Hematopoiesis	31
Cell Signaling	28
Skeletal and Muscular System Development and Function	27
Dermatological Diseases and Conditions	27
Connective Tissue Developmemt and Function	26
Organismal Injury and Abnormalities	24
Drug Metabolism	21
Vitamin and Mineral Metabolism	21
Cellular Assembly and Organization	19
Carbohydrate Metabolism	18
Cell Morphology	18
Cellular Function and Maintenance	17
Hepatic System Disease	17
Embryonic Development	14
Nervous System Development and Function	13
Cellular Compromise	13
Organismal Functions	12
Infectious Disease	12
Free Radical Scavenging	11
Amino Acid Metabolism	9
Behavior	9
Organismal Developmemt	8
Nutritional Disease	6
Infection Mechanism	6
Organ Morphology	5
Tumor Morphology	4
Endocrine System Development and Function	3
Reproductive System Development and Function	3
Digestive System Development and Function	3
Renal and Urological System Development and Function	2
