# Supplementary material for: Transcriptional profiling of host gene expression in chicken embryo lung cells infected with laryngotracheitis virus
Source: BMC Genomics. 2010 Jul 21;11:445. doi: 10.1186/1471-2164-11-445 (PMC3091642; doi:10.1186/1471-2164-11-445)
Supplement: Additional file 4 — Gene lists of each network. Gene symbols and GenBank accession numbers were displayed for the illustrations of network analysis. Only focus molecules, which were elected as differentially expressed genes from microarray analysis, are marked as bold and GenBank accession numbers are provided. Accession numbers for reference molecules were not included in the table. [file 1471-2164-11-445-S4.PDF]

Network #1		Network #2		Network #3	
**Symbol**	**GenBank**	**Symbol**	**GenBank**	**Symbol**	**GenBank**
**BUB1**	**AJ719676**	**ACLY**	**AJ851548.1**	**CD44**	**AF153205**
**BUB1B**	**AY245433**	Akt		Collagen(s)	
**CCNB2**	**X62531**	**APP**	**AF289218.1**	**DIO2**	**AF125575**
**CDC2**	**X16881**	**AQP9**	**BX930047**	**DIO3**	**Y11273**
**CDC20**	**AJ720948**	Caspase		**DUSP6**	**AY278202**
**CENPF**	**U62026**	DNAJ		ERK	
Cyclin A		**DNAJA1**	**AJ720331**	**FBLN1**	**AF051399**
Cyclin B		**DNAJB6**	**AJ851505**	**FGF7**	**CR354361**
Cyclin E		**DNAJB9**	**AJ720657**	Fibrin	
E2f		**FANCC**	**AJ851680**	**HBEGF**	**AF131224**
**ETV4**	**AF075708**	**FBLN5**	**BX935571**	Laminin	
**GMNN**	**AJ720138**	**FLNB**	**U00147**	**MCM6**	**AJ720016**
Histone h3		**GLUL**	**M29076**	**MDK**	**M61754**
Histone h4		Gpcr		Mmp	
**HPGD**	**BX934937**	Gsk3		**MMP16**	**U66463**
**IL6**	**AJ309540**	**HMGB2**	**M83235**	**MMP27**	**AF062392**
**JAK**	**AF096264**	**HNRNPD**	**AJ720813**	**MMP7**	**AJ719326**
**MAD2L1**	**BX935595**	**HSF2**	**L06125**	**NID1**	**AF239837**
MAP2K1/2		Hsp22		**NOV**	**X59284**
**MCM2**	**AJ719593**	Hsp27		p70 S6k	
**MCM3**	**AJ719352**	Hsp70		Pdgf	
**MCM5**	**AJ720074**	Hsp90		PDGF BB	
**NASP**	**AJ719339**	**HSPA5**	**M27260**	PDGF-AA	
**NUSAP1**	**AJ720339**	**HSPA8**	**AJ004940**	**PDGFB**	**AB031025**
**PBK**	**BX934024**	**ITGB5**	**AY434090**	PLC gamma	
**PTTG1**	**BX932212**	**MAT1A**	**BX935026**	**RASD1**	**BX930456**
**RAD51**	**S59426**	Nos		**RASGRP3**	**AJ720845**
**RAD52**	**U01047**	**PHLDA2**	**BX950502**	**SERPINB10**	**AF053401**
Rb		PP2A		**SLA**	**AJ721107**
RPA		**PPT1**	**AJ720351**	**TFPI2**	**BX934121**
**SNX10**	**AJ720825**	**PRNP**	**M61145**	Tgf beta	
SOCS		Proteasome		**TRIM63**	**BX931246**
**TOP2A**	**AB007445**	**RFK**	**AJ719362**	Trypsin	
**TYMS**	**BX932834**	**SMARCA5**	**AJ719817**	VAV	
**WARS**	**BX933994**	**WISP1**	**BX934666**	**VAV3**	**AJ851569**

Network #4		Network #5		Network #6	
**Symbol**	**GenBank**	**Symbol**	**GenBank**	**Symbol**	**GenBank**
**BPI**	**BX930367**	Adaptor		Ap1	
**CCL20**	**AB101005**	**ALAS1**	**X02827**	**B2M**	**Z48921**
**CCL4**	**Y18692**	**AP1S2**	**AJ720066**	**BATF3**	**BX932427**
**CD82**	**AJ719947**	**ASS1**	**CR407399**	**BNIP3**	**CR407493**
**CITED4**	**AF261079**	Cbp/p300		C1q	
**F10**	**D00844**	**CEBPB**	**Z21646**	Calcineurin	
**F2RL1**	**AJ851370**	**CSTB**	**BX931532**	**CD47**	**AJ719869**
**FABP4**	**AF432506**	Cyclooxyge		**CKB**	**X03509**
HLA-DR		**ELOVL6**	**AJ720366**	**CLU**	**AF119370**
**HMGB1**	**Y17968**	FABP		Cpla2	
IFN Beta		**FABP7**	**X65459**	Creb	
Ifn gamma		GC-GCR		**DUSP1**	**CR387287**
Ikb		**GEM**	**AB105812**	**EPS15**	**AJ719296**
IKK		**IL1B**	**Y15006**	ERK1/2	
IL12		**IRG1**	**AJ720739**	**FMOD**	**U34977**
**INSIG1**	**AJ719295**	JINK1/2		**HNRNPM**	**AJ719854**
LDL		**MGP**	**Y13903**	**ID1**	**AY040527**
**LSP1**	**AJ851520**	N-cor		Ige	
NF-kappaB		**NFIB**	**X51485**	IgG	
NFkB		NF- 1		IL1	
NfkB-RelA		**PCDH7**	**CR388971**	Interferon	
NfkB1-RelA		**PDE4B**	**AJ720018**	**LTF**	**X02009**
**NFKBIZ**	**AJ721113**	PEPCK		**LYZ**	**V00428**
**NOS2**	**U46504**	**PMP2**	**BX935863**	**MAFK**	**D16187**
**RHOB**	**AF098515**	**PPARG**	**AB045597**	Mek	
SAA@		**PTGS2**	**M64990**	MHC -I	
**SERPINB6**	**AJ719818**	Rxr		MHC -II	
**SOCS1**	**BX933215**	Secretase		**NFKB1**	**AF000241**
Sod		**SEPP1**	**AJ719438**	NGF	
**SOD1**	**BX935573**	Smad		**NSFL1C**	**AJ720428**
**SOD3**	**BX929804**	**SMARCA2**	**X91638**	P38 MAPK	
**TFPI**	**CR353337**	STAT5a/b		**SLC2A3**	**M37785**
Tlr		SWI-SNF		TCR	
**TLR7**	**AJ720504**	TYR		**TRIB2**	**AJ720823**
**TRAF3IP2**	**CR390935**	**VNN1**	**AJ721110**	Ubiquitin	
