# Supplementary material for: Transcriptional profiling of host gene expression in chicken embryo lung cells infected with laryngotracheitis virus
Source: BMC Genomics. 2010 Jul 21;11:445. doi: 10.1186/1471-2164-11-445 (PMC3091642; doi:10.1186/1471-2164-11-445)
Supplement: Additional file 5 — Six gene networks. (A) network #1 (B) network #2 (C) network #3 (D) network #4 (E) network #5 and (F) network #6 are displayed. Enlarged images are followed by small alphabetical orders (a - d) to indicate dpi time points. Colored shapes indicate focus molecules, which were identified as differentially expressed genes by microarray analysis, while clear shapes indicate reference molecules. The green represents down-regulation and the red represents up-regulation. Degree of color intensities indicates levels of fold changes. [file 1471-2164-11-445-S5.PDF]

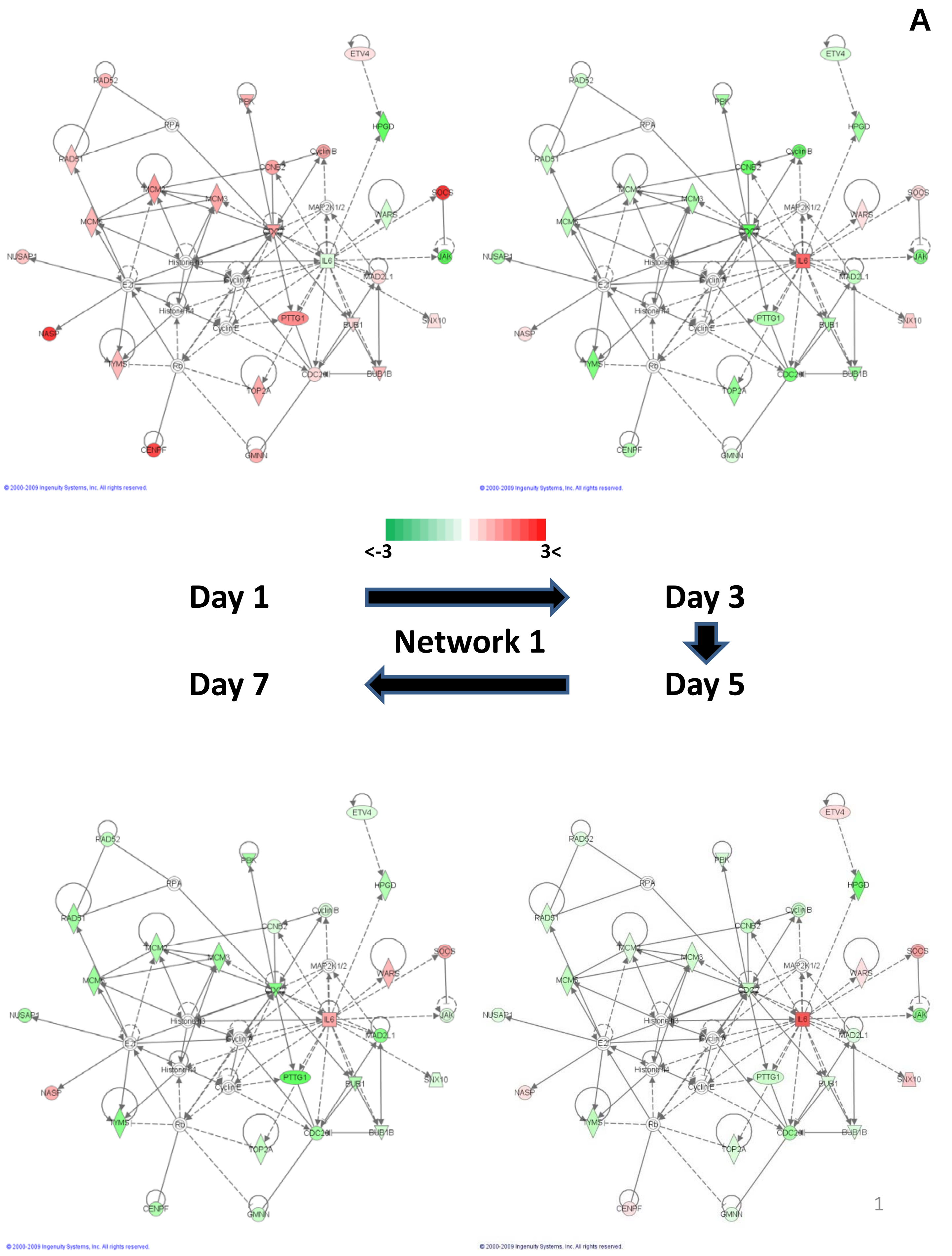
A
<-3
3<
Day 1
Day 3
Network 1
Day 5
Day 7
ETV4
RAD52
PBK
RPA
HPGD
RAD51
Cyclin B
CCNB2
MCM2
MCM3
SOCS
MAP2K1/2
WARS
MCM5
NUSAP1
Histone h3
IL6
JAK
E2f
Cyclin A
MAD2L1
Histone h4
PTTG1
SNX10
NASP
Cyclin E
BUB1
TYMS
Rb
CDC20
BUB1B
TOP2A
CENPF
GMNN
© 2000-2009 Ingenuity Systems, Inc. All rights reserved.

Network 1

A-a

Day 1

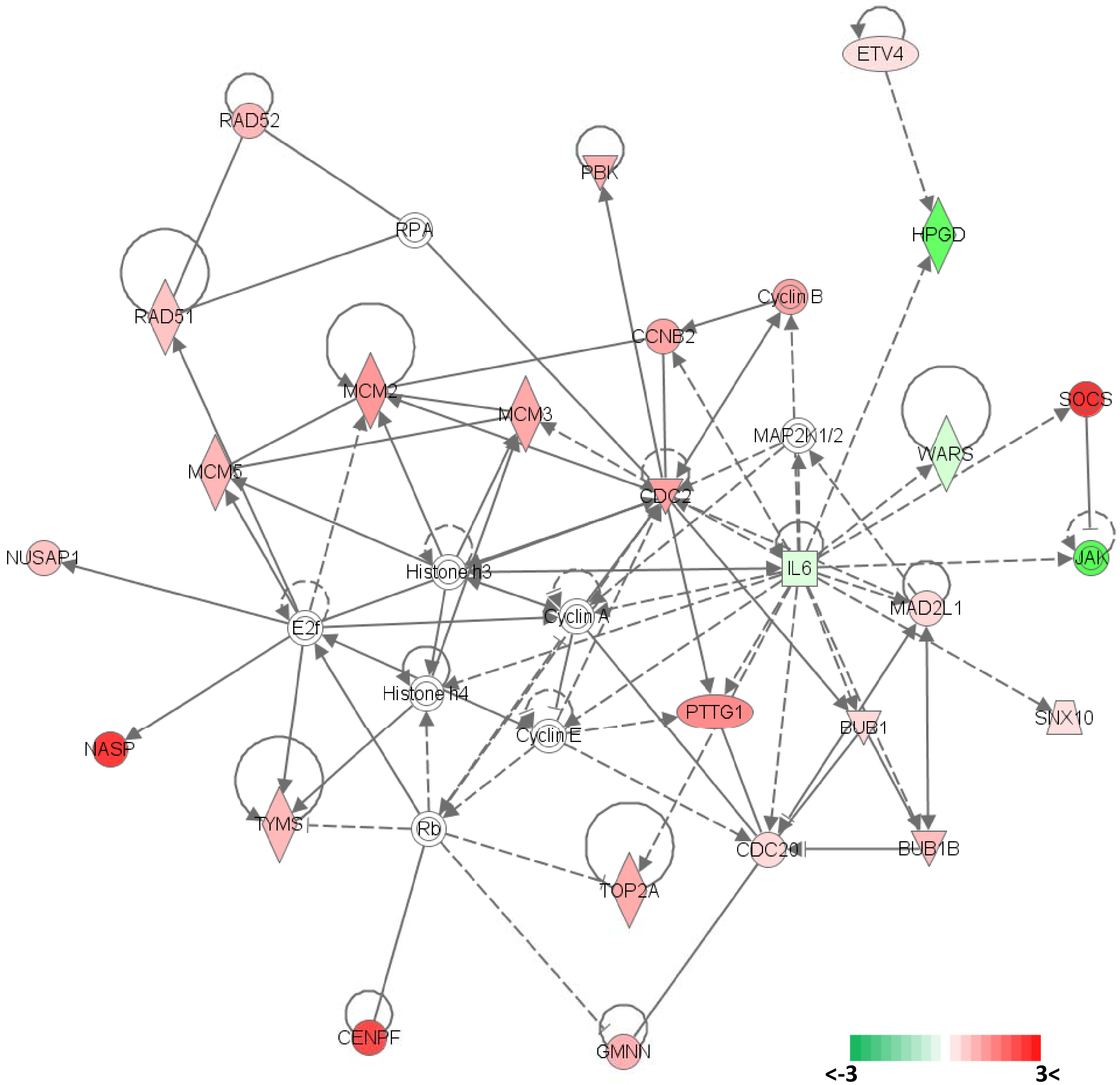

© 2000-2009 Ingenuity Systems, Inc. All rights reserved.

Network 1

A-b

Day 3

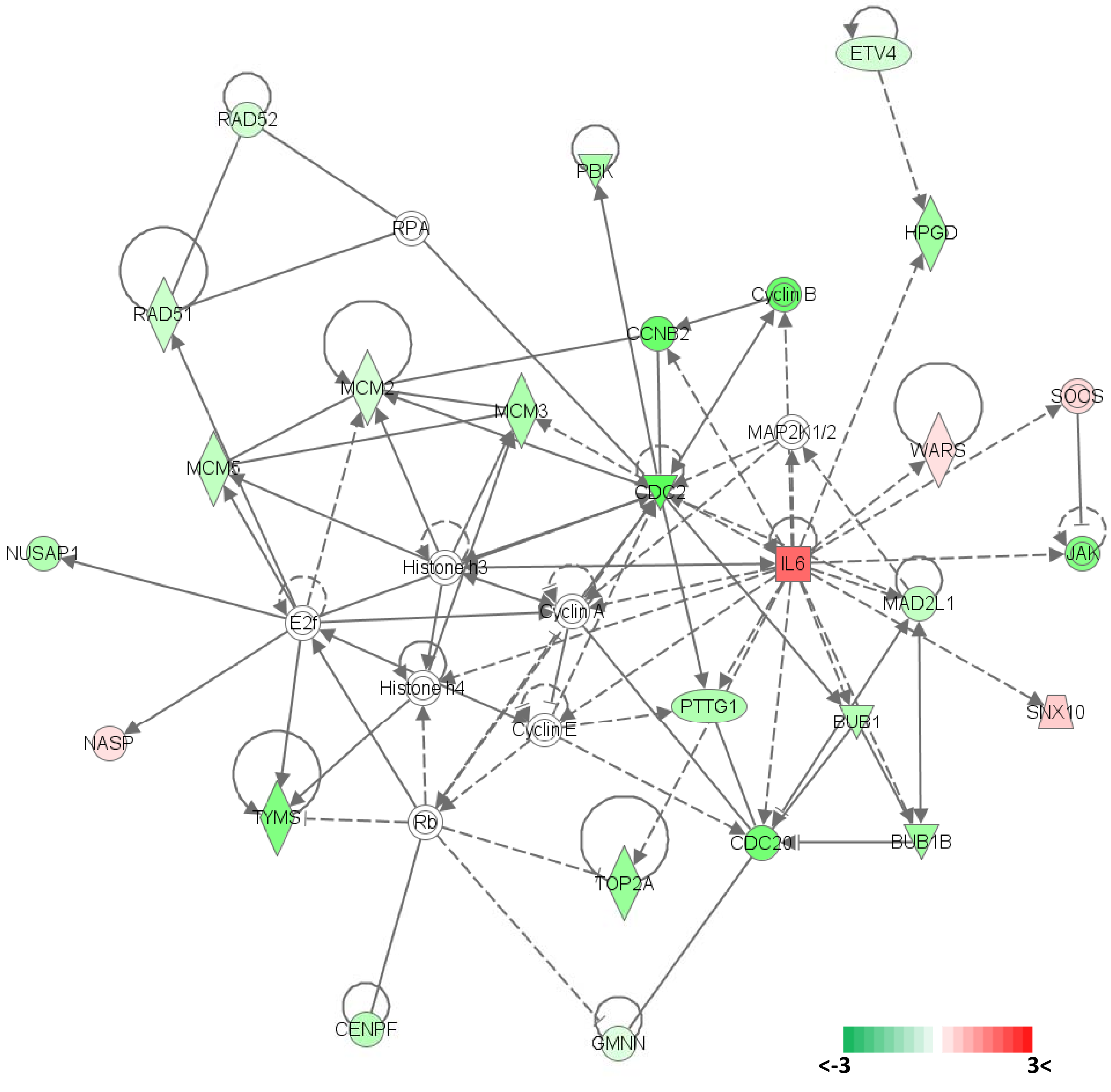

© 2000-2009 Ingenuity Systems, Inc. All rights reserved.

Network 1

A-c

Day 5

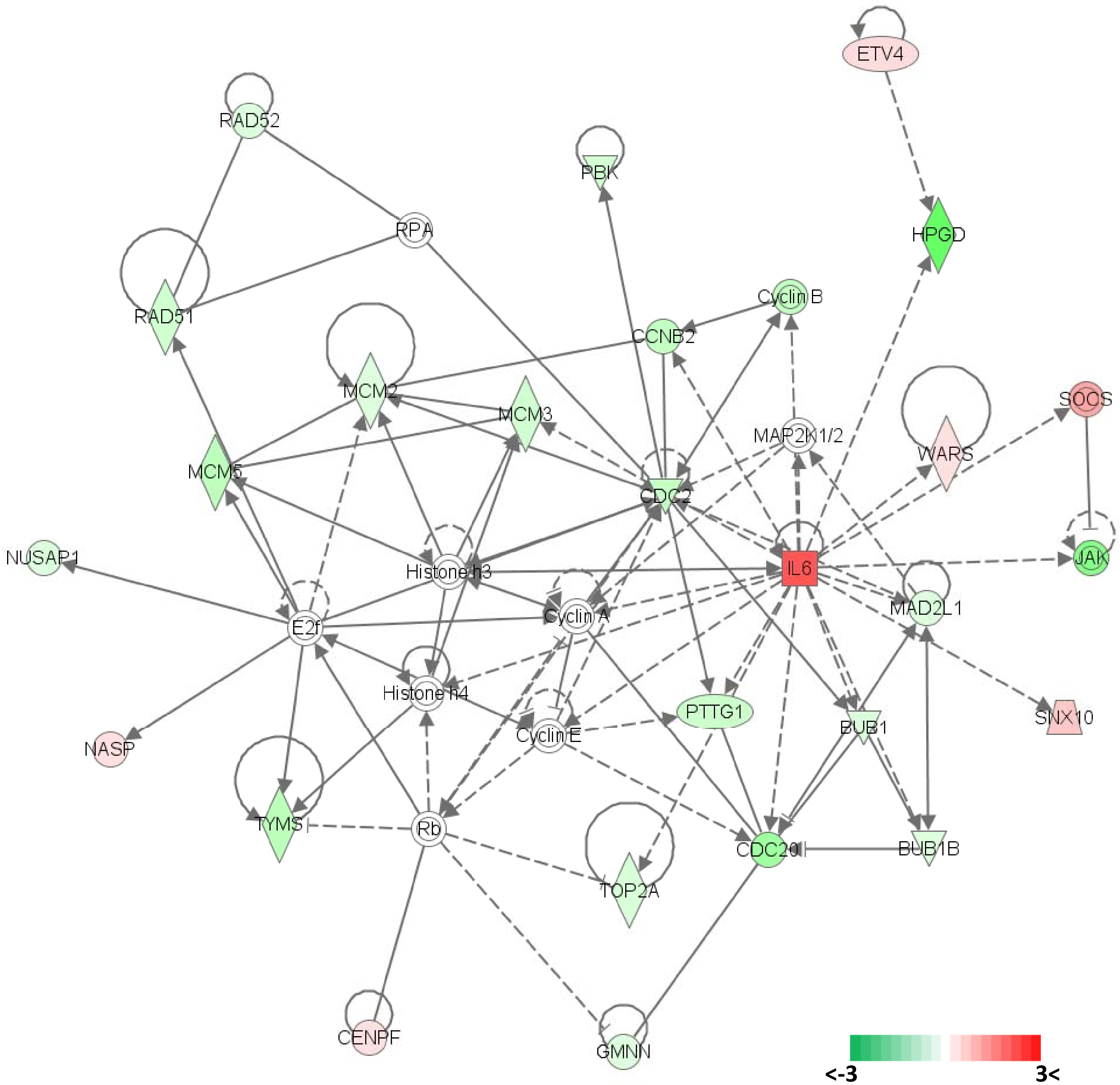

© 2000-2009 Ingenuity Systems, Inc. All rights reserved.

Network 1 A-d

Day 7

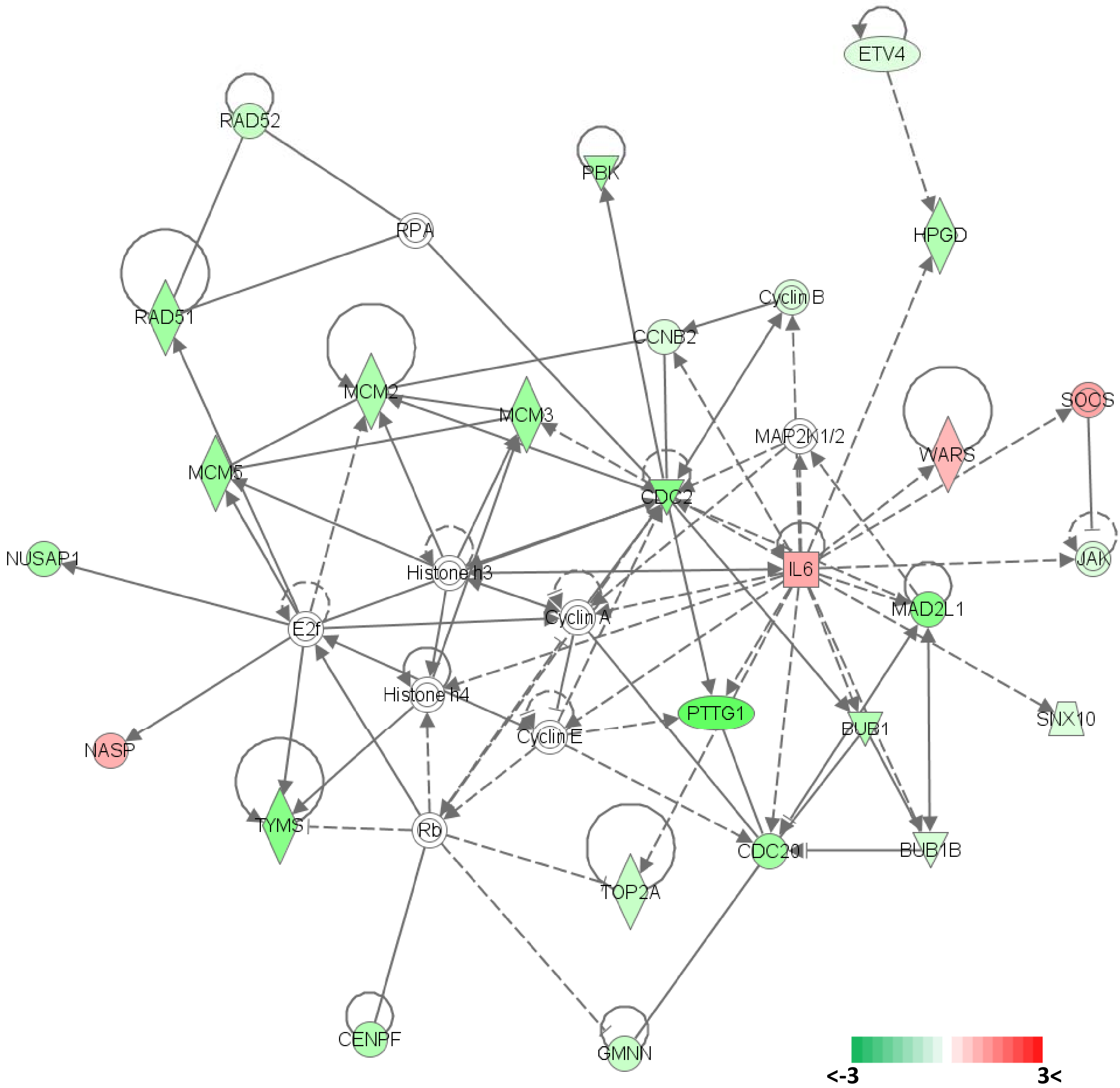

© 2000-2009 Ingenuity Systems, Inc. All rights reserved.

B

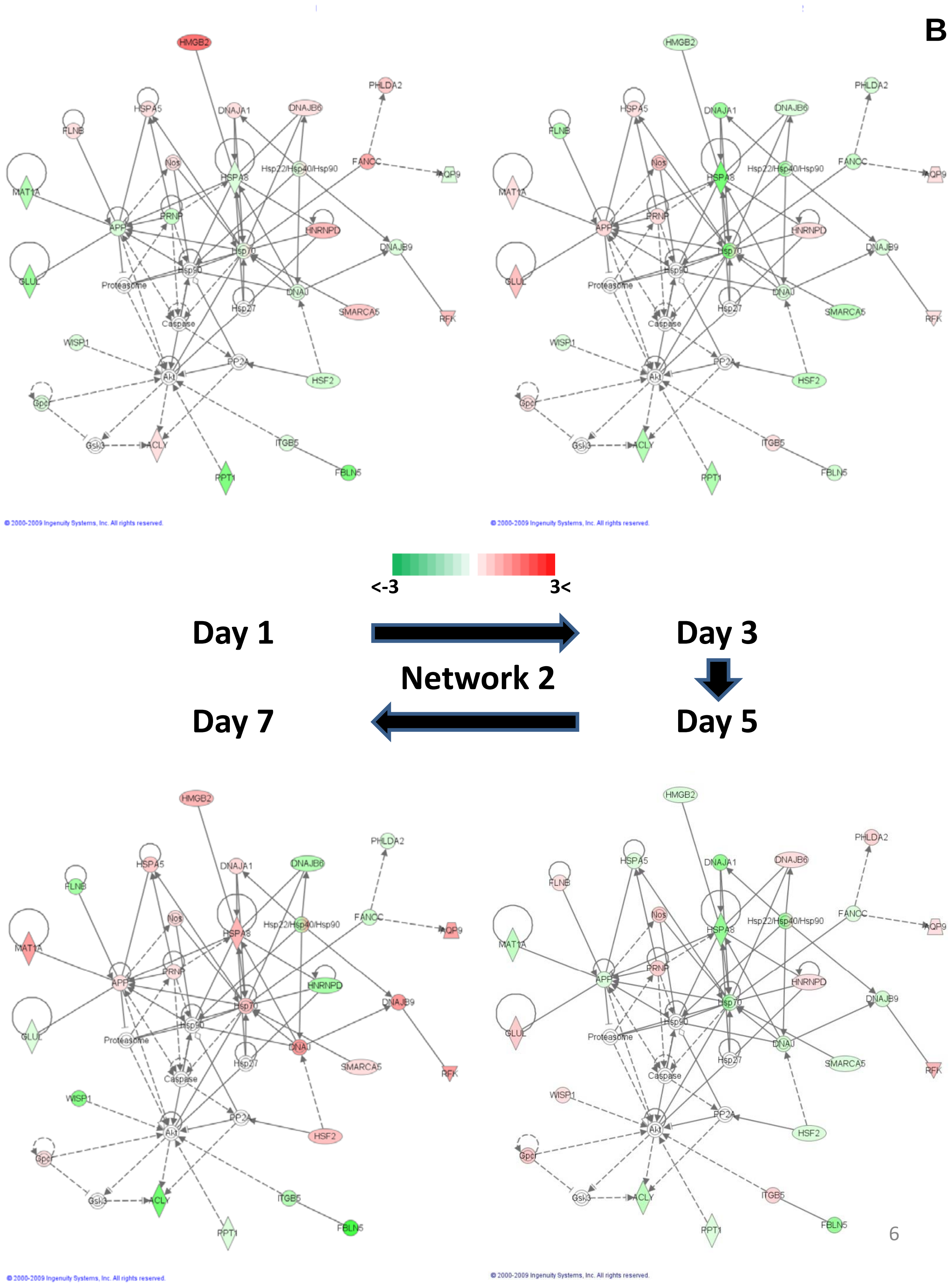

© 2000-2009 Ingenuity Systems, Inc. All rights reserved.

Network 2

B-a

Day 1

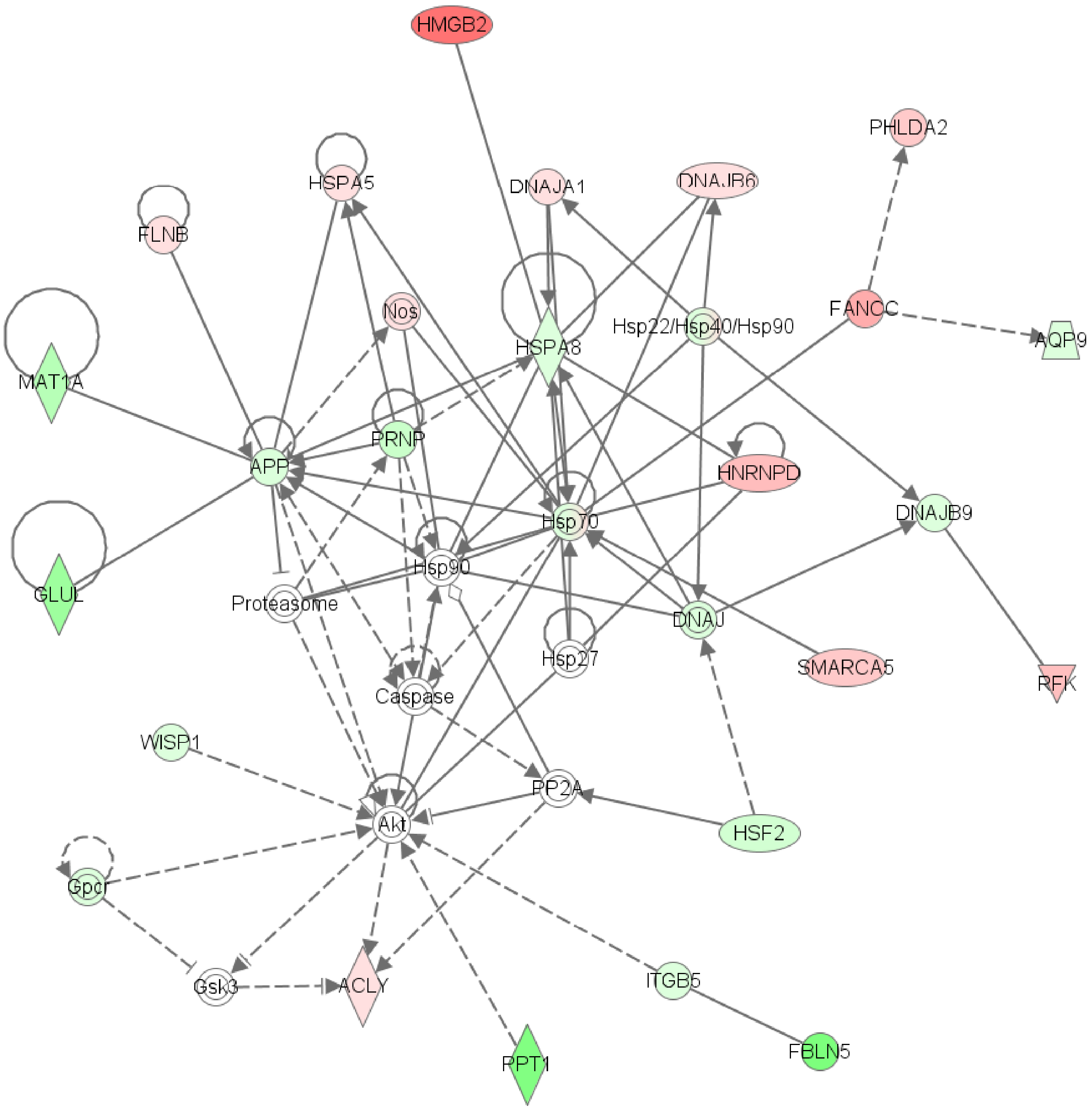

© 2000-2009 Ingenuity Systems, Inc. All rights reserved.

Network 2

B-b

Day 3

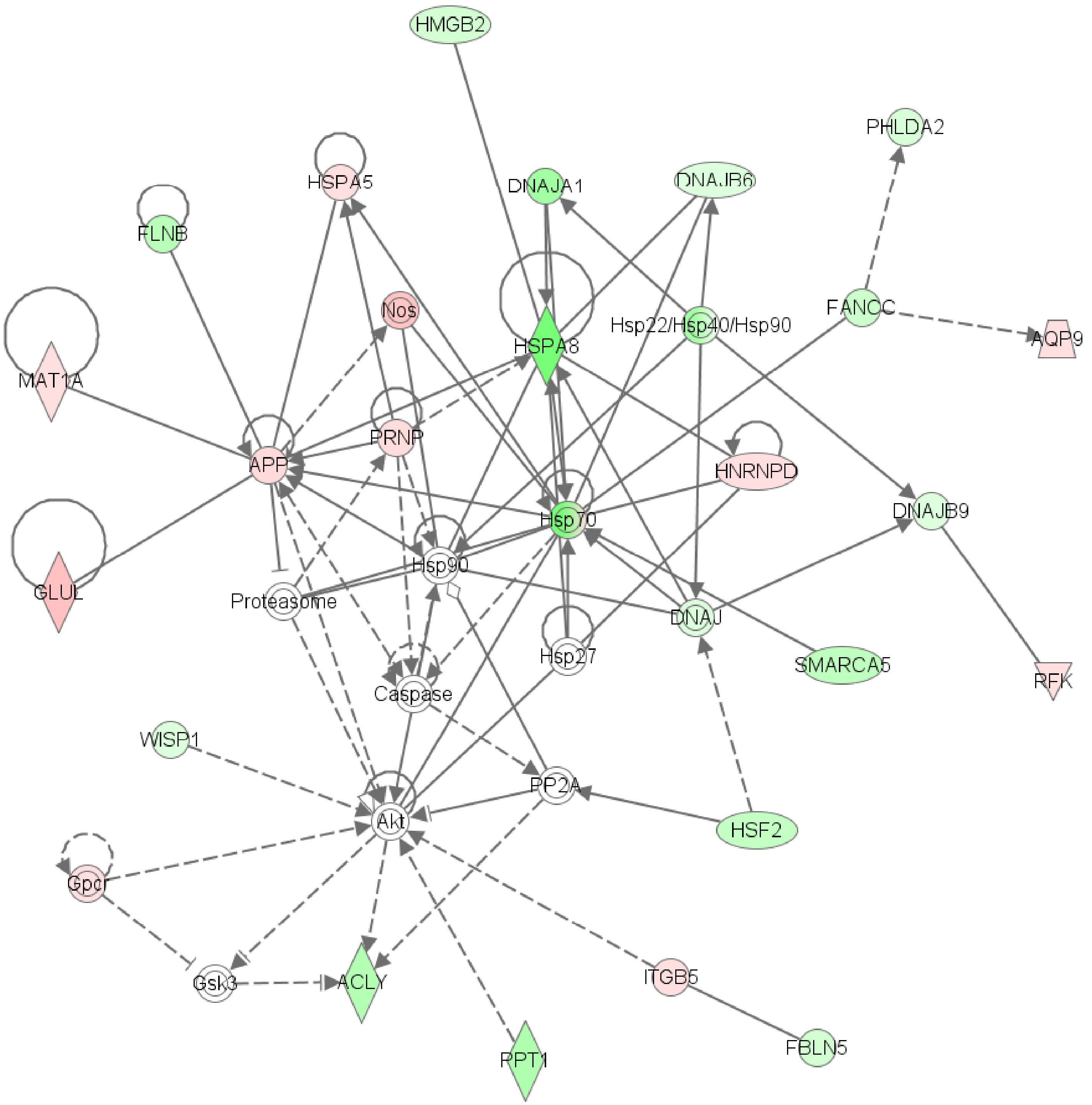

© 2000-2009 Ingenuity Systems, Inc. All rights reserved.

Network 2

B-c

Day 5

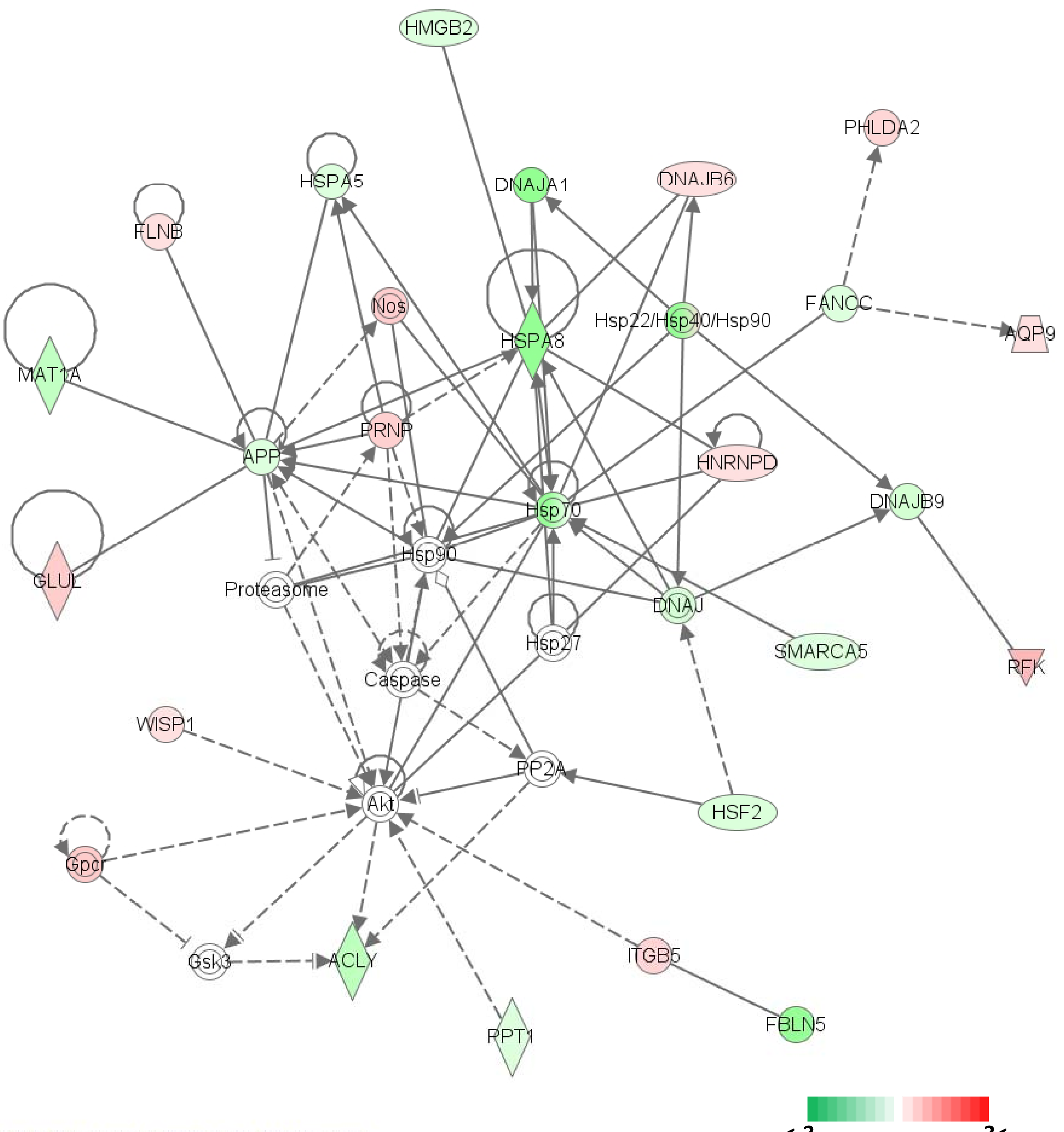

© 2000-2009 Ingenuity Systems, Inc. All rights reserved.

Network 2

B-d

Day 7

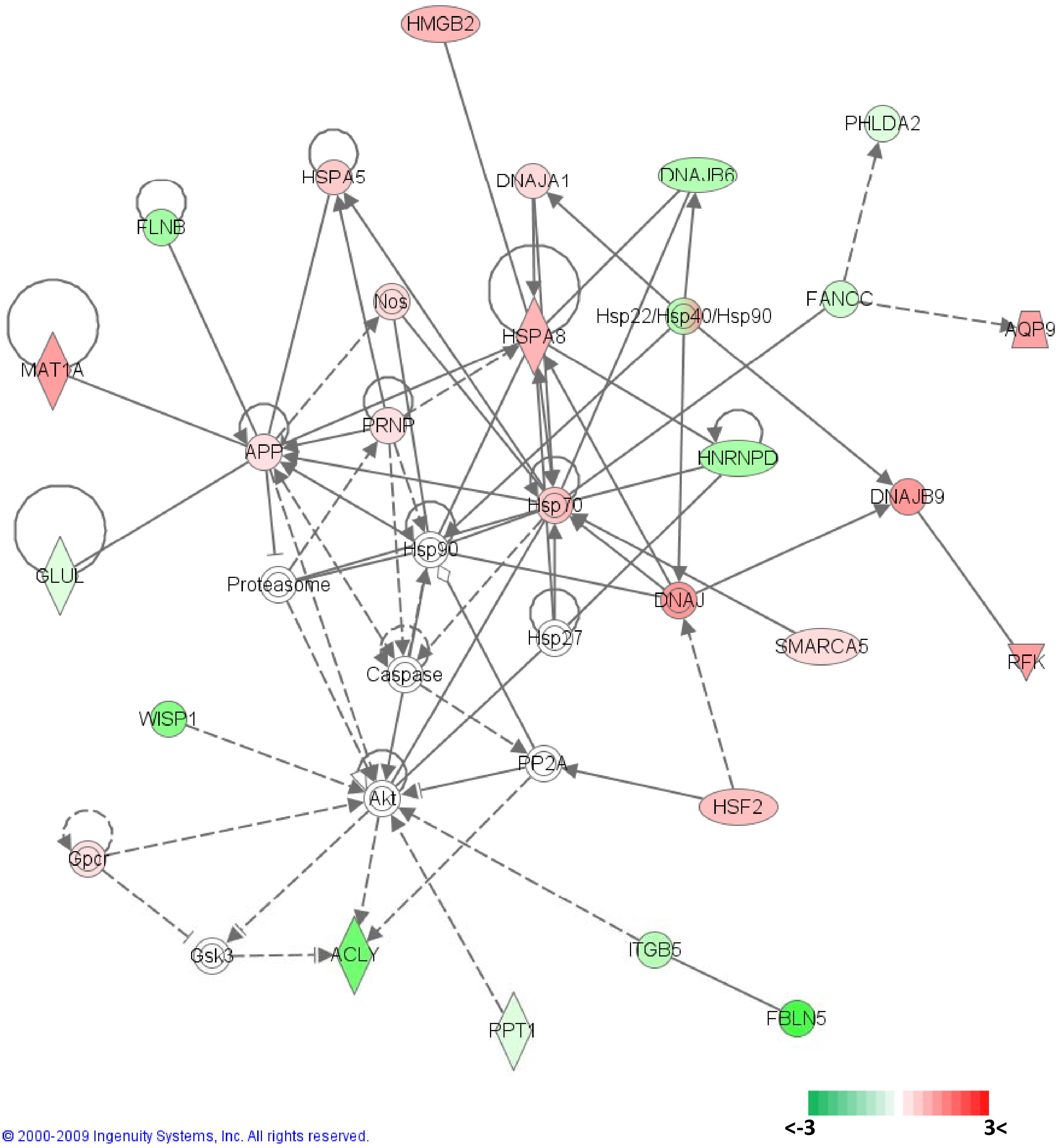

© 2000-2009 Ingenuity Systems, Inc. All rights reserved.

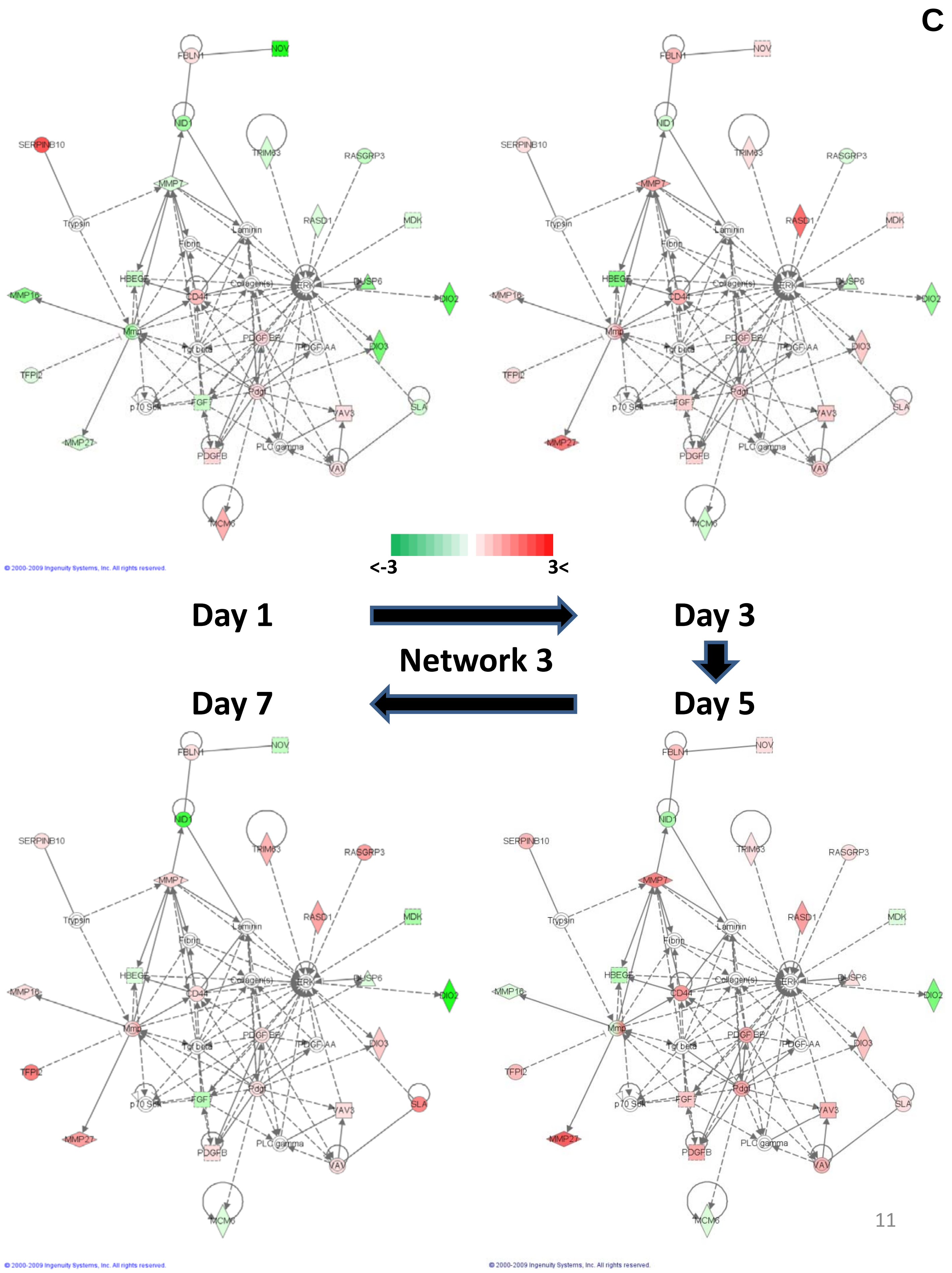
C
FBLN1
NOV
NID1
SERPINB10
TRIM63
RASGRP3
MMP7
Trypsin
Laminin
RASD1
MDK
Fibrin
HBEGF
Collagen(s)
ERK
DUSP6
MMP16
CD44
DIO2
Mmp
PDGF BB
PDGF AA
DIO3
Tgf beta
TFPI2
Pdgf
SLA
p70 S6k
FGF
VAV3
MMP27
PDGFB
PLC gamma
VAV
MCM6
© 2000-2009 Ingenuity Systems, Inc. All rights reserved.
<-3
3<
Day 1
Day 3
Network 3
Day 5
Day 7

Network 3

C-a

Day 1

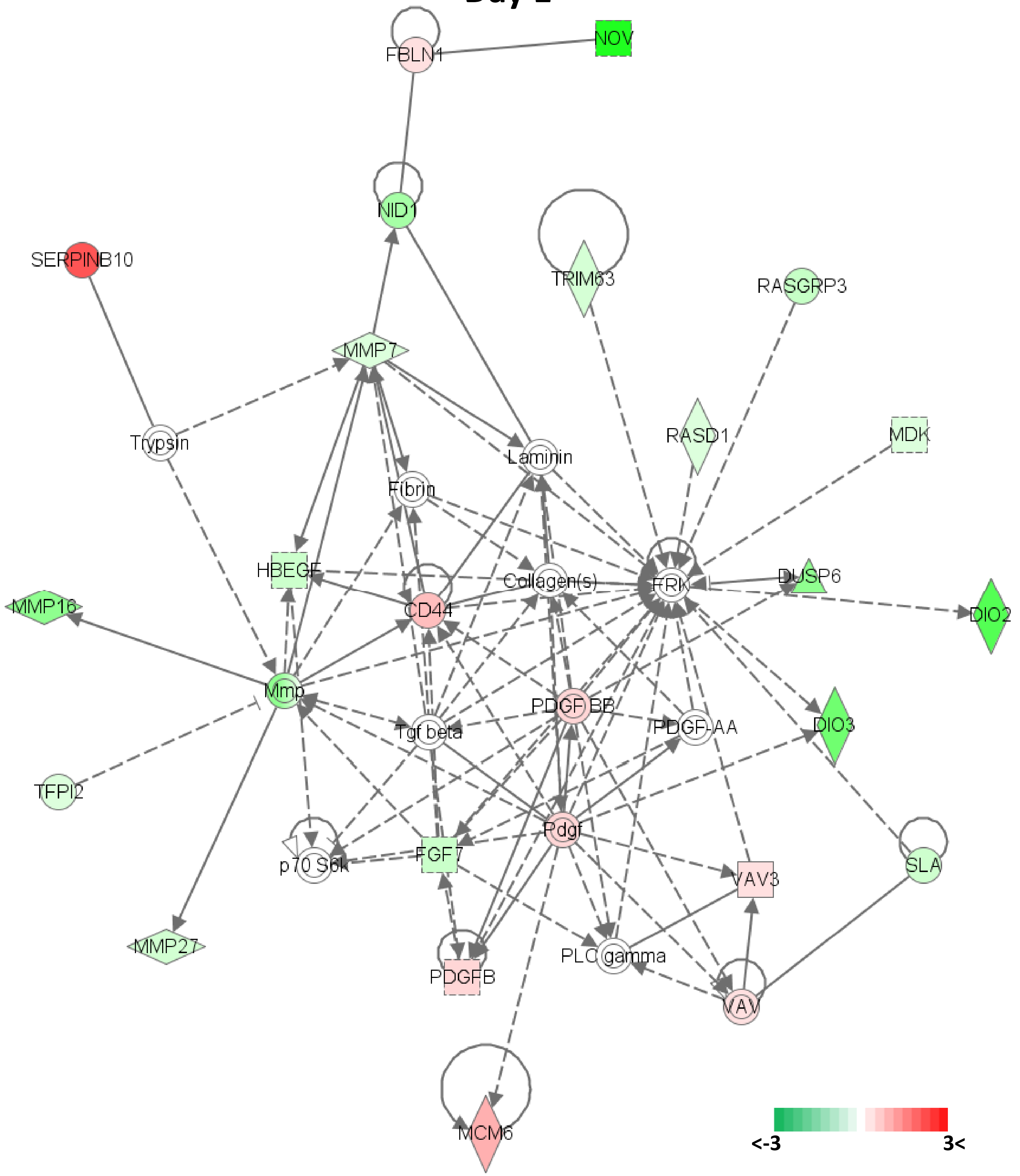

© 2000-2009 Ingenuity Systems, Inc. All rights reserved.

Network 3

C-b

Day 3

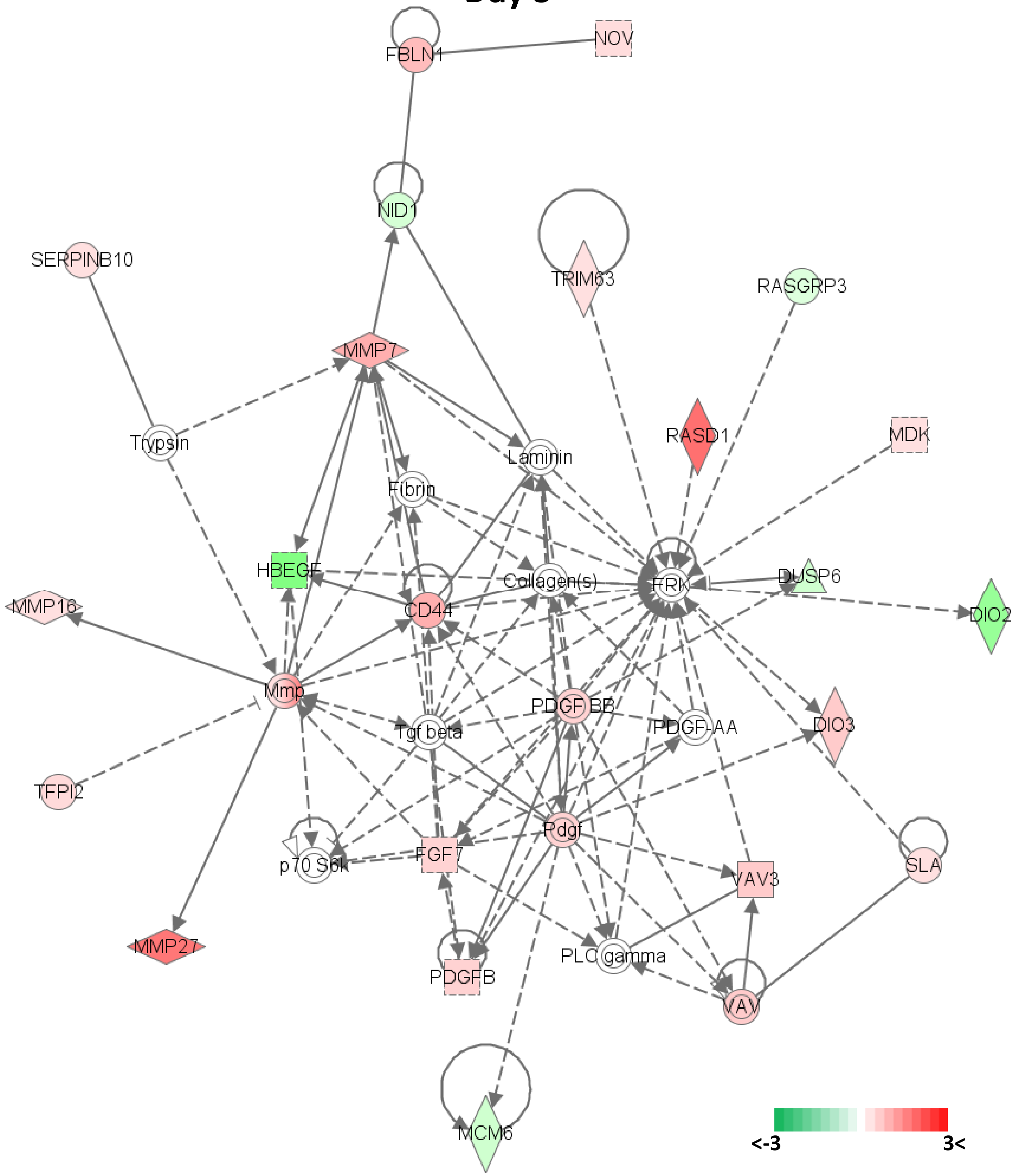

© 2000-2009 Ingenuity Systems, Inc. All rights reserved.

Network 3

C-c

Day 5

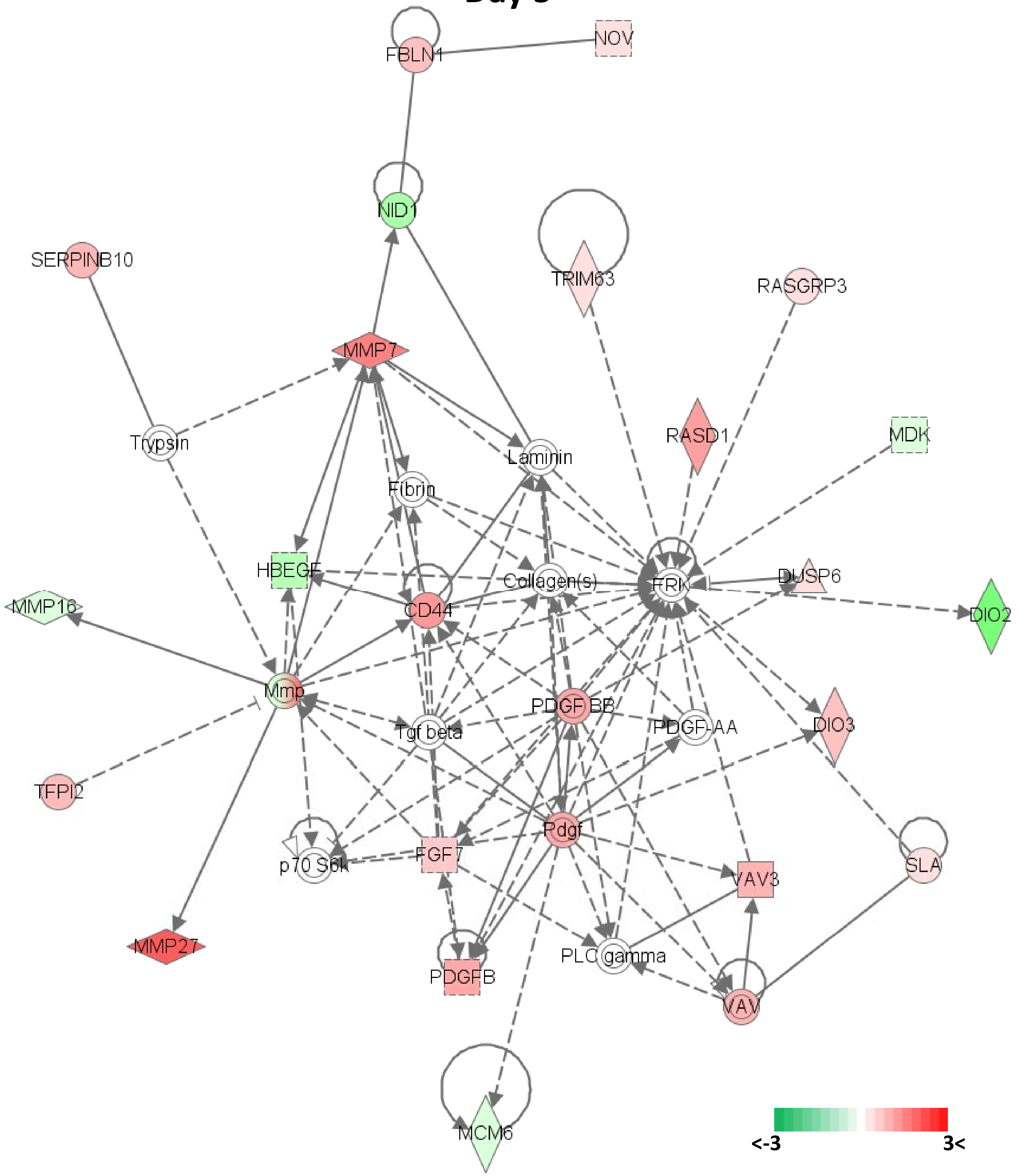

© 2000-2009 Ingenuity Systems, Inc. All rights reserved.

Network 3

C-d

Day 7

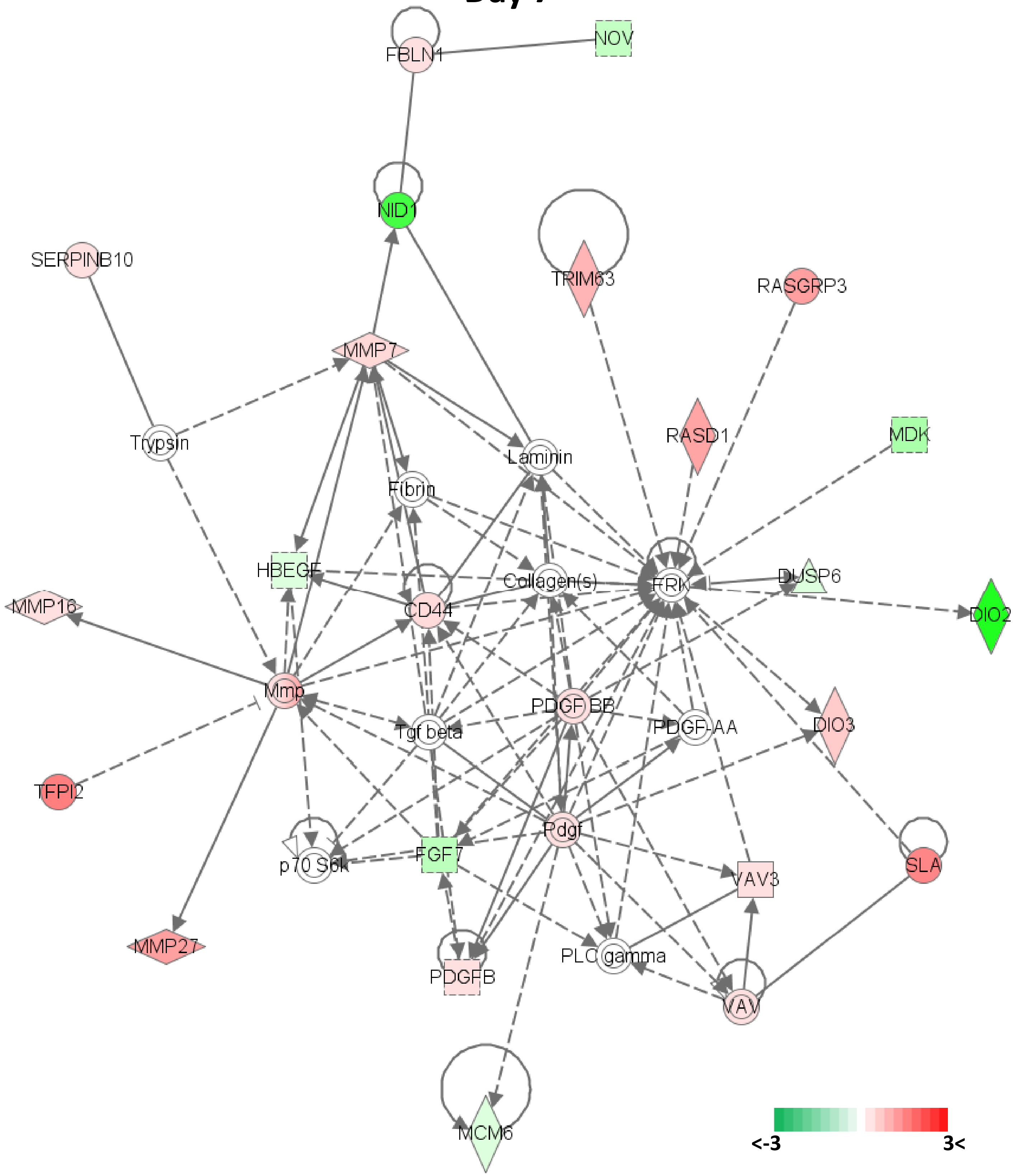

© 2000-2009 Ingenuity Systems, Inc. All rights reserved.

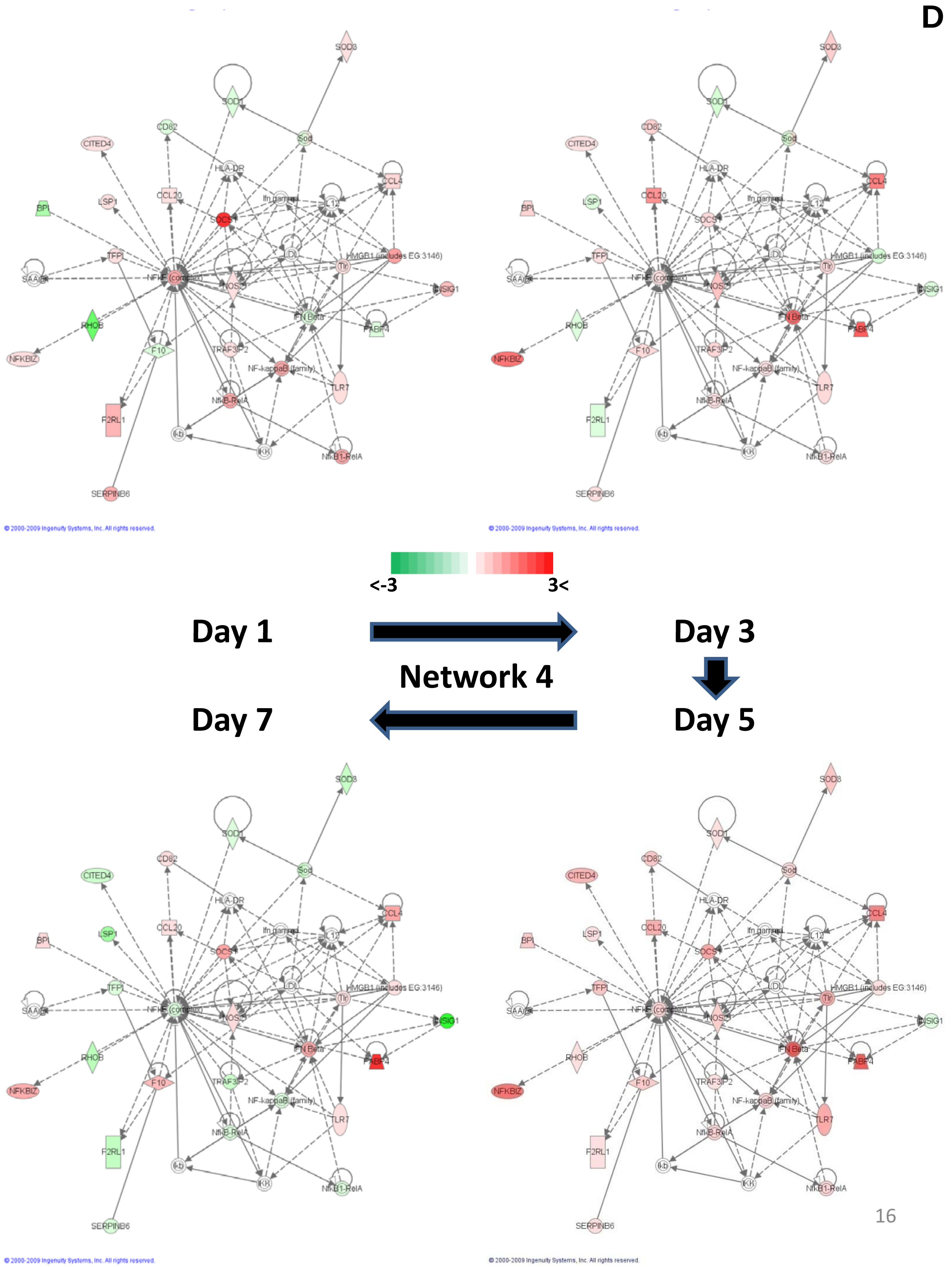
D
SOD3
SOD1
CD82
Sod
CITED4
HLA-DR
CCL4
CCL20
LSP1
Ifn gamma
IL12
BPI
SOCS1
LDL
HMGB1 (includes EG:3146)
TFPI
Tlr
SAA@
NFkB (complex)
NOS2
INSIG1
RHOB
IFN Beta
FABP4
F10
TRAF3IP2
NFKBIZ
NF-kappaB (family)
TLR7
NfkB-RelA
F2RL1
Ikb
IKK
NfkB1-RelA
SERPINB6
© 2000-2009 Ingenuity Systems, Inc. All rights reserved.
<-3
3<
Day 1
Day 3
Network 4
Day 7
Day 5

Network 4

D-a

Day 1

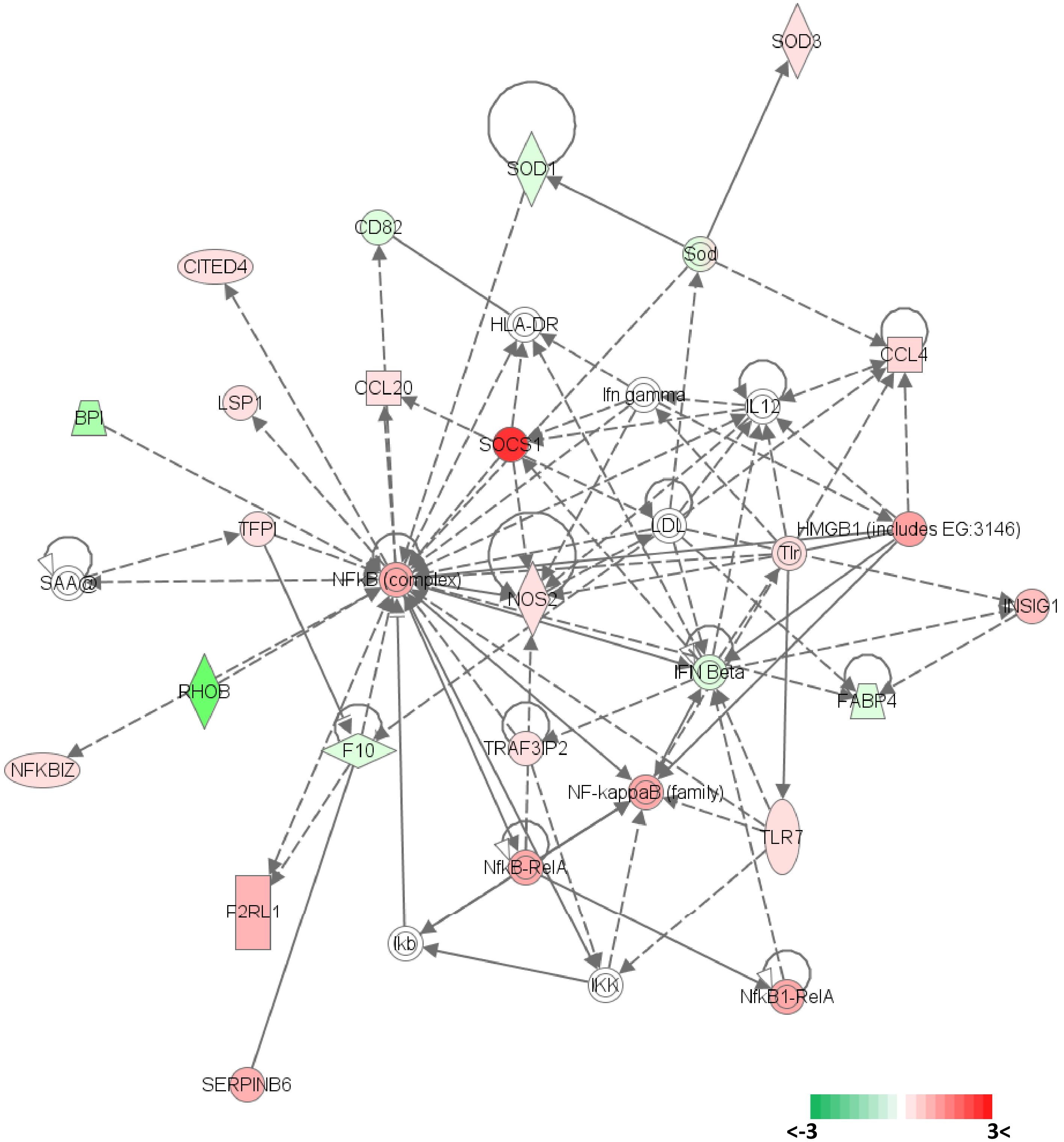

© 2000-2009 Ingenuity Systems, Inc. All rights reserved.

Network 4 D-b

Day 3

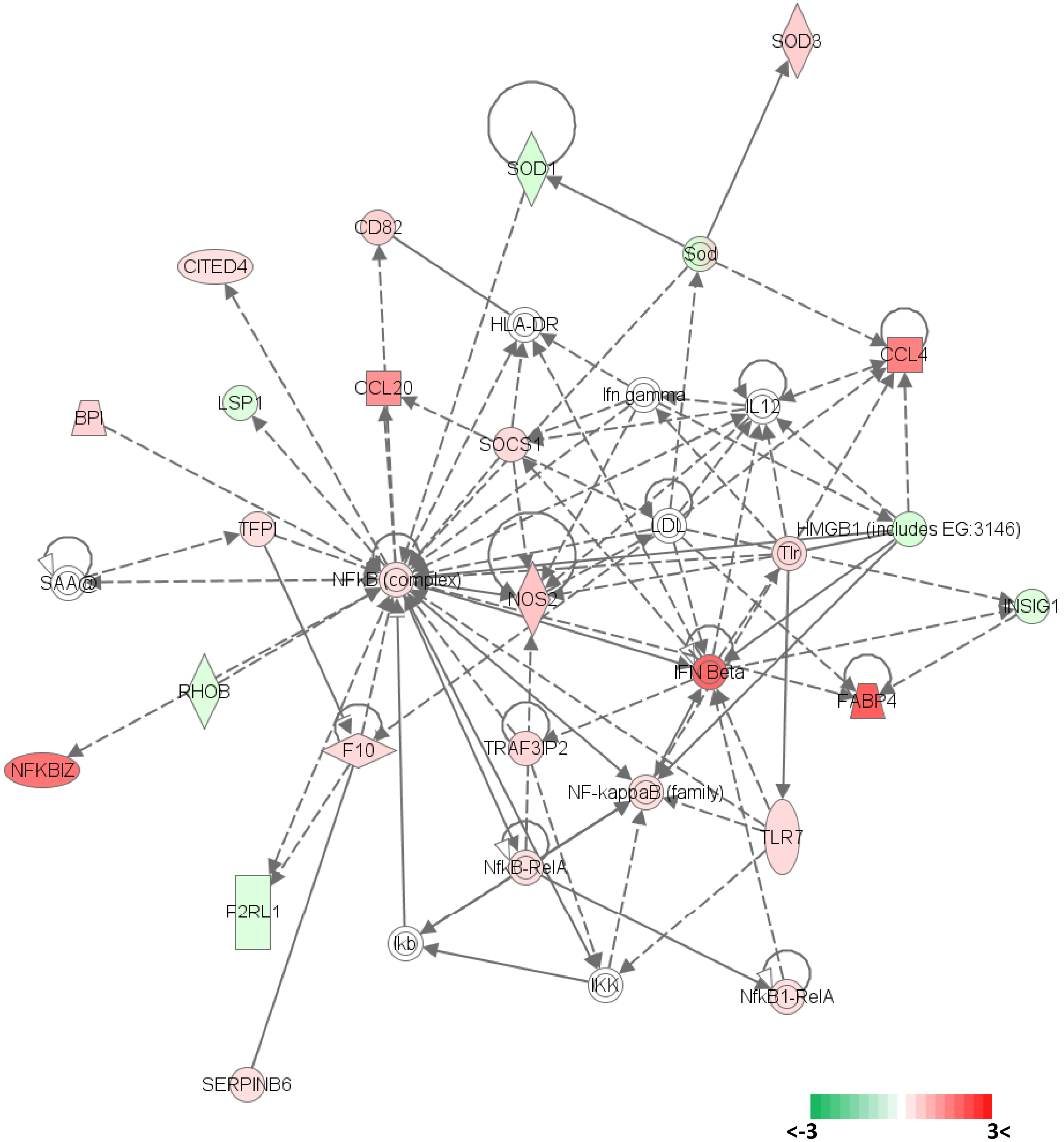

© 2000-2009 Ingenuity Systems, Inc. All rights reserved.

Network 4

D-c

Day 5

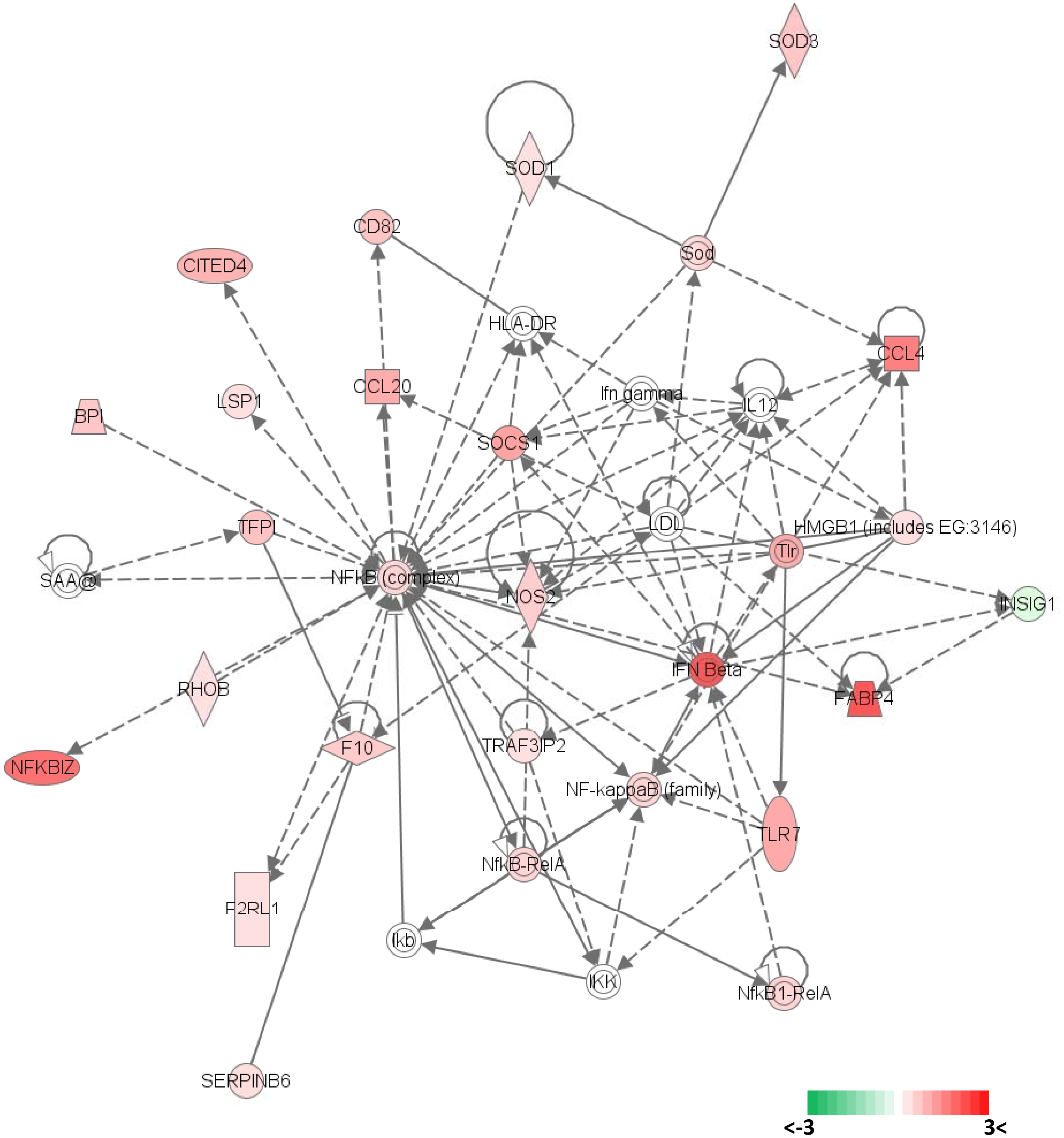

© 2000-2009 Ingenuity Systems, Inc. All rights reserved.

Network 4 D-d

Day 7

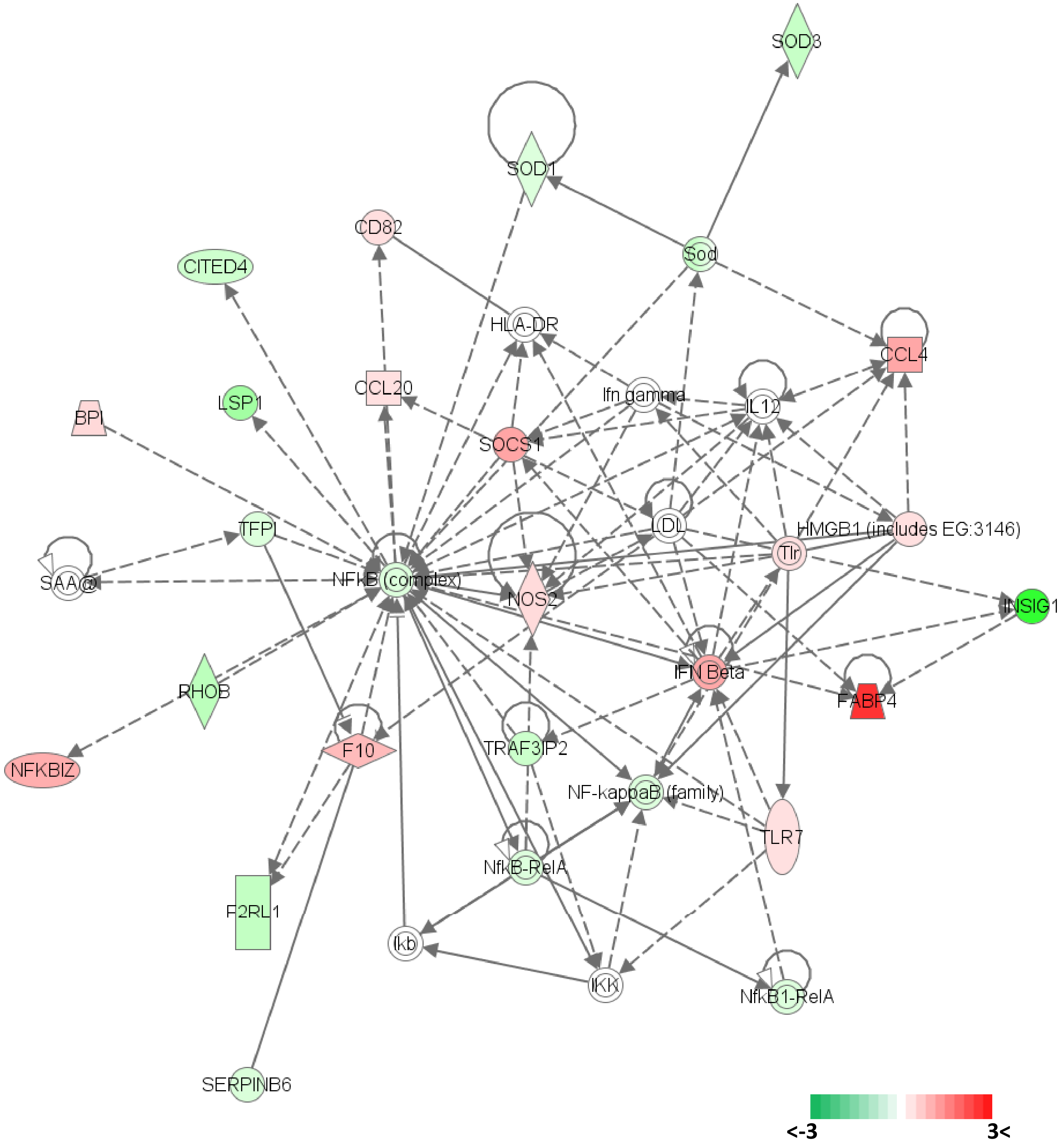

© 2000-2009 Ingenuity Systems, Inc. All rights reserved.

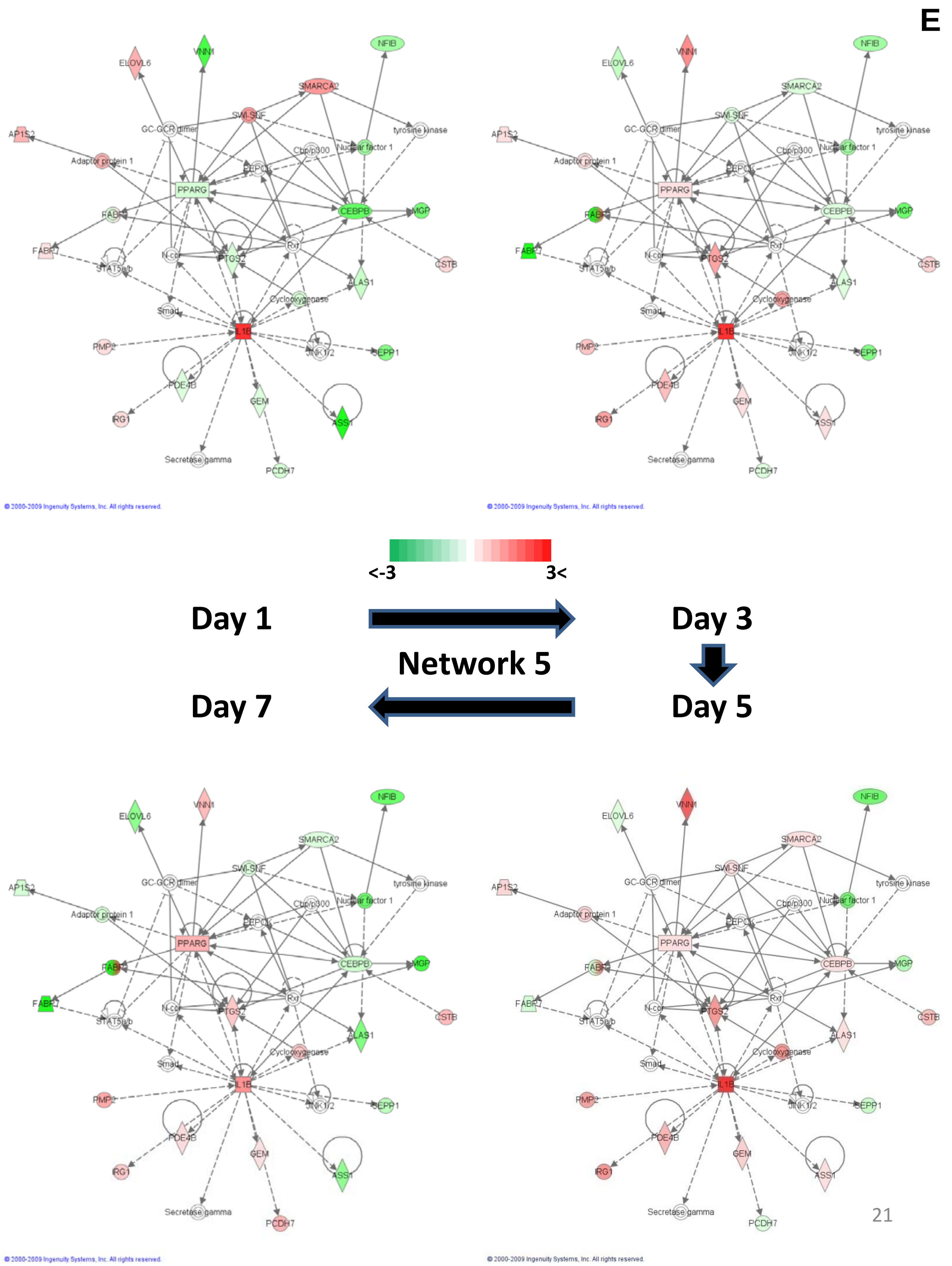

E
NFIB
VNN1
ELOVL6
SMARCA2
SWI-SNF
GC-GCR dimer
tyrosine kinase
AP1S2
Adaptor protein 1
Cbp/p300
Nuclear factor 1
PEPCK
PPARG
CEBPB
MGP
FABP
FABP7
Rxr
N-cor
PTGS2
STAT5a/b
CSTB
ALAS1
Cyclooxygenase
Smad
IL1B
PMP2
JNK1/2
SEPP1
PDE4B
GEM
IRG1
ASS1
Secretase gamma
PCDH7
© 2000-2009 Ingenuity Systems, Inc. All rights reserved.
<-3
3<
Day 1
Day 3
Network 5
Day 5
Day 7

Network 5

E-a

Day 1

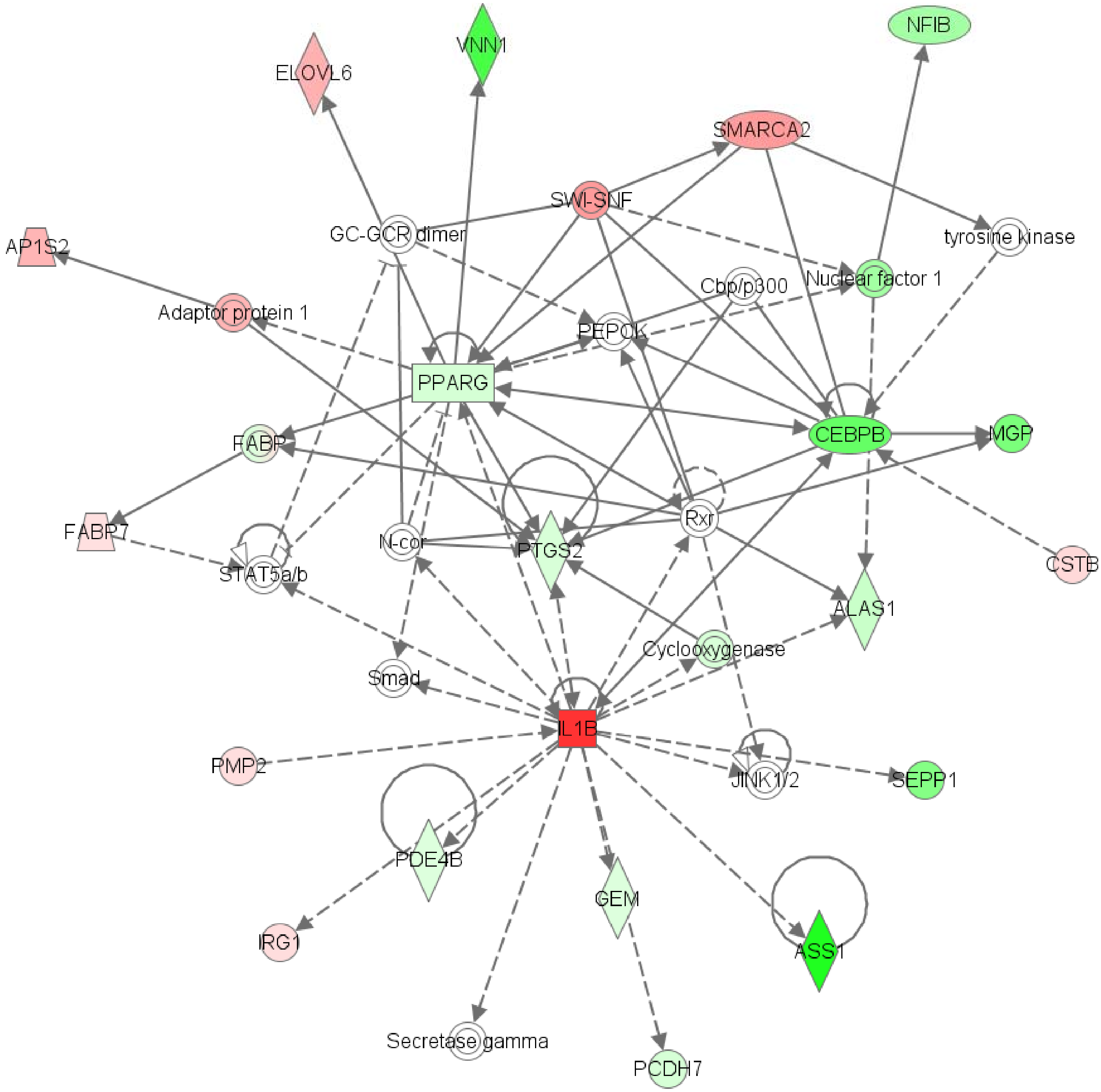

© 2000-2009 Ingenuity Systems, Inc. All rights reserved.

Network 5

E-b

Day 3

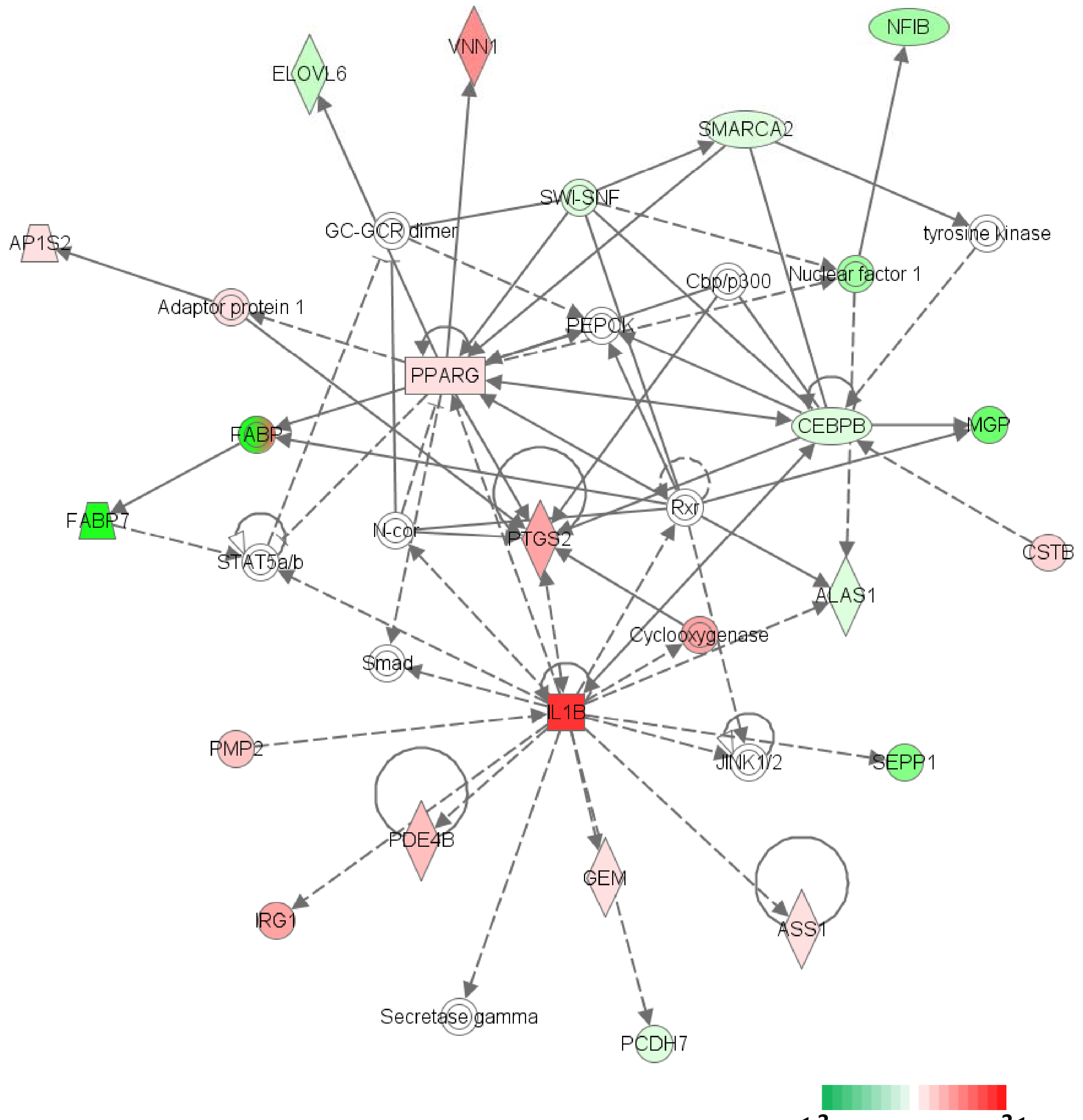

© 2000-2009 Ingenuity Systems, Inc. All rights reserved.

Network 5

E-c

Day 5

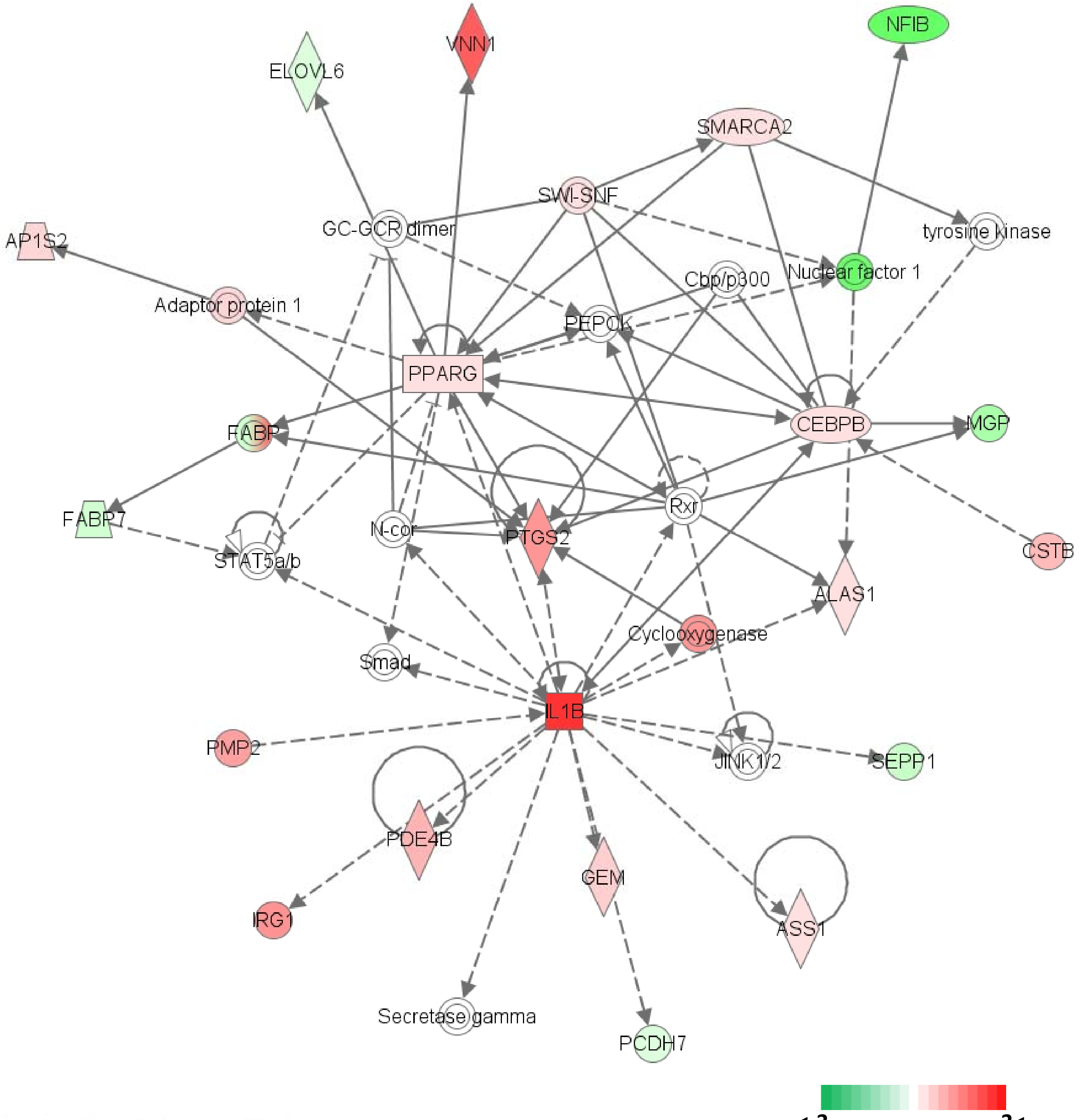

© 2000-2009 Ingenuity Systems, Inc. All rights reserved.

Network 5

E-d

Day 7

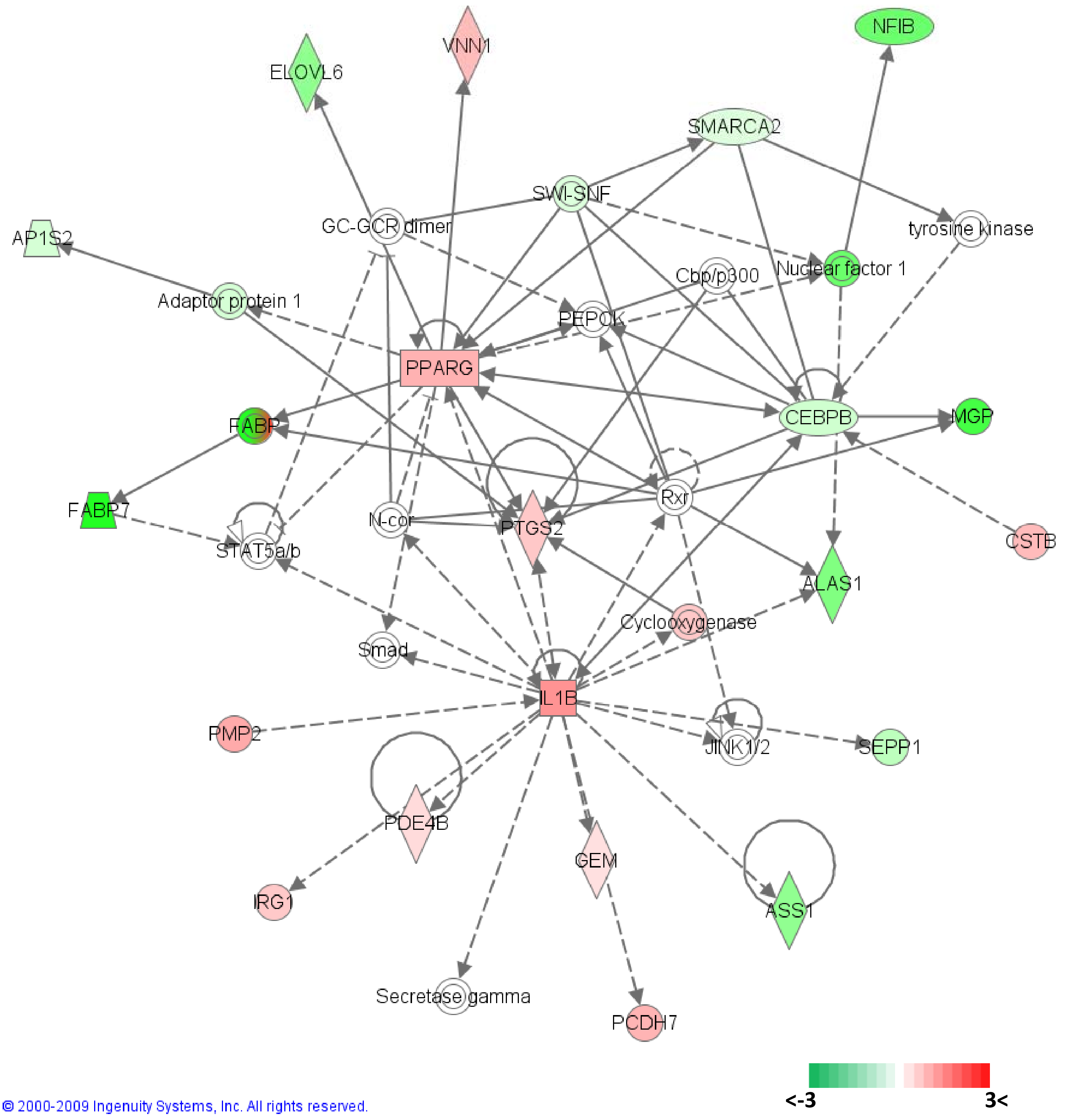

© 2000-2009 Ingenuity Systems, Inc. All rights reserved.

F

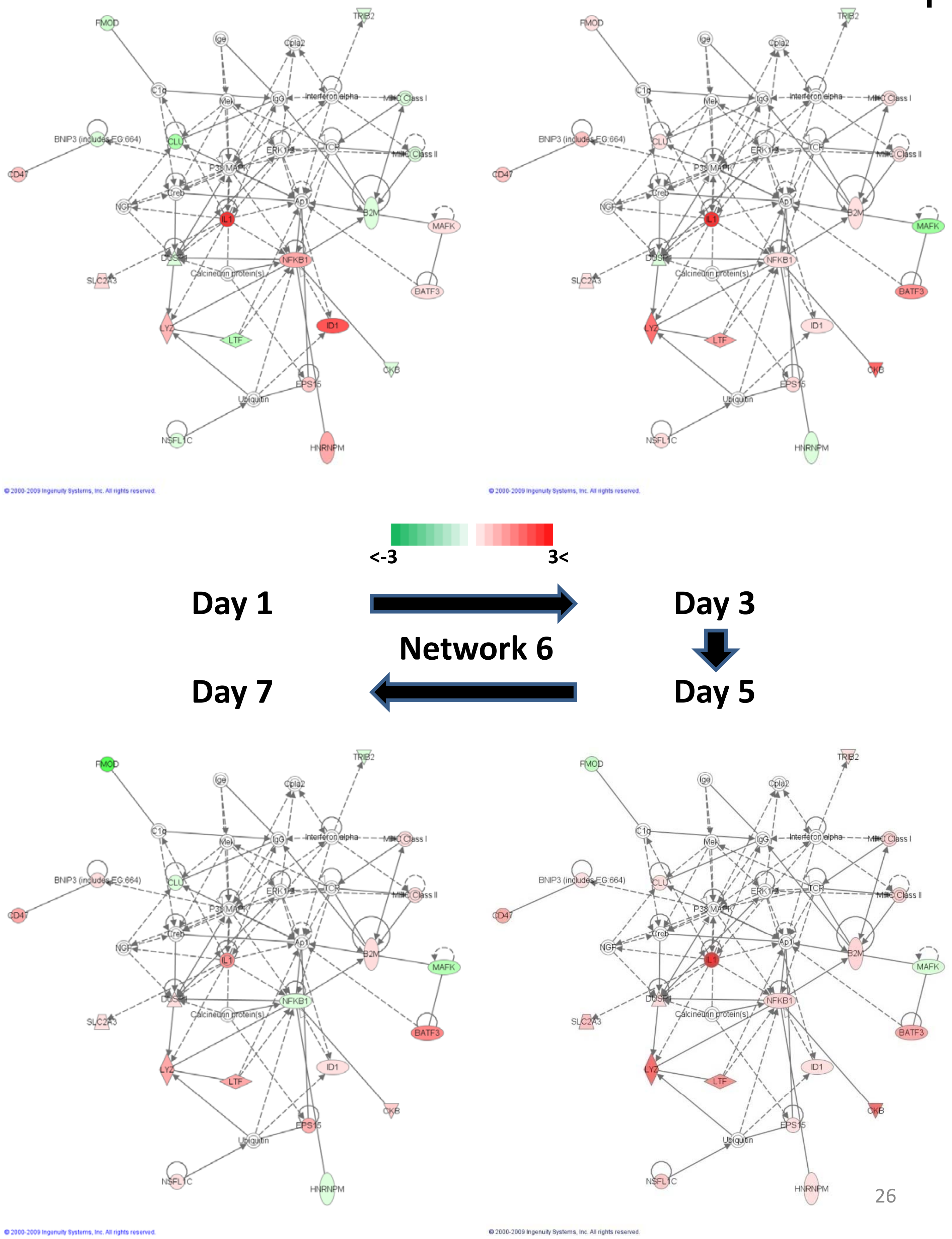
FMOD
TRIB2
Ige
Cpla2
C1q
Mek
IgG
Interferon alpha
MHC Class I
BNP3 (includes EG:664)
CLU
ERK1/2
TCR
MHC Class II
CD47
P38 MAPK
Creb
Ap1
NGF
IL1
B2M
MAFK
DUSP1
NFKB1
Calcineurin protein(s)
SLC2A3
BATF3
LYZ
ID1
LTF
CKB
EPS15
Ubiquitin
NSFL1C
HNRNPM
© 2000-2009 Ingenuity Systems, Inc. All rights reserved.
<-3
3<
Day 1
Day 3
Network 6
Day 7
Day 5

Network 6 F-a

Day 1

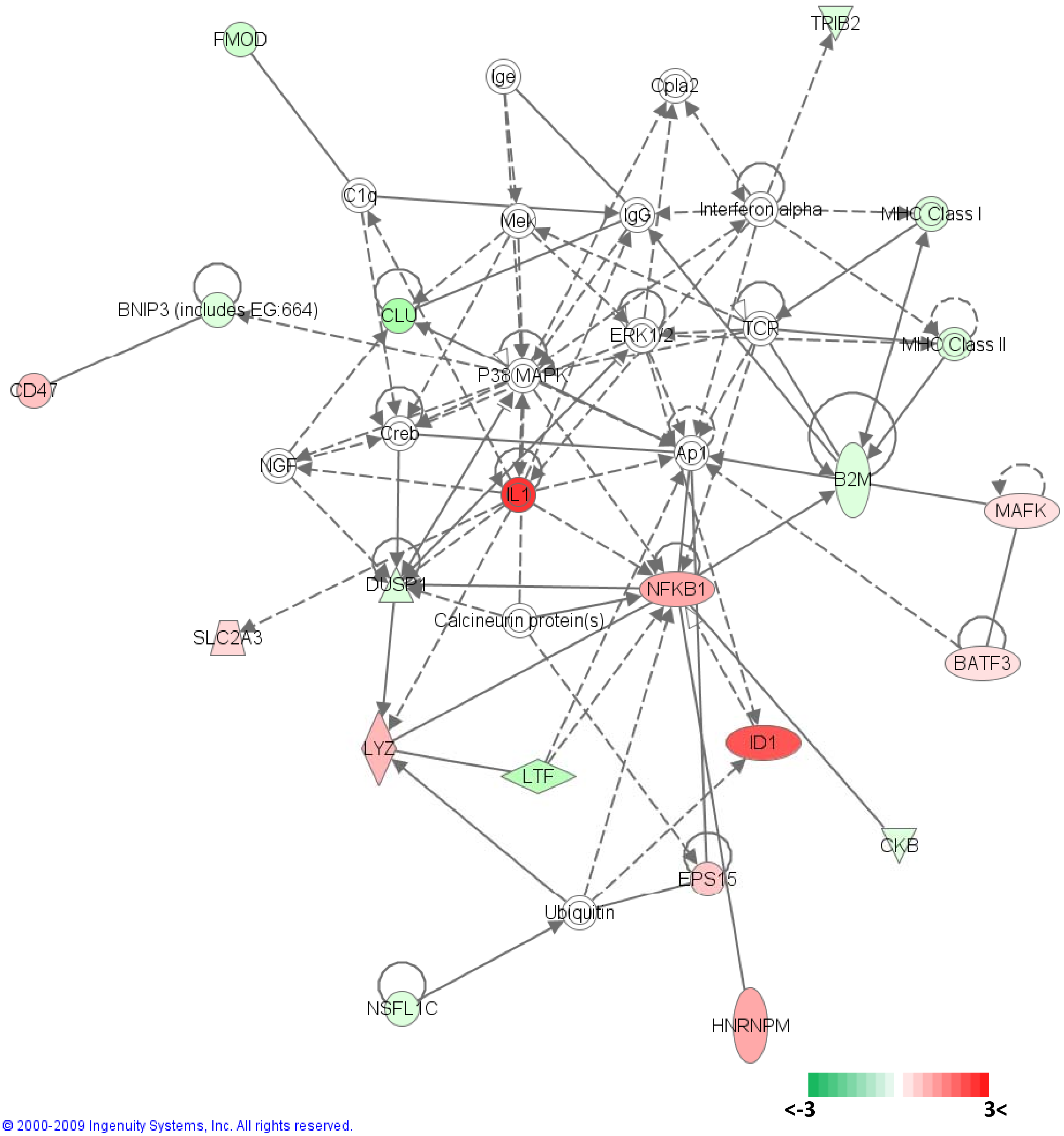

© 2000-2009 Ingenuity Systems, Inc. All rights reserved.

Network 6

F-b

Day 3

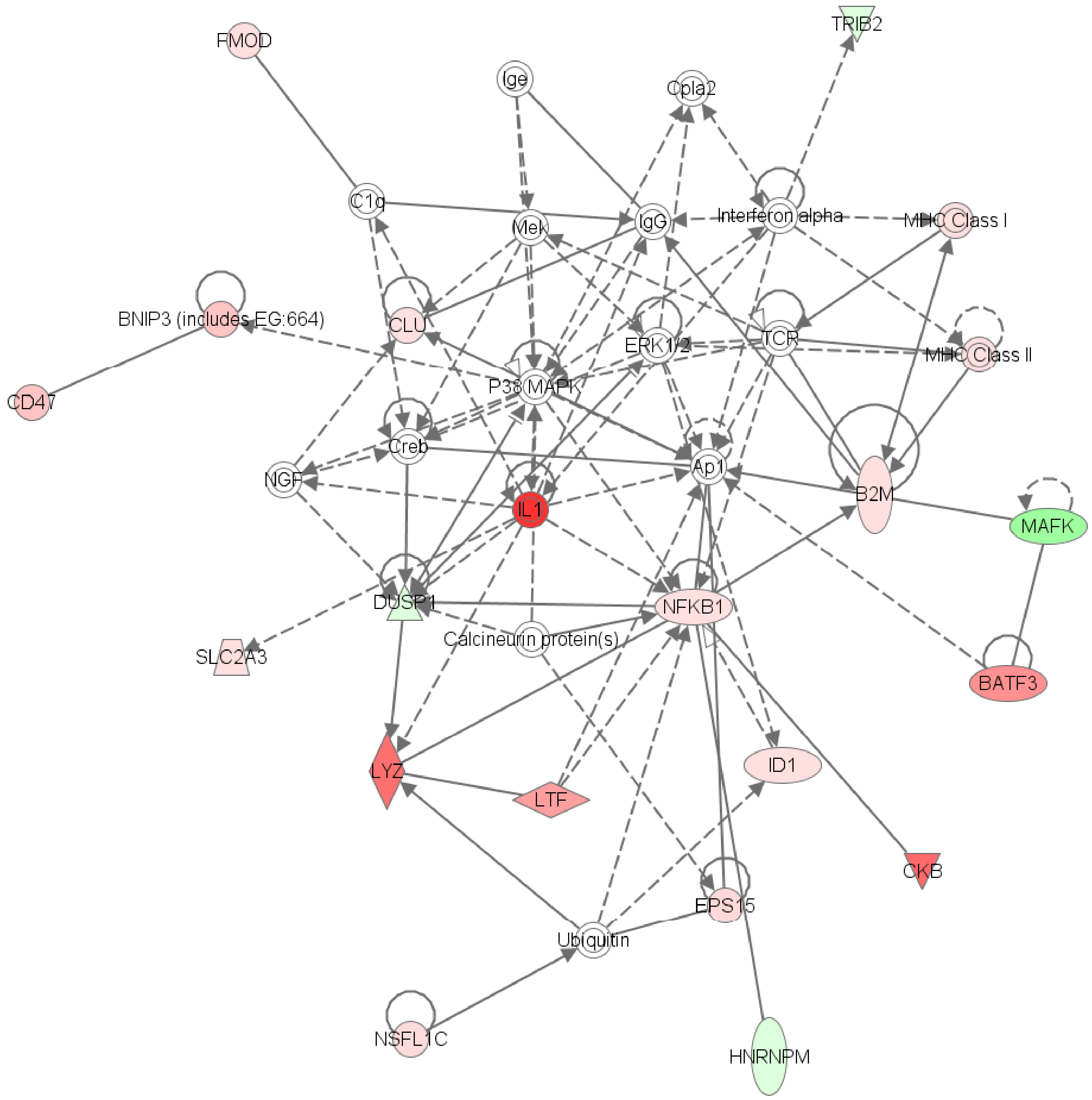

© 2000-2009 Ingenuity Systems, Inc. All rights reserved.

Network 6

F-c

Day 5

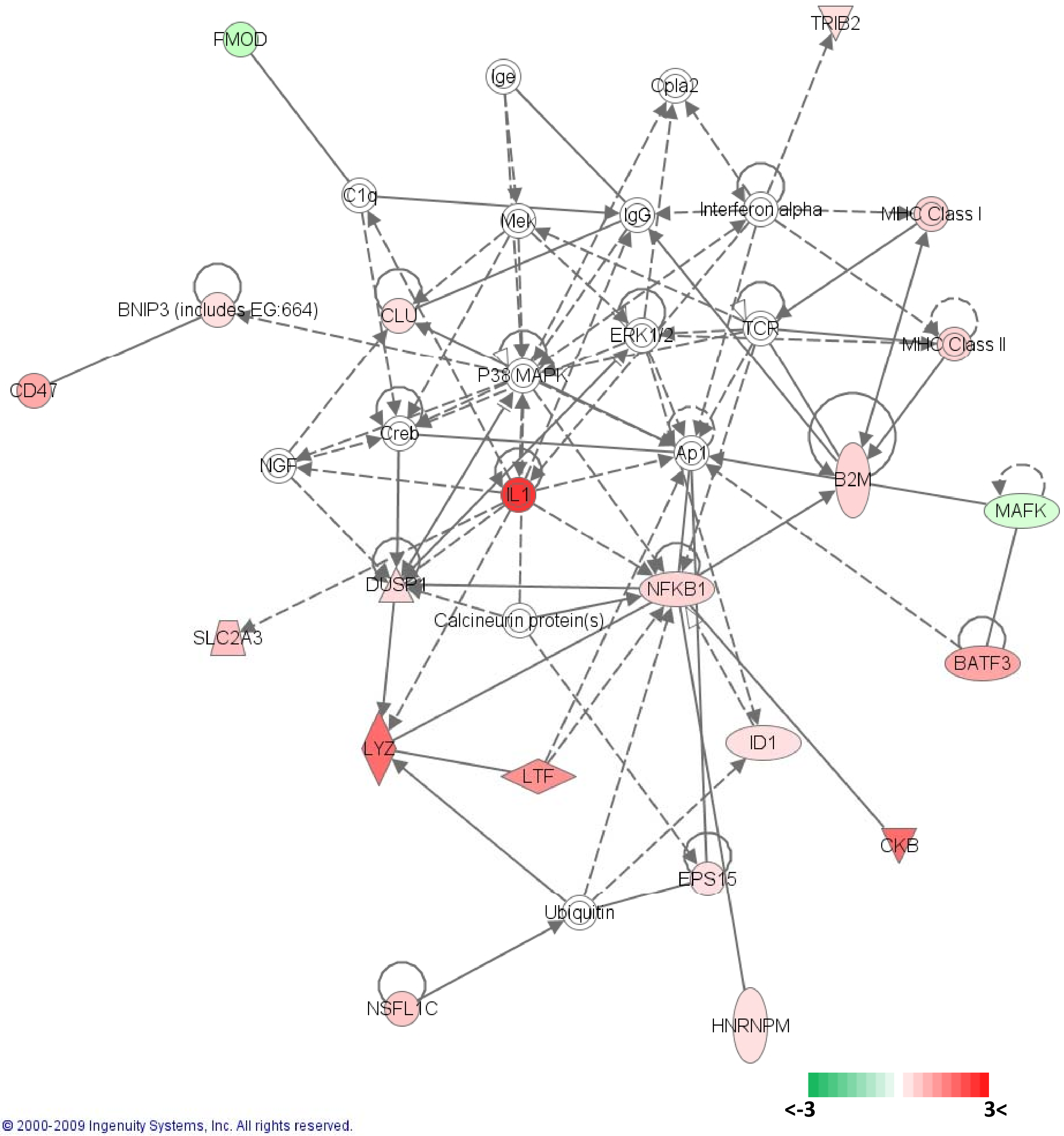

© 2000-2009 Ingenuity Systems, Inc. All rights reserved.

Network 6

F-d

Day 7

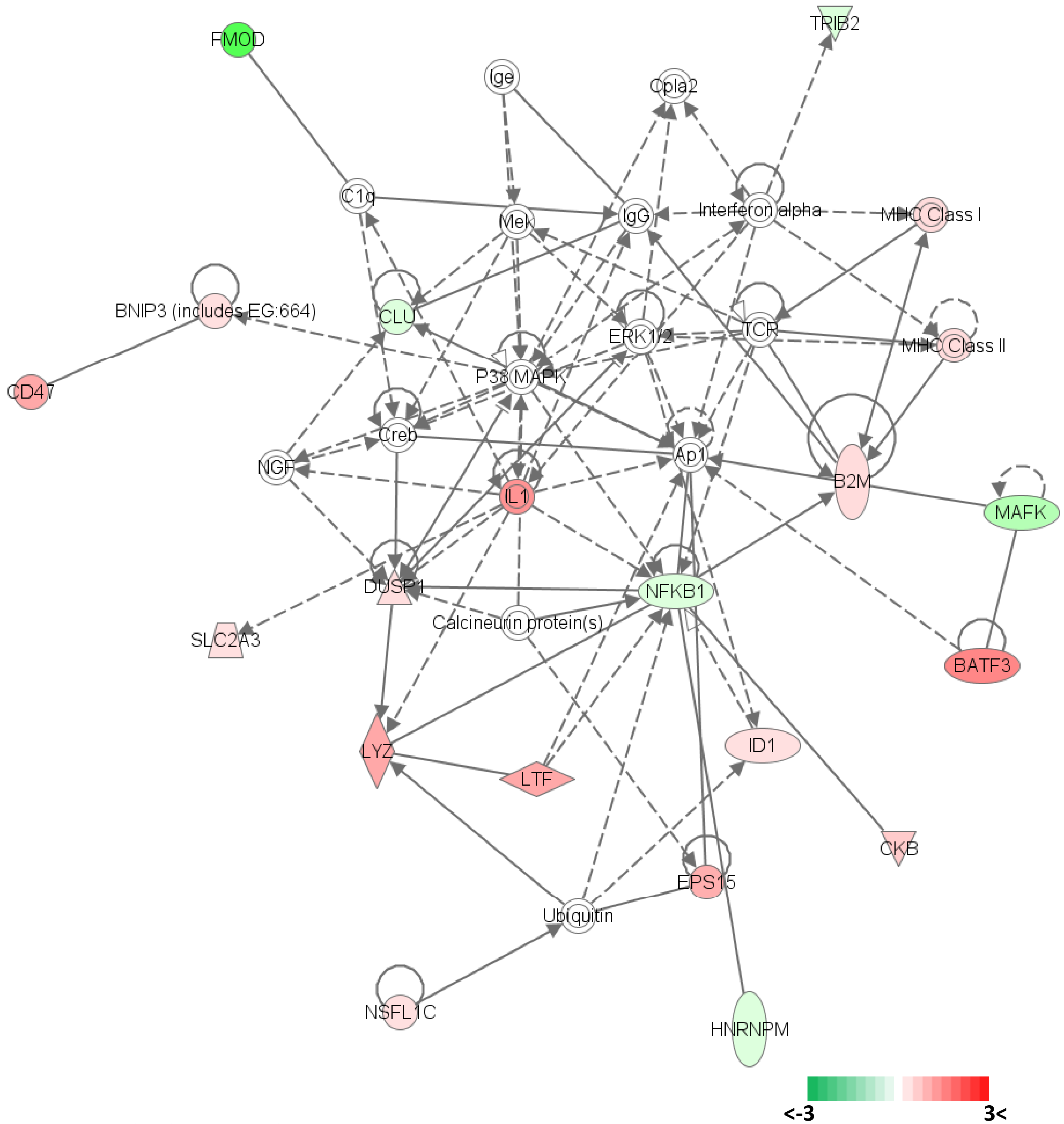

© 2000-2009 Ingenuity Systems, Inc. All rights reserved.
